# Seven New Drimane-Type Sesquiterpenoids from a Marine-Derived Fungus *Paraconiothyrium sporulosum* YK-03

**DOI:** 10.3390/molecules24091817

**Published:** 2019-05-10

**Authors:** Li-Hua Zhang, Gang Chen, Yi Sun, Hai-Feng Wang, Jiao Bai, Hui-Ming Hua, Yue-Hu Pei

**Affiliations:** 1 Department of Medicinal Chemistry and Natural Medicine Chemistry, College of Pharmacy, Harbin Medical University, Harbin 150081, China; 2 School of Traditional Chinese Materia Medica, Shenyang Pharmaceutical University, Shenyang 110016, China; zhanglihua200061@163.com (L.-H.Z.); chengang1152001@hotmail.com (G.C.); wanghaifeng0310@163.com (H.-F.W.); baijiao@hotmail.com (J.B.); huimhua@163.com (H.-M.H.); 3 State Key Laboratory of Bioactive Substance and Function of Natural Medicines, Institute of Materia Medica, Chinese Academy of Medical Sciences & Peking Union Medical College, Beijing 100050, China; 4 Institute of Chinese Materia Medica, China Academy of Chinese Medical Sciences, Beijing 100700, China; sylotus@hotmail.com

**Keywords:** *Paraconiothyrium*, *Paraconiothyrium sporulosum*, drimane-type sesquiterpenoid, sporulositol, *seco*-sporulositol, sporuloside

## Abstract

Seven new drimane-type sesquiterpenoids, namely the sporulositols **A**–**D** (**1**–**4**), 6-hydroxydiaporol (**5**), *seco*-sporulositol (**6**) and sporuloside (**7**) were isolated from the ethyl acetate extract of fermentation broth for a marine-derived fungus *Paraconiothyrium sporulosum* YK-03. Their structures were elucidated by analysis of extensive spectroscopic data, and the absolute configurations were established by crystal X-ray diffraction analysis and comparisons of circular dichroism data. Among them, sporulositols **A**–**E** (**1**–**4**) and *seco*-sporulositol (**6**) represent the first five examples of a unique class of drimanic mannitol derivatives, while compounds **6** and **7** may represent two new series of natural drimanes, possessing an aromatic ring with a rare 4,5-secodrimanic skeleton and an unusual CH_3_-15 rearranged drimanic α-D-glucopyranside, respectively. Furthermore, the origin of mannitol moiety was investigated by reliable HPLC and NMR analyses.

## 1. Introduction

Marine fungi afforded chemically diverse and pharmacologically active metabolites, and have become a remarkable source of marine drugs [1,2,3,4]. *Paraconiothyrium* genus is a new genus specified by Verkley et al. through analysis of 18S rRNA and ITS sequences [5], and has been known as a plant pathogen similar to *Phoma* and rarely as a human pathogen [6]. Verkley proposed that two *Coniothyrium* species (*C. fuckelii* and *C. sporulosum*) should be combined into the genus *Paraconiothyrium*, and united as *P. sporulosum* [5,6]. So far, structural diverse terpenoids including brasilamides A–N [7,8,9], hawaiinolides A–G [10,11], epoxyphomalins A–E [12], sporulaminals A–B [13] and six isopimarane diterpenoid glycosides [14], and several polyketides [15,16,17,18,19,20,21,22,23,24,25,26] have been identified from the genus *Paraconiothyrium*.

Drimanes, a type of sesquiterpenoid with a bicyclic scaffold, are widely distributed in plants, liverworts, fungi and certain marine organisms (sponges), possessing diverse structural features [27,28] and extensive biological activities, such as antibacterial [29,30], antifungal [31,32], antiviral [29,33], cytotoxic [34,35], antifeedant [36,37], plant-growth [38,39], and so on. Natural rearranged drimanes only occurred with a 1,2 methyl shift from C-4 to C-3 [40]. In 2013, the first drimanic compound with an aromatic ring was synthesized [41], while a synthesized *seco*-drimanic compound was reported for the first time in 2016 [42]. Because of their interesting structural features and bioactivities, they have attracted increasing attention of biologists and chemists for further research [36,41,43].

**Figure 1:**
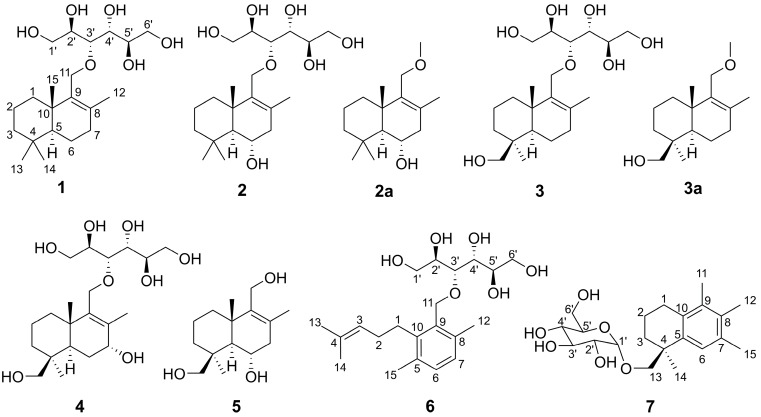
The structures of **1**–**7**.

In course of our continuous exploration for novel marine natural products, seven new drimane-type sesquiterpenoids (Figure 1), namely sporulositols **A**–**D** (**1**–**4**), 6-hydroxydiaporol (**5**), *seco*-sporulositol (**6**) and sporuloside (**7**), were isolated from the ethyl acetate extract of a fermentation broth of a marine-derived fungus *P. sporulosum* YK-03 (Genbank Accession Number KC416199), which was collected from the sea mud in the intertidal zone of Bohai Bay (Liaoning Province, China). Among them, sporulositols **A**–**D** (**1**–**4**) and *seco*-sporulositol (**6**) represent the first five examples of unique drimanic mannitol derivatives, *seco*-sporulositol (**6**) possesses a 4,5-secodrimanic skeleton, and sporuloside (**7**) is a CH_3_-15 rearrangement derivative of drimanic glucoside. Compounds **6** and **7** may represent two new series of natural drimanes with an aromatic ring (compound **6**), or a drimane-type sesquiterpene glycoside with an *α*-D-glucose moiety (compound **7**). Herein, we report the isolation and structure elucidation of the isolated compounds, together with the origin of mannitol moiety.

## 2. Results and Discussion

The fermentation broth of *P. sporulosum* YK-03 was concentrated and extracted with ethyl acetate and *n*-butanol, successively. Then the ethyl acetate extract of the fermentation broth of *P. sporulosum* YK-03 was subjected to various modern chromatographic isolation methods (including silica gel/Sephadex LH-20 column chromatography and reversed-phase C_18_ preparative high performance liquid chromatography) to give seven new compounds **1**−**7** (4.3 mg, 93.3 mg, 89.8 mg, 2.6 mg, 2.3 mg, 2.5 mg and 2.0 mg). Their structures and the absolute configurations were elucidated by analysis of HRESIMS, 1D/2D NMR, circular dichroism (CD) and X-ray diffraction analyses.

### 2.1. Structural Elucidation

Sporulositols **A**–**D** (**1**–**4**) and *seco*-sporulositol (**6**) were isolated as colorless oils. Their respective molecular formulas of C_21_H_38_O_6_, C_21_H_38_O_7_, C_21_H_38_O_7_, C_21_H_38_O_8_, and C_21_H_34_O_6_ were established by analyses of their positive HRESIMS data, in which the sodium adduct ions ([M + Na]^+^) peaks appeared at *m*/*z* 409.2568, *m*/*z* 425.2512, *m*/*z* 425.2512, *m*/*z* 441.2452, and *m*/*z* 405.2239, respectively. Further, the above determination of molecular formulas was supported by analysis of the NMR data (Table 1 and Table 2), indicating three, three, three, three, and five indices of hydrogen deficiency, respectively, for **1**–**4** and **6**. The IR spectra of sporulositols **A**–**D** (**1**–**4**) and *seco*-sporulositol (**6**) showed the presence of hydroxyl (*ν*_max_ 3384.7–3416.6 cm^−1^) and olefinic (*ν*_max_ 1632.0–1659.0 cm^−1^) groups in their structures, and beyond these, there was an aromatic (*ν*_max_ 1597.8, 1554.4, and 1432.7 cm^−1^) group in **6**.

**Figure 2:**
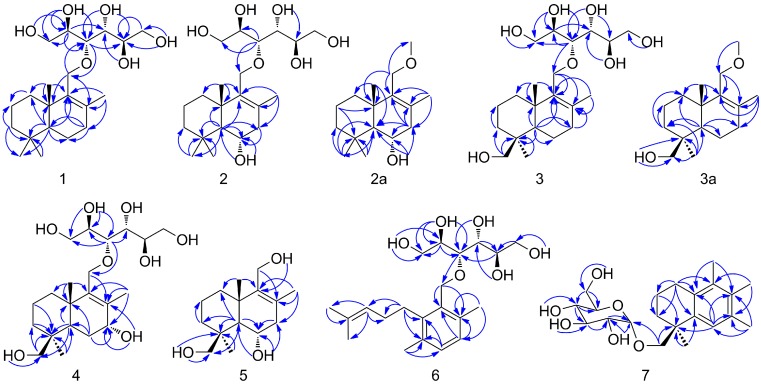
The key HMBC correlations for **1**–**7**.

The NMR spectra (Table 1 and Table 2) of **1**–**4** and **6** all displayed very similar signals for a hexitol moiety (*δ*_H_ 3.51–3.54, 3.40–3.42/*δ*_C_ 62.9–63.0, *δ*_H_ 3.65–3.68/*δ*_C_ 72.6–72.8, *δ*_H_ 3.54–3.57/*δ*_C_ 77.6–77.7, *δ*_H_ 3.50–3.51/*δ*_C_ 71.0–71.1, *δ*_H_ 3.49–3.51/*δ*_C_ 71.3–71.4, and *δ*_H_ 3.60–3.61, 3.35–3.36/*δ*_C_ 63.9–64.0 for **1**–**4**; *δ*_H_ 3.56, 3.43/*δ*_C_ 63.0, *δ*_H_ 3.72/*δ*_C_ 72.3, *δ*_H_ 3.76/*δ*_C_ 78.1, *δ*_H_ 3.58/*δ*_C_ 70.9, *δ*_H_ 3.54/*δ*_C_ 71.3, and *δ*_H_ 3.63, 3.41/*δ*_C_ 63.8 for **6**) and the corresponding free hydroxyl groups (*δ*_H_ 4.20–4.77), indicating that the hexitol moieties existed in **1**–**4** and **6** share the same structures with similar configurations, and the same etherification positions with the remaining skeletons. The deduction was also supported by the HMBC correlations (Figure 2) of **1** from *δ*_H_ 3.68 (H-2′) to *δ*_C_ 63.0 (C-1′), 77.6 (C-3′) and 71.1 (C-4′); from *δ*_H_ 4.57 (HO-1′) to *δ*_C_ 72.8 (C-2′); from *δ*_H_ 4.72 (HO-2′) to *δ*_C_ 63.0, 72.8 and 77.6; from 3.57 (H-3′) to *δ*_C_ 63.0, 72.8 and 71.4 (C-5′); from *δ*_H_ 3.68 (H-2′) to *δ*_C_ 63.0, 77.6 and 71.4 (C-5′); from *δ*_H_ 4.20 (HO-4′) to *δ*_C_ 77.6, 71.1 and 71.4; from *δ*_H_ 3.61, 3.35 (H_2_-6′) to *δ*_C_ 71.4 and from *δ*_H_ 4.32 (HO-6′) to *δ*_C_ 71.4 and 64.0 (C-6′).

The remaining ^13^C-NMR data (Table 1) of **1** included fifteen carbon signals, attributable to four methyls (*δ*_C_ 19.8, 20.7, 21.5, 33.1), six methylenes (*δ*_C_ 18.6, 18.7, 33.3, 36.1, 41.4, 66.8), one methine (*δ*_C_ 51.1) and four quaternary carbons (*δ*_C_ 33.2, 37.5, 131.7, 138.4), supported by analyses of the ^1^H-NMR and HSQC data. Among these carbons, there were one oxygenated methylene {*δ*_H_ 4.00 (d, *J* = 15.0 Hz), 4.02 (d, *J* = 15.0 Hz)/*δ*_C_ 66.8} and one methyl {*δ*_H_ 1.65 (s)/*δ*_C_ 19.8} located at a pair of olefinic carbons (*δ*_C_ 131.7 and 138.4). The 11-hydroxyldrimane-8-en moiety in **1** was then established by HMBC correlations (Figure 2) from *δ*_H_ 4.00, 4.02 (H_2_-11) to *δ*_C_ 131.7 (C-8), *δ*_C_ 138.4 (C-9) and *δ*_C_ 37.5 (C-10), from *δ*_H_ 1.65 (H_3_-12) to *δ*_C_ 33.3 (C-7), C-8 and C-9, from *δ*_H_ 0.92 (H_3_-15) to *δ*_C_ 36.1 (C-1), C-9 and C-10, from *δ*_H_ 1.05 (H-5) to C-1, C-9, C-10, *δ*_C_ 33.2 (C-4), *δ*_C_ 21.5 (C-13) and *δ*_C_ 18.6 (C-6), from *δ*_H_ 2.00, 2.01 (H_2_-7) to C-6, C-8, C-9 and *δ*_C_ 51.1 (C-5), from *δ*_H_ 1.89, 1.18 (H_2_-1) to C-10 and *δ*_C_ 18.7 (C-2), from *δ*_H_ 1.36, 1.11 (H_2_-3) to C-2, C-4 and *δ*_C_ 21.5 (C-13), and from *δ*_H_ 0.81 (H_3_-13), 0.86 (H_3_-14) to C-4. Then, the HMBC correlations from *δ*_H_ 3.57 (H-3′) to *δ*_C_ 66.8 (C-11) and from *δ*_H_ 4.00, 4.02 (H_2_-11) to *δ*_C_ 77.6 (C-3′) established the above deduced hexitol and drimane-type sesquiterpenoid moieties to afford the planar structure of **1** through C_11_-O-C_3_.

The NMR data (Table 1) of **2** at *δ*_H_/*δ*_C_ 1.28, 1.10/43.5 (CH_2_-3), 1.09/55.7 (CH-5), 3.88/66.1 (-O-CH-6), 2.29, 1.98/45.0 (CH_2_-7), 0.99/22.0 (CH_3_-13), 1.12/36.5 (CH_3_-14), 0.94/22.0 (CH_3_-15) and *δ*_C_ 130.4 (C-8), 40.0 (C-10), were quite different from those of **1**, indicating that C-6 was hydroxylated in **2** due to the significant downfield shift of *δ*_C_ 66.1. The assignment was further confirmed by the key HMBC correlations (Figure 2) from *δ*_H_ 4.22 (HO-6) to *δ*_C_ 55.7 (C-5) and 66.1 (C-6), from both *δ*_H_ 1.09 (H-5) and *δ*_H_ 2.29, 1.98 (H-7) to C-6. Finally, the HMBC correlation from *δ*_H_ 3.54 (H-3′) to *δ*_C_ 66.7 (C-11) indicated that C-11 of the 6,11-dihydroxyldrimane-8-en moiety and C-3’ of the hexitol formed the planar structure of **2** by an *O*-ether bridge.

Comparison of NMR data (Table 1) of **3** with those of **1** and **2** revealed that CH_3_-13 of **3** was hydroxylated instead of CH_2_-6 in **2**, in clue of the differences at *δ*_H_/*δ*_C_ 1.77, 0.78/35.1 (CH_2_-3), 1.14/51.9 (CH-5), 3.52, 3.14/62.6 (-O-CH_2_-13), 0.87/27.3 (CH_3_-14), 0.89/21.3 (CH_3_-15) and *δ*_C_ 38.4 (C-4). The deduction was also supported by the key HMBC correlations (Figure **2**) from *δ*_H_ 4.14 (HO-13) to *δ*_C_ 38.4 (C-4) and from *δ*_H_ 3.52, 3.14 (H_2_-13) to *δ*_C_ 35.1 (C-3) and C-4. Further, the HMBC correlation of *δ*_H_ 3.55 (H-3′)/*δ*_C_ 66.8 (C-11) suggested that the 11,13-dihydroxyldrimane-8-en (viz. diaporol [35]) and hexitol moieties in **3** formed the planar structure in the same way as compounds **1** and **2**.

Different from NMR data (Table 1) of **3**, the signals in the NMR data of **4** at *δ*_H_/*δ*_C_ 1.49/45.4 (CH-5), 1.68, 1.57/28.8 (CH_2_-6), 3.69/68.2 (-O-CH-7), 1.74/17.6 (CH_3_-12), 0.85/19.5 (CH_3_-15) and *δ*_C_ 133.5 (C-8), 140.6 (C-9), 38.2 (C-10) suggested that CH_2_-7 was hydroxylated in **4**, which was also supported by the key HMBC correlations from *δ*_H_ 4.56 (HO-7) to *δ*_C_ 68.2 (C-7) and C-8, from *δ*_H_ 3.69 (H-7) to C-8, and from both *δ*_H_ 1.74 (H_3_-12) and *δ*_H_ 1.68, 1.57 (H_2_-6) to C-7. The planar structure of **4** was finally established by HMBC correlation (Figure 2) from *δ*_H_ 3.57 (H-3′) to *δ*_C_ 66.8 (C-11), combining the 7,11,13-trihydroxyldrimane-8-en and hexitol moieties in **4** at the same positions as compounds **1**–**3**.

6-Hydroxydiaporol (**5**) was obtained as a colorless oil. Its molecular formula of C_15_H_26_O_3_ was determined by analyses of its NMR data (Table 1) and positive HRESIMS (*m*/*z* 277.1814 [M + Na]^+^, calcd for C_15_H_26_O_3_, 254.1882) data. Based on HSQC correlations, NMR spectra of **5** showed the presence of three methyls (*δ*_H_ 1.61/*δ*_C_19.2, *δ*_H_ 1.13/*δ*_C_ 31.4, and *δ*_H_ 0.98/*δ*_C_ 22.9), six methylenes (*δ*_H_ 1.78, 1.29/*δ*_C_ 37.0, *δ*_H_ 1.45, 1.33/*δ*_C_ 18.6, *δ*_H_ 1.58, 0.90/*δ*_C_ 38.1, *δ*_H_ 2.20, 1.94/*δ*_C_ 44.5, *δ*_H_ 3.91, 3.81/*δ*_C_ 56.5, and *δ*_H_ 3.78, 3.37/*δ*_C_ 65.1), two methines (*δ*_H_ 1.21/*δ*_C_ 56.5 and *δ*_H_ 3.96/*δ*_C_ 66.9) and four quaternary carbons (*δ*_C_ 38.7, 40.4, 128.5, 140.8). Among the deduced groups, there were two oxygenated methylenes {*δ*_H_ 3.91 (d, *J* = 11.6, 4.4 Hz), 3.81 (d, *J* = 11.6, 4.8 Hz)/*δ*_C_ 56.5 and *δ*_H_ 3.78 (d, *J* = 10.4, 4.8 Hz), 3.37 (d, *J* = 10.4, 4.4 Hz)/*δ*_C_ 65.1} and one oxygenated methine {*δ*_H_ 3.96 (m)/*δ*_C_ 66.9}. Then, the planar structure of **5** was constructed by the key HMBC correlations (Figure 2) from *δ*_H_ 1.61 (H_3_-12) to *δ*_C_ 44.5 (C-7), 128.5 (C-8) and 140.8 (C-9), from *δ*_H_ 3.91, 3.81 (H_2_-11) to C-8, C-9 and *δ*_C_ 40.4 (C-10), from *δ*_H_ 4.11 (HO-11) to *δ*_C_ 140.8, from *δ*_H_ 0.98 (H_3_-13) to *δ*_C_ 140.8, 40.4 and 37.0 (C-1), from *δ*_H_ 1.21 (H-5) to *δ*_C_ 37.0, 40.4, 38.1 (C-3), 38.7 (C-4), and 66.9 (C-6), from *δ*_H_ 4.43 (HO-6) to *δ*_C_ 56.5 (C-5), 66.9 and 44.5, from *δ*_H_ 2.20, 1.94 (H_2_-7) to *δ*_C_ 66.9 and 128.5, from *δ*_H_ 4.78 (HO-13) to *δ*_C_ 65.1 (C-13) and 38.7, from *δ*_H_ 1.13 (H_3_-14) to *δ*_C_ 38.7, and from *δ*_H_ 1.58, 0.91 (H_2_-3) to *δ*_C_ 38.7, 18.6 (C-2). Thus, **5** tuned out to be 6,11,13-trihydroxyldrimane-8-en, similar to the sesquiterpenoid cores in **1**–**4**.

**Figure 3:**
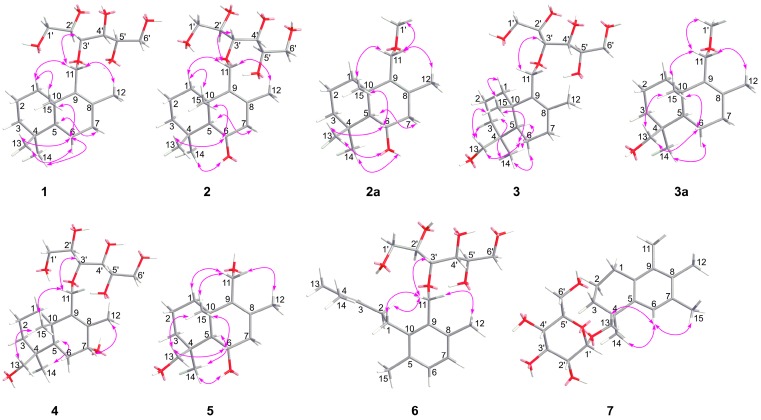
Selected NOE correlations for **1**–**7**.

**Figure 4:**
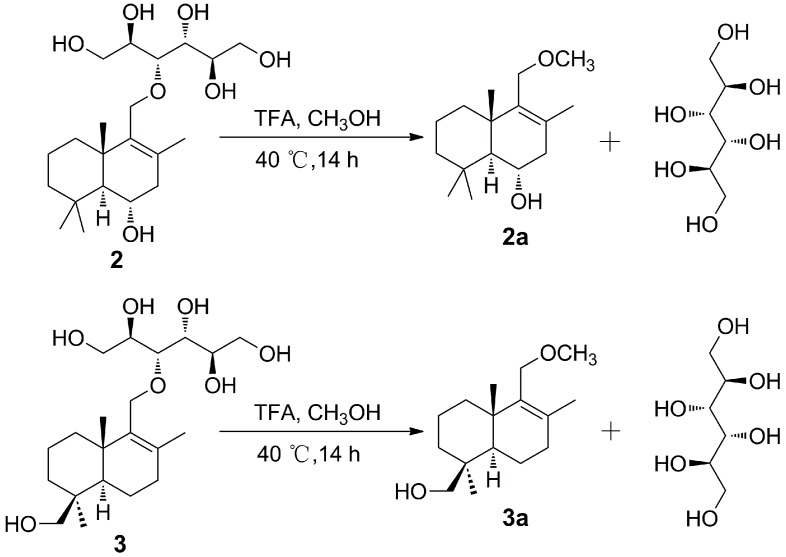
Acid hydrolysis of **2** and **3**.

The relative configurations of compounds **1**–**5** were assigned by ^1^H-NMR *J*-values and NOE correlations (Figure 3). In these drimane-type sesquiterpenoids, the doublet *J* values (11.1–13.2 Hz) of H-5 suggested a *trans*-junction of the two cyclohexatomic ring system [35], H_3_-15 and H_3_-13 (or H_2_-13) adopt the same *β*-orientation, whereas H_3_-14, H-5 and HO-6 (or HO-7) oriented in the opposite *α*-direction. To determine the absolute configurations of **1**–**5**, compounds **2** and **3** were selected and subjected to acid hydrolysis (5% trifluoroacetic acid in methanol; Figure 4), due to their abundant amounts.

**Figure 5:**
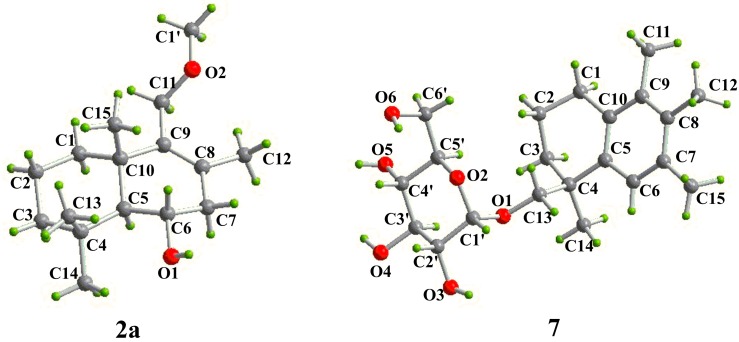
Diamond plot for X-ray crystal structures of **2a** and **7**.

**Figure 6:**
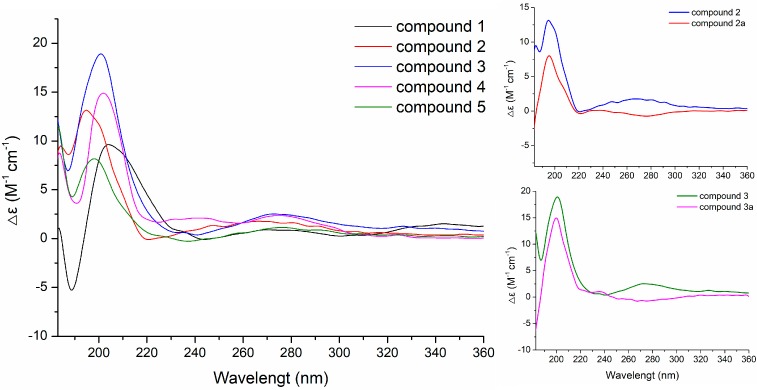
Experimental CD spectra of **1**–**5** in CH_3_CN.

Acid hydrolysis of **2** and **3** afforded a hexitol, together with **2a** and **3a**, respectively, whose structures were elucidated by extensive NMR spectroscopic data (Figure 2, Figure 3 and Figures S19–S29, S38–S45 in supplementary materials). Furthermore, NMR (Figure S28 and S29) and optical rotation data (${\left[\alpha \right]}_{D}^{25}$ +135.5 (*c* 0.380, CH_3_OH)) of the hexitol were quite similar as those reported of D-mannitol [44], which was also isolated from the same strain *P. sporulosum* YK-03. NOESY spectra of **2a** and **3a** (Figure 3) indicated that they share the same relative configurations as compounds **1**–**5**. After many attempts, crystal of **2a** suitable for single-crystal X-ray diffraction (Cu K*α*) analysis (Figure 5) was successfully obtained upon slow evaporation of the solvent mixture (methanol-water, 20:1) by keeping the sample at room temperature for nearly one month. Thus, the absolute configuration of **2a** was unambiguously determined as 5*S*,6*S*,10*S*. Based on the fact that the CD patterns of **1**–**5** and **3a** (Figure 6) were identical to that of **2a**, the absolute configurations of drimane-type sesquiterpenoid were assigned as 5*S*,10*S* in **1**, 5*S*,6*S*,10*S* in **2**, 4*S*,5*R*,10*S* in **3a**, 4*S*,5*R*,10*S* in **3**, 4*S*,5*R*,7*R*,10*S* in **4**, and 4*S*,5*R*,6*S*,10*S* in **5**.

Besides the NMR data of the hexitol, the remaining NMR signals of **6** (Table 2) showed the existence of a prenyl methyl {*δ*_H_/*δ*_C_ 1.55 (s)/17.5, 1.66 (s)/25.6, 5.25 (t, *J* = 6.0 Hz)/124.3, 2.05 (m)/29.5, 2.71 (t, *J* = 8.4 Hz) /29.2 and *δ*_C_ 131.0} and a tetrasubstituted benzene {*δ*_H_/*δ*_C_ 6.90 (d, *J* = 7.8 Hz)/127.6, 6.98 (d, *J* = 7.8 Hz)/129.7 and *δ*_C_ 133.2, 134.9, 135.6, 140.2} groups, assisted by HMBC correlations (Figure 2). 

Then, the HMBC correlations (Figure **2**) from *δ*_H_ 2.25 (s) to *δ*_C_ 129.7 (C-7) and 140.2 (C-8), and from *δ*_H_ 2.34 (s) to *δ*_C_ 127.6 (C-6) and 134.9 (C-5), from *δ*_H_ 4.67 (d, *J* = 10.2 Hz), 4.60 (d, *J* = 10.2 Hz) to *δ*_C_ 133.2 (C-10), 135.6 (C-9) and 140.2, and from *δ*_H_ 2.71 to *δ*_C_ 133.2 and 134.9 led to the assignment of the tetrasubstituted benzene with two methyls located at C-5 and C-8, an oxygenated methyl located at C-9, and a prenyl methyl located at C-10. Further, the HMBC correlation of *δ*_H_ 3.76 (m, H-3′) to *δ*_C_ 67.4 (C-11) indicated C-3’ was connected to C-11 by an ether O to afford the structure of **6**. Based on the NMR data and biosynthetic homology, the hexitol moiety of **6** was presumed to be D-mannitol, the same as that of **1**–**5**.

Sporuloside (**7**) was obtained as colorless oil. The molecular formular of C_21_H_32_O_6_ was established by its positive HRESIMS data at *m*/*z* 403.2103 [M + Na]^+^. Analysis of the NMR data of **7** (Table 2) indicated the existence of an *α*-glucosyl {*δ*_H_/*δ*_C_ 4.64 (d, *J* = 3.6 Hz < 7.0 Hz)/99.3, 3.20 (m)/72.6, 3.40 (m)/73.7, 3.05 (td, *J* = 9.1, 5.2 Hz)/70.7, 3.28 (m)/73.3, and 3.56 (m), 3.40 (m)/61.4} moiety, a pentasubstituted benzene ring {*δ*_H_/*δ*_C_ 7.01 (s)/126.1 and *δ*_C_ 132.1, 132.7, 133.0, 134.1, 139.2}, and three adjacent aromatic methyls {*δ*_H_/*δ*_C_ 2.07 (s)/15.8, 2.09 (s)/15.9, 2.18(s)/21.0}, supported by the key HMBC correlations (Figure 2) from *δ*_H_ 2.18 (H_3_-15) to *δ*_C_ 126.1 (C-6), 133.0 (C-7), 132.1 (C-8), from *δ*_H_ 2.09 (H_3_-12) to *δ*_C_ 133.0, 132.1, 134.1 (C-9), from *δ*_H_ 2.07 (H_3_-11) to *δ*_C_ 132.1, 134.1, 132.7 (C-10), and from *δ*_H_ 7.01(H-6) to *δ*_C_ 133.0, 132.1, 132.7. The rest of NMR signals attributed to four methylenes {*δ*_H_/*δ*_C_ 2.53 (t, *J* = 8.4 Hz)/28.2, 1.72 (m)/19.2, 1.84, 1.44 (each m)/33.0, 3.59, 3.24 (each d, *J* = 9.2 Hz)/75.6}, a methyl {*δ*_H_/*δ*_C_ 1.25 (s)/27.1} and one quaternary carbon {*δ*_C_ 37.8}. The HMBC correlations of *δ*_H_ 2.53 (H_2_-1)/*δ*_C_ 19.2 (C-2), 33.0 (C-3), 139.2 (C-5), 134.1, 132.7, *δ*_H_ 1.72 (H_2_-2)/*δ*_C_ 33.0, 37.8 (C-4), *δ*_H_ 1.25 (H_3_-14)/*δ*_C_ 37.8, 139.2, *δ*_H_ 7.01/*δ*_C_ 37.8, and *δ*_H_ 3.59, 3.24 (H_2_-13)/*δ*_C_ 37.8, 99.3 (C-1′) combined the abovementioned groups to afford the planar structure of **7**, in which C-13 was glycosidated by a *α*-glucose. Luckily, crystals of **7** suitable for single-crystal X-ray diffraction (Cu K*α*) analysis (Figure 5) were successfully obtained upon slow evaporation of the solvent mixture (methanol-water, 20:1) by keeping the sample at room temperature for nearly one month, so the absolute configuration of C-4 was assigned as *S*, and *α*-glucosyl group as D-form.

### 2.2. Investigation on the Origin of Mannitol Moiety

**Figure 7:**
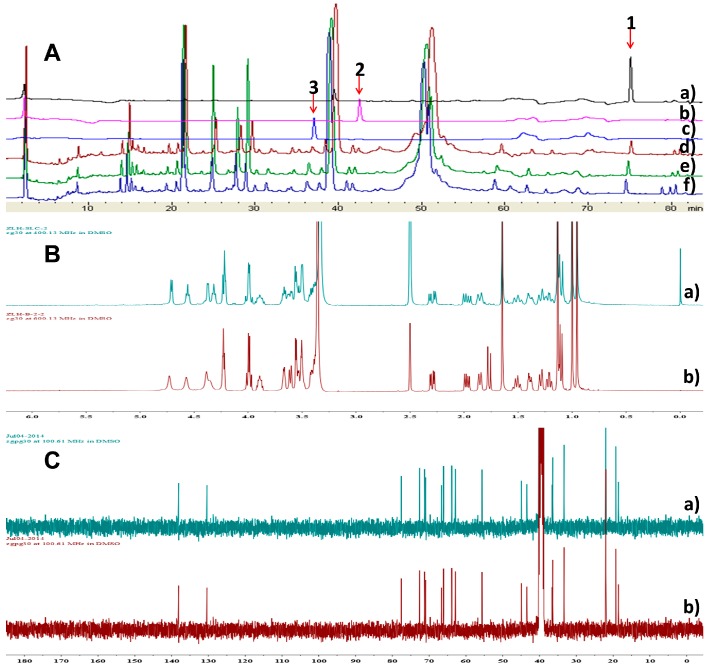
Analysis of the reference compounds **1**–**3** and metabolites of *P. sporulosum* YK-03 in different mediums using HPLC and NMR methods. (**A**) HPLC Analysis of the reference compounds **1**–**3** and metabolites of *P. sporulosum* YK-03 in different mediums: (a) compound **1**; (b) compound **2**; (c) compound **3**; (d) metabolites from blank group (medium with-No mannitol); (e) metabolites from control group (Normal medium, mannitol-contained medium); (f) metabolites from experimental group (medium with mannitol replaced by sorbitol); ^1^H-NMR (**B**) and ^13^C-NMR (**C**) spectra of compound **2** isolated from the experimental and control groups: (a) experimental group; (b) control group.

Sporulositols **A**–**D** (**1**–**4**) and *seco*-sporulositol (**6**) represent the first examples of unique drimanic mannitol derivatives. To find out whether their mannitol moiety was formed intrinsically or derived from the medium, the normal medium (mannitol-contained, control group), modified medium No.1 (no mannitol, blank group) and modified medium No.2 (mannitol replaced by sorbitol, experimental group) were included for simultaneous cultivation of *P. sporulosum* YK-03 and HPLC analysis of the metabolites. Compounds **1**–**3** could be detected in all the three groups (Figure 7A), and compound **2** was isolated from the extract of experimental group (Figure 7B,C), revealing that the fungus can produce the mannitol moiety intrinsically, no matter whether the medium contains mannitol or not.

Compounds **1**–**7** were tested for cytotoxicity against two cell lines A549 (human lung adenocarcinoma cells) and MCF-7 (human breast cancer cells). Unfortunately, compounds **1**–**7** did not show any detectable cytotoxicity.

## 3. Materials and Methods

### 3.1. General Experimental Procedures

Optical rotations were measured using a Perkin-Elmer Model 241 polarimeter (Perkin Elmer, Inc. Waltham, MA, USA). UV spectra were obtained on a Shimadzu UV-1601 (Shimadzu Corp., Kyoto, Japan). IR spectra were taken on a Bruker IFS-55 infrared spectrophotometer (Bruker Optik BmbH, Ettlingen, Germany) with KBr disks. The HRESIMS data were obtained on a microTOF-Q Bruker mass instrument (Bruker Daltonics, Billerica, MA, USA). CD spectra were recorded with a Biologic MOS-450 spectrometer (BioLogic Science Instruments, Grenoble, French) using CH_3_CN as solvent. 1D and 2D NMR spectra were recorded on Bruker ARX-400 and AV-600 spectrometers (^1^H/^13^C, 400/100 MHz 600/150 MHz, Bruker, Zurich, Switzerland) using TMS as an internal standard. Chemical shifts (δ) were expressed in ppm. HPLC was performed using a Shimadzu LC-20AB HPLC pump equipped with a SPD-20A detector (Shimadzu Corp.) for new compound analysis, employing a YMC-Pack ODS-A column (250 mm × 4.6 mm, 5 µm), and for metabolite analysis in Figure 7, employing a CHIRALPAK AD-H column (250 mm × 4.6 mm, 5 μm). Reversed-phase HPLC was performed using a Shimadzu LC-8A HPLC pump equipped with SPD-10A detector for the purification of new compounds, employing a YMC-Pack ODS-A column (250 mm × 10 mm, 5 µm). Column chromatography (CC) was carried out on silica gel (200–300/400–500 mesh, Qingdao Marine Chemical, Inc., Qingdao, China) and sephadex LH-20 (Pharmacia, Uppsala, Sweden). Column fractions were monitored by TLC (Silica gel GF254, 200–300 mesh, Qingdao Haiyang Chemical Factory, Qingdao, China), and the spots were visualized by heating the plates after spraying with 10% H_2_SO_4_ in ethanol. All reagents of HPLC or analytical grade were purchased from Shangdong Yuwang Reagent Co., Ltd. (Shangdong, China).

### 3.2. Fungal Material

*Paraconiothyrium sporulosum* YK-03 was isolated from the sea mud collected from the intertidal zone of Bohai Bay in Liaoning Province of China. It was identified based on the analysis of ITS sequence (GenBank accession No. KC416199) and has been deposited in the School of Traditional Chinese Materia Medica, Shenyang Pharmaceutical University.

### 3.3. Fermentation

The strain was cultured on PDA (potato 20%, glucose 2% and agar 2%) medium in Petri dishes at 28 °C for 3 days, and were inoculated in a 500 mL Erlenmeyer flask containing 150 mL of media (maltose 2%, monosodium glutamate 1%, glucose 1%, yeast cream 0.3%, corn steep liquor 0.1%, maltose 2%, KH_2_PO_4_ 0.05%, MgSO_4_^.^7H_2_O 0.03%). After incubation at 28 °C and 180 rpm for 4 days, a 5 mL cultural solution was transferred as a seed into each of 500 mL flask containing 150 mL liquid medium (maltose 2%, monosodium glutamate 1%, glucose 1%, yeast cream 0.5%, east cream 0.3%, corn steep liquor 0.1%, mannitol 2%, KH_2_PO_4_ 0.05%, MgSO_4_^.^7H_2_O 0.03%, CaCO_3_ 2%, sea water element 3.3%, pH6.5). The flasks were subsequently incubated at the same conditions for 8 days.

### 3.4. Extraction and Isolation

Following incubation, the fermentation broth of *P. sporulosum* YK-03 (70 L) was concentrated and extracted with ethyl acetate and *n*-butanol, successively. The ethyl acetate extract (20 g) was subjected to a silica gel column (10 cm × 120 cm), eluted with CHCl_3_-CH_3_OH (100:1–0:1), yielding 14 fractions **A**–**N**. Fraction **L** (350 mg) was firstly subjected to a Sephadex LH-20 column (2.5 cm × 100 cm), eluted with CHCl_3_-CH_3_OH (1:1) to remove pigment, then purified LPLC using a gradient of increasing methanol (20%–100%) in water to afford three subfractions (**L1**–**L3**). Fraction **L1** (48 mg) afforded compound **1** (4.3 mg, *t*_R_ 36.4 min) and compound **6** (2.5 mg, *t*_R_ = 58.9 min) by using preparative HPLC (CH_3_OH-H_2_O 53:47, flow rate 3 mL/min, wavelength 210 nm), employing a YMC-Pack ODS-A column (250 mm × 10 mm, 5 µm). Fraction **M** (628 mg) was subjected to Sephadex LH-20 column (3 cm × 120 cm), eluted with CHCl_3_-CH_3_OH (1:1) and preparative HPLC (CH_3_OH-H_2_O 44:56, flow rate 3 mL/min, wavelength 210 nm) to obtain compound **2** (93.3 mg, *t*_R_ 40.2 min) compound **3** (89.8 mg, *t*_R_ = 45.5 min), and compound **4** (2.6 mg, *t*_R_ = 58.2 min). Fraction **N** (1.2 g) was subjected to a silica gel column (4 cm × 80 cm), eluted with CHCl_3_-CH_3_OH (100:1–0:1), yielding 7 fractions (**N1**–**N7**). Then, fraction **N4** (89 mg) afforded compound **8** (3.2 mg) through recrystallization, and the rest solution was purified by preparative HPLC (CH_3_OH-H_2_O 30:70, flow rate 3 mL/min, wavelength 210 nm) to obtain compound **5** (2.3 mg, *t*_R_ = 42.0 min) and compound **7** (2.0 mg, *t*_R_ = 49.2 min).

Sporulositol A (**1**): colorless oil; ${\left[\alpha \right]}_{D}^{25}$+101.6 (*c* 0.43, CH_3_OH); UV (CH_3_OH λ_max_ 204.4 nm; IR (KBr) *ν*_max_ 3416.6, 2928.1, 1659.0, 1461.7, 1384.4, 1080.7 cm^−1^; CD (*c* 0.10, CH_3_CN) λ(Δε) 203 (+7.75) nm; ^1^H-NMR (600 MHz, DMSO-*d*_6_) and ^13^C NMR (150 MHz, DMSO-*d*_6_), see Table 1; HRESIMS *m*/*z* 409.2568 [M + Na]^+^, (calcd for C_21_H_38_O_6_ 386.2668).

Sporulositol B (**2**): colorless oil; ${\left[\alpha \right]}_{D}^{25}$+113.9 (*c* 1.00, CH_3_OH); UV (CH_3_OH) λ_max_ 206.4 nm; IR (KBr) *ν*_max_ 3396.3, 2927.4, 1632.3 1434.2, 1384.2, 1075.0, 1043.1 cm^−1^; CD (*c* 0.10, CH_3_CN) λ(Δε) 203 (+6.36) nm; ^1^H-NMR (600 MHz, DMSO-*d*_6_) and ^13^C NMR (150 MHz, DMSO-*d*_6_), see Table 1; HRESIMS *m*/*z* 425.2512 [M + Na]^+^, (calcd for C_21_H_38_O_7_ 402.2617).

Sporulositol C (**3**): colorless oil; ${\left[\alpha \right]}_{D}^{25}$+101.8 (*c* 1.00, CH_3_OH); UV (CH_3_OH) λ_max_ 206.2,243.6 nm; IR (KBr) *ν*_max_ 3384.7, 2928.2, 1650.1, 1451.1, 1384.0, 1079.0, 1032.4 cm^−1^; CD (*c* 0.10, CH_3_CN) λ(Δε) 202 (+10.79) nm; ^1^H-NMR (600 MHz, DMSO-*d*_6_) and ^13^C NMR (150 MHz, DMSO-*d*_6_), see Table 1, HRESIMS *m*/*z* 425.2512 [M + Na]^+^, (calcd for C_21_H_38_O_7_ 402.2617).

Sporulositol D (**4**): colorless oil; ${\left[\alpha \right]}_{D}^{25}$+90.3 (*c* 0.26, CH_3_OH); UV (CH_3_OH) λ_max_ 207.2 nm; IR (KBr) *ν*_max_ 3405.7, 2922.7, 1632.3, 1597.6, 1552.5, 1430.0, 1384.4, 1121.3, 1053.1, 1033.0 cm^−1^; CD (*c* 0.10, CH_3_CN) λ(Δε) 201 (+11.07) nm;^1^ H-NMR (600 MHz, DMSO-*d*_6_) and ^13^C NMR (150 MHz, DMSO-*d*_6_), see Table 1; HRESIMS *m*/*z* 441.2452 [M + Na]^+^, (calcd for C_21_H_38_O_8_ 418.2566).

6-hydroxyl diaporol (**5**): colorless oil; ${\left[\alpha \right]}_{D}^{25}$+98.3 (c 0.23, CH_3_OH); UV (CH_3_OH) λ_max_ 207.2 nm; IR (KBr) *ν*_max_ 3418.1, 2924.9, 1658.1, 1554.1, 1433.8, 1384.3 cm^−1^; CD (*c* 0.10, CH_3_CN) λ(Δε) 196.5 (+6.12) nm; ^1^H-NMR (600 MHz, DMSO-*d*_6_) and ^13^C NMR (150 MHz, DMSO-*d*_6_), see Table 1; HRESIMS *m*/*z* 277.1814 [M + Na]^+^, (calcd for C_15_H_26_O_3_ 254.1882).

*seco*-sporulositol (**6**): colorless oil; ${\left[\alpha \right]}_{D}^{25}$+55.0 (*c* 0.25, CH_3_OH); UV (CH_3_OH) λ_max_ 205.0 nm; IR (KBr) *ν*_max_ 3405.4, 2923.0, 1632.0, 1597.8, 1554.4, 1432.7, 1384.4, 1127.7, 1033.1 cm^−1^; ^1^H-NMR (600 MHz, DMSO-*d*_6_) and ^13^C NMR (150 MHz, DMSO-*d*_6_), see Table 2; HRESIMS *m*/*z* 405.2239 [M + Na]^+^, (calcd for C_21_H_34_O_6_ 382.2355).

Sporuloside (**7**): colorless oil; ${\left[\alpha \right]}_{D}^{25}$+117.6 (*c* 0.26, CH_3_OH); UV (CH_3_OH) λ_max_ 205.0 nm; IR (KBr) *ν*_max_ 3405.4, 2921.6, 1634.3, 1457.7, 1384.4, 1148.2, 1123.5, 1025.5 cm^−1^; ^1^H-NMR (600 MHz, DMSO-*d*_6_) and ^13^C NMR (150 MHz, DMSO-*d*_6_), see Table 2; HRESIMS *m*/*z* 403.2103 [M + Na]^+^, (calcd for C_21_H_32_O_6_ 380.2199).

D-mannitol (**8**): white powder; ${\left[\alpha \right]}_{D}^{25}$+145.5 (*c* 0.50, CH_3_OH); ^1^H-NMR (DMSO-*d*_6_, 400 MHz), *δ*_H_ 3.60 (2H, m), 3.54 (2H, t, *J* = 8.0 Hz), 3.46 (2H, m), 3.38 (2H, m), 4.41 (2H, d, *J* = 5.6 Hz, HO ×2 ), 4.33(2H, t, *J* = 6.0 Hz, HO×2), 4.14 (2H, d, *J* = 7.2 Hz, HO × 2); ^13^C-NMR (DMSO-*d*_6_, 100 MHz) *δ*_C_ 64.2 × 2, 70.1 × 2, 71.7 × 2.

### 3.5. X-ray Crystallographic Analysis of Compounds ***2a*** and ***7***

Crystal Data of **2a**: C_16_H_28_O_2_, *M* =252.38, orthorhombic, *a* = 10.0481 (3) Å, *b* = 10.4089(4) Å, *c* = 28.5282(9) Å, *U* = 2983.76(17) Å^3^, *T* = 100.6, space group P2_1_2_1_2_1_ (no. 19), *Z* = 8, μ(Cu Kα) = 0.554, 10773 reflections measured, 5657 unique (*R*_int_ = 0.0286) which were used in all calculations. The final *wR* (*F*_2_) was 0.1314 (all data). The crystallographic data for the structure of **2a** have been deposited with the Cambridge Crystallographic Data Centre as supplementary publication CCDC No. 1905155.

Crystal Data of **7**: C_21_H_32_O_6_, *M* = 380.47, orthorhombic, *a* = 5.39286(9) Å, *b* = 7.54344 (11) Å, *c* = 48.7570(7) Å, *U* = 1983.47(5) Å^3^, *T* = 102.3, space group P2_1_2_1_2_1_ (no. 19), *Z* = 4, μ (Cu Kα) = 0.753, 6585 reflections measured, 3754 unique (*R*_int_ = 0.0252) which were used in all calculations. The final*wR* (*F*_2_) was 0.0996 (all data). The crystallographic data for the structure of **7** have been deposited with the Cambridge Crystallographic Data Centre as supplementary publication CCDC No. 1905156.

CCDC-1905155 and CCDC-1905156 contain the supplementary crystallographic data, which can be obtained free of charge from the Cambridge Crystallographic Data Centre via http://www.ccdc.cam.ac.uk/conts/retrieving.html.

### 3.6. Acid Hydrolysis of Compounds ***2*** and ***3***

Compounds **2** and **3** (each 10 mg) were dissolved in CH_3_OH (1 mL) with 1 mL trifluoroacetic acid (TFA), and heated in a H_2_O bath at 40 °C for 14 h to give an acid hydrolysate. The acid hydrolysate was then vacuum evaporated to remove the residual TFA. Then, the hydrolysate was suspended in H_2_O, and extracted with CHCl_3_. Finally, the H_2_O solution afforded sugar alcohol through recrystallization, and the CHCl_3_ solution was purified by preparative HPLC to obtain compound **2a** (1.8 mg) and compound **3a** (2.0 mg), respectively.

### 3.7. Analysis of the Refrence Compounds ***1***–***3*** and Metabolites of P. Sporulosum YK-03 in Different Mediums Using HPLC and NMR Methods

*P. sporulosum* YK-03 was simultaneously cultured in three following liquid media at 28 °C and 180 rpm for 4 days. Liquid media: (1) Normal medium (mannitol-containing, control group): maltose 2%, monosodium glutamate 1%, glucose 1%, yeast cream 0.5%, east cream 0.3%, corn steep liquor 0.1%, mannitol 2%, KH_2_PO_4_ 0.05%, MgSO_4_^.^7H_2_O 0.03%, CaCO_3_ 2%, sea water element 3.3%, pH 6.5. (2) Modified medium No.1 (no mannitol, blank group): maltose 2%, monosodium glutamate 1%, glucose 1%, yeast cream 0.5%, east cream 0.3%, corn steep liquor 0.1%, KH_2_PO_4_ 0.05%, MgSO_4_^.^7H_2_O 0.03%, CaCO_3_ 2%, sea water element 3.3%, pH 6.5. (3) Modified medium No. 2 (mannitol replaced by sorbitol, experimental group): maltose 2%, monosodium glutamate 1%, glucose 1%, yeast cream 0.5%, east cream 0.3%, corn steep liquor 0.1%, sorbitol 2%, KH_2_PO_4_ 0.05%, MgSO_4_^.^7H_2_O 0.03%, CaCO_3_ 2%, sea water element 3.3%, pH 6.5.

After incubation, their fermentation broths were concentrated and extracted with ethyl acetate. Then their metabolites were analyzed by gradient HPLC analysis. HPLC chromatographic condition: (1) Instrument: Shimadzu LC-20AB HPLC pump equipped with an SPD-20A detector (Shimadzu Corp.); (2) Column: CHIRALPAK AD-H column (250 mm × 4.6 mm, 5 *μ*m); (3) Mobile phase: CH_3_OH (B) and H_2_O (A); (4) Wavelength: 210 nm; (3) Flow rate: 1 mL min^−1^; (5) gradient elution program: 10% B; 0–6 min, linearly changed to 24% B; 6–26 min, linearly changed to 35% B; 26–40 min, linearly changed to 60% B; 40–60 min, linearly changed to 70% B; 60–72 min, linearly changed to 80% B; 72–82 min, linearly changed to 100%. The sample injection volume was 5.0 μL.

The strain was incubated in twenty Erlenmeyer flasks (500 mL) containing 150 mL Modified medium No. 2 medium at 28 °C and 180 rpm for 8 days. Then the fermentation broth was concentrated and extracted with ethyl acetate. The ethyl acetate extract was subjected to a silica gel column eluted with CHCl_3_-CH_3_OH (100:1–0:1) and preparative HPLC (CH_3_OH-H_2_O 44:56, flow rate 3 mL/min, wavelength 210 nm) to yield a drimane-type sesquiterpenoid, guiding by TLC and HPLC. Then its NMR spectra was compared with those of compounds **1**–**3**.

### 3.8. Cytotoxic Activity Assay

The cytotoxicity was evaluated by using the MTT assay as described previously [45]. Doxorubicin hydrochloride was used as a positive control. The A549 and MCF-7 Cells (China Infrastructure of Cell Lines Resources, Beijing, China were cultured in McCoy’s 5A medium and DMEM basic medium (1×) at 37 °C under an atmosphere of 5% CO_2_, and were seeded on each well of 96-well plates containing 200 *μ*L of tumor cell suspension (1 × 10^4^ cells). After 24 h, each well was added 2 μL of test solution and incubated for another 72 h. 50 μL of MTT solution (1 mg/mL, Beijing Cellchip Biotechnology Co., Ltd., Beijing, China was added to each well, and the plate was incubated for 3h under the same condition. Then, the plate was centrifuged and the supernatants were removed and cells were dissolved in 150 μL of DMSO to determine the IC_50_ values.

## 4. Conclusions

Seven new drimane-type sesquiterpenoids, including sporulositols **A**–**D** (**1**–**4**), 6-hydroxy- diaporol (**5**), *seco*-sporulositol (**6**) and sporuloside (**7**), were isolated from a marine-derived fungus *P. sporulosum* YK-03. Their structures were established by extensive NMR experiments, X-ray diffraction analysis and comparisons of circular dichroism data. Compounds **1**–**4** and **6** are rare new drimanic hexitol derivatives containing a D-mannitol moiety. *seco*-sporulositol (**6**) and sporuloside (**7**) may represent two new series of natural drimanes, possessing an aromatic ring with a rare 4,5-secodrimanic skeleton and an unusual CH_3_-15 rearranged drimanic α-D-glucopyranside, respectively. Then, the cultivation medium was evaluated for the origin of D-mannitol moiety by HPLC and NMR analyses. These isolates enriched the structural diversity of natural drimanic sesquiterpenoids.

## Figures and Tables

**Table 1:** NMR spectroscopic data of **1**–**5** ^a^.

No.	1		2		2a ^b^		3		3a ^b^		4		5	
	*δ*_C_	*δ*_H_, mult (*J*, Hz)	*δ*_C_	*δ*_H_, mult (*J*, Hz)	*δ*_C_	*δ*_H_, mult (*J*, Hz)	*δ*_C_	*δ*_H_, mult (*J*, Hz)	*δ*_C_	*δ*_H_, mult (*J*, Hz)	*δ*_C_	*δ*_H_, mult (*J*, Hz)	*δ*_C_	*δ*_H_, mult (*J*, Hz)
1	36.1	1.89, d (13.2); 1.18, td (13.2, 2.4)	36.7	1.84, d (13.2); 1.20, td (13.2, 3.0)	36.9	1.71, d (12.6); 1.18, td (13.2, 3.6)	36.2	1.89, d (13.2); 1.19, dd (10.8, 3.6)	36.4	1.74, d (13.2); 1.16, d (13.2)	35.8	1.88, d (13.2); 1.17, td (13.2, 3.6)	37.0	1.78, m; 1.29, m
2	18.7	1.55, m; 1.43, m	18.6	1.51, qt (13.6, 3.8); 1.37, dt (13.6, 3.5)	18.9	1.50, qt (13.2, 3.6); 1.38, dt (13.2, 3.6)	18.5	1.49, m; 1.38, m	18.6	1.50, m; 1.37, m	18.5	1.55, m; 1.39, m	18.6	1.45, m; 1.33, m
3	41.4	1.36, m; 1.11, td (12.6, 3.6)	43.5	1.28, d (13.2); 1.10, d (10.8)	43.9	1.29, d (13.2); 1.11, m	35.1	1.77, d (13.2); 0.78, td (13.2, 3.6)	35.4	1.77, d (13.2); 0.78, td (13.2, 3.6)	35.2	1.78, d (13.8); 0.82, td (13.2, 3.6)	38.1	1.58, d (13.2); 0.90, td (13.2, 4.0)
4	33.2		33.4		33.8		38.4		38.7		38.0		38.7	
5	51.1	1.05, d (12.6)	55.7	1.09, d (11.1)	56.1	1.08, d (10.8)	51.9	1.14, d (12.6)	52.2	1.13, d (12.6)	45.4	1.49, d (13.2)	56.5	1.21, d (11.2)
6	18.6	1.61, m; 1.37, m	66.1	3.88, m	66.5	3.89, m	18.7	1.67, m; 1.36, m	19.0	1.68, m; 1.37, m	28.8	1.68, d (12.6); 1.57, dd (12.6, 4.8)	66.9	3.96, m
7	33.3	2.00, m; 2.01, m	45.0	2.29, dd (17.8, 6.3); 1.98, dd (17.8, 8.9)	45.2	2.30, dd (18.0, 6.0); 1.96, dd (18.0, 9.0)	33.8	1.94, m; 1.95, m	33.9	1.95, m	68.2	3.69, m	44.5	2.20, dd (17.2, 6.0); 1.94, dd (17.2, 9.6)
8	131.7		130.4		131.0		131.6		131.9		133.5		128.5	
9	138.4		138.2		137.6		138.4		137.8		140.6		140.8	
10	37.5		40.0		40.5		37.5		37.7		38.2		40.4	
11	66.8	4.02, d (15.0); 4.00, d (15.0)	66.7	3.98, d (16.7); 3.97, d (16.7)	67.8	3.80, d (10.5); 3.69, d (10.5)	66.8	4.00, d (12.6); 3.99, d (12.6)	67.9	3.80, d (10.2); 3.68, d (10.2)	66.8	4.02, d (10.2); 3.99, d (10.2)	56.5	3.91, dd (11.6, 4.4); 3.81, dd (11.6, 4.8)
12	19.8	1.65, s	19.3	1.64, s	19.4	1.59, s	19.7	1.63, s	19.6	1.57, s	17.6	1.74, s	19.2	1.61, s
13	21.5	0.81, s	22.0	0.99, s	22.4	0.94, s	62.6	3.52, m; 3.14, m	62.9	3.52, m; 3.13, m	62.8	3.54, m; 3.13, dd (10.8, 5.4)	65.1	3.78, dd (10.4, 4.8); 3.37, dd (10.4, 4.4)
14	33.1	0.86, s	36.5	1.12, s	36.8	1.13, s	27.3	0.87, s	27.6	0.87, s	27.0	0.86, s	31.4	1.13, s
15	20.7	0.92, s	22.0	0.94, s	22.3	0.94, s	21.3	0.89, s	21.4	0.88, s	19.5	0.85, s	22.9	0.98, s
6-OH				4.22, d (6.6)										4.43, d (4.8)
7-OH												4.56, d (5.4)		4.11, t (4.8)
13-OH								4.14, t (4.8)				4.16, t (5.4)		
1′	63.0	3.54, dd (10.2, 4.8); 3.41, m	63.0	3.52, m; 3.40, m			63.0	3.53, m; 3.40, m			62.9	3.51, m; 3.42, m		
2′	72.8	3.68, m	72.6	3.65, m			72.7	3.66, m			72.8	3.67, m		
3′	77.6	3.57, d (4.8)	77.7	3.54, d (4.8)			77.6	3.55, d (4.8)			77.6	3.57, d (4.8)		
4′	71.1	3.51, m	71.0	3.51, m			71.0	3.50, m			71.1	3.51, m		
5′	71.4	3.51, m	71.3	3.49, m			71.3	3.50, m			71.3	3.51, m		
6′	64.0	3.61, dd (10.2, 4.2); 3.35, m	63.9	3.60, d (10.8); 3.36, m			63.9	3.60, m; 3.36, m			63.9	3.61, dd (9.6, 6.0); 3.36, m		
1’-OH		4.57, t (5.4)		4.57, br s				4.54, t (4.8)				4.61, t (5.4)		
2’-OH		4.72, d (4.8)		4.72, br s				4.70, d (4.8)				4.77, d (5.4)		
4’-OH		4.20, br s		4.23, d (5.4)				4.20, d (4.8)				4.25, br d (4.8)		
5’-OH		4.37, br s		4.38, br s				4.36, br s				4.42, br d (4.8)		
6’-OH		4.32, t (5.4)		4.35, br s				4.30, t (4.8)				4.34, t (5.4)		

^a^ The spectra were recorded at 600 (^1^H) and 100 MHz (^13^C) in DMSO-*d*_6_; Assignments were made by a combination of 1D and 2D NMR experiments. ^b^ **2a** and **3a** was obtained by acid hydrolysis of **2** and **3**, respectively.

**Table 2:** NMR spectroscopic data of **6** and **7**.

No.	6 ^a^		7^ b^	
	*δ*_C_	*δ*_H_, mult (*J*, Hz)	*δ*_C_	*δ*_H_, mult (*J*, Hz)
1	29.2	2.71, t (8.4)	28.2	2.53, t (8.4)
2	29.5	2.05, m	19.2	1.72, m
3	124.3	5.25, t (6.0)	33.0	1.84, m 1.44, m
4	131.0		37.8	
5	134.9		139.2	
6	127.6	6.90, d (7.8)	126.1	7.01, s
7	129.7	6.98, d (7.8)	133.0	
8	140.2		132.1	
9	135.6		134.1	
10	133.2		132.7	
11	67.4	4.67, d (10.2) 4.60, d (10.2)	15.8	2.07, s
12	19.5	2.25, s	15.9	2.09, s
13	25.6	1.66, s	75.6	5.59, d (9.2) 3.24, d (9.2)
14	17.5	1.55, s	27.1	1.25, s
15	19.7	2.34, s	21.0	2.18, s
1′	63.0	3.56, m 3.43, m	99.3	4.64, d (3.6)
2′	72.3	3.72, m	72.6	3.20, m
3′	78.1	3.76, d (5.1)	73.7	3.40, m
4′	70.9	3.58, m	70.7	3.05, td (9.1, 5.2)
5′	71.3	3.54, m	73.3	3.28, m
6′	63.8	3.63, m 3.41, m	61.4	3.56, m 3.40, m
1’-OH		4.59, t (5.4)		
2’-OH		4.75, d (5.4)		4.60, d (6.4)
3’-OH				4.74, d (4.8)
4’-OH		4.30, d (5.4)		4.85, d (5.2)
5’-OH		4.40, d (5.4)		
6’-OH		4.35, t (5.4)		4.38, t (6.0)

^a^ The NMR spectra were recorded at 600 (^1^H) and 100 MHz (^13^C) in DMSO-*d*_6_. ^b^ The NMR spectra were recorded at 400 (^1^H) and 100 MHz (^13^C) in DMSO-*d*_6_. Assignments were made by a combination of 1D and 2D NMR experiments.

## Supplementary Materials

The following are available online, Figures S1–S79: HRESIMS, 1D and 2D NMR, IR, UV and CD spectra of compounds **1**–**8**, **2a** and **3a**; Figures S80–S81: 1D NMR of compound **2** isolated from the modified medium No.2.
