# Supplementary material for: Seven New Drimane-Type Sesquiterpenoids from a Marine-Derived Fungus Paraconiothyrium sporulosum YK-03
Source: Molecules. 2019 May 10;24(9):1817. doi: 10.3390/molecules24091817 (PMC6539489; doi:10.3390/molecules24091817)
Supplement: Supplementary file 1 [file molecules-24-01817-s001.zip › supporting materials-R2/supporting materials-R2.pdf]

Supplementary Materials

Seven new drimane-type sesquiterpenoids from a marine-derived fungus *Paraconiothyrium sporulosum* YK-03

Li-Hua Zhang [2,3], Gang Chen [2], Yi Sun [4], Hai-Feng Wang [2], Jiao Bai [2], Hui-Ming Hua [2], Yue-Hu Pei [1,*]

[1] Department of Medicinal Chemistry and Natural Medicine Chemistry, College of Pharmacy, Harbin Medical University, Harbin 150081, China; peiyueh@vip.163.com (Y.-H.P.)

[2] School of Traditional Chinese Materia Medica, Shenyang Pharmaceutical University, Shenyang 110016, China; zhanglihua200061@163.com (L.-H.Z); chengang1152001@hotmail.com (G.C); wanghaifeng0310@163.com (H.-F.W); baijiao@hotmail.com (J.B); huimhua@163.com (H.-M.H)

[3] State Key Laboratory of Bioactive Substance and Function of Natural Medicines, Institute of Materia Medica, Chinese Academy of Medical Sciences & Peking Union Medical College, Beijing 100050, China

[4] Institute of Chinese Materia Medica, China Academy of Chinese Medical Sciences, Beijing 100700, China; sylotus@hotmail.com (Y.S)

* Correspondence: peiyueh@vip.163.com (Y.-H.P.); Tel.: +86-0451-86685745

The List of Contents

No.	Content	Page
1	Figure **S1**. HR ESI-TOF MS spectrum of compound **1**	5
2	Figure **S2**. IR spectrum of compound **1**	5
3	Figure **S3**. UV spectrum of compound **1**	6
4	Figure **S4**. ^{1}H NMR (600 MHz, DMSO-d_6) spectrum of compound **1**	6
5	Figure **S5**. ^{13}C NMR (100 MHz, DMSO-d_6) spectrum of compound **1**	7
6	Figure **S6**. HSQC (600 MHz, DMSO-d_6) spectrum of compound **1**	7
7	Figure **S7**. HMBC (600 MHz, DMSO-d_6) spectrum of compound **1**	8
8	Figure **S8**. NOESY spectrum (600 MHz, DMSO-d_6) of compound **1**	8
9	Figure **S9**. CD spectrum of compound **1** in CH_3CN	9
10	Figure **S10**. HR ESI-TOF MS spectrum of compound **2**	9
11	Figure **S11**. IR spectrum of compound **2**	10
12	Figure **S12**. UV spectrum of compound **2**	10
13	Figure **S13**. ^{1}H NMR (600 MHz, DMSO-d_6) spectrum of compound **2**	11
14	Figure **S14**. ^{13}C NMR (100 MHz, DMSO-d_6) spectrum of compound **2**	11
15	Figure **S15**. HSQC (600 MHz, DMSO-d_6) spectrum of compound **2**	12
16	Figure **S16**. HMBC (600 MHz, DMSO-d_6) spectrum of compound **2**	12
17	Figure **S17**. NOESY spectrum (600 MHz, DMSO-d_6) of compound **2**	13
18	Figure **S18**. CD spectrum of compound **2** in CH_3CN	13
19	Figure **S19**. HR ESI-TOF MS spectrum of compound **2a**	14
20	Figure **S20**. IR spectrum of compound **2a**	14
21	Figure **S21**. UV spectrum of compound **2a**	15
22	Figure **S22**. ^{1}H NMR (600 MHz, DMSO-d_6) spectrum of compound **2a**	15
23	Figure **S23**. ^{13}C NMR (100 MHz, DMSO-d_6) spectrum of compound **2a**	16
24	Figure **S24**. HSQC (600 MHz, DMSO-d_6) spectrum of compound **2a**	16
25	Figure **S25**. HSBC (600 MHz, DMSO-d_6) spectrum of compound **2a**	17
26	Figure **S26**. NOESY (600 MHz, DMSO-d_6) spectrum of compound **2a**	17
27	Figure **S27**. CD spectrum of compound **2a** in CH_3CN	18

28	Figure **S28**. ^{1}H NMR (600 MHz, DMSO-d_6) spectrum of the hexitol	18
29	Figure **S29**. ^{13}C NMR (100 MHz, DMSO-d_6) spectrum of the hexitol	19
30	Figure **S30**. HR ESI-TOF MS spectrum of compound **3**	19
31	Figure **S31**. IR spectrum of compound **3**	20
32	Figure **S32**. UV spectrum of compound **3**	20
33	Figure **S33**. ^{1}H NMR (600 MHz, DMSO-d_6) spectrum of compound **3**	21
34	Figure **S34**. ^{13}C NMR (100 MHz, DMSO-d_6) spectrum of compound **3**	21
35	Figure **S35**. HSQC (600 MHz, DMSO-d_6) spectrum of compound **3**	22
36	Figure **S36**. HMBC (600 MHz, DMSO-d_6) spectrum of compound **3**	22
37	Figure **S37**. CD spectrum of compound **3** in CH_3CN	23
38	Figure **S38**. HR ESI-TOF MS spectrum of compound **3a**	23
39	Figure **S39**. IR spectrum of compound **3a**	24
40	Figure **S40**. UV spectrum of compound **3a**	24
41	Figure **S41**. ^{1}H NMR (600 MHz, DMSO-d_6) spectrum of compound **3a**	25
42	Figure **S42**. ^{13}C NMR (100 MHz, DMSO-d_6) spectrum of compound **3a**	25
43	Figure **S43**. HSQC (600 MHz, DMSO-d_6) spectrum of compound **3a**	26
44	Figure **S44**. HSBC (600 MHz, DMSO-d_6) spectrum of compound **3a**	26
45	Figure **S45**. CD spectrum of compound **3a** in CH_3CN	27
46	Figure **S46**. HR ESI-TOF MS spectrum of compound **4**	27
47	Figure **S47**. IR spectrum of compound **4**	28
48	Figure **S48**. UV spectrum of compound **4**	28
49	Figure **S49**. ^{1}H NMR (600 MHz, DMSO-d_6) spectrum of compound **4**	29
50	Figure **S50**. ^{13}C NMR (100 MHz, DMSO-d_6) spectrum of compound **4**	29
51	Figure **S51**. HSQC (600 MHz, DMSO-d_6) spectrum of compound **4**	30
52	Figure **S52**. HMBC (600 MHz, DMSO-d_6) spectrum of compound **4**	30
53	Figure **S53**. NOESY spectrum (600 MHz, DMSO-d_6) of compound **4**	31
54	Figure S**54**. CD spectrum of compound **4** in CH_3CN	31
55	Figure **S55**. HR ESI-TOF MS spectrum of compound **5**	32
56	Figure **S56**. IR spectrum of compound **5**	32

57	Figure **S57**. UV spectrum of compound **5**	33
58	Figure **S58**. ^{1}H NMR (600 MHz, DMSO-d_6) spectrum of compound **5**	33
59	Figure **S59**. ^{13}C NMR (100 MHz, DMSO-d_6) spectrum of compound **5**	34
60	Figure **S60**. HSQC (600 MHz, DMSO-d_6) spectrum of compound **5**	34
61	Figure **S61**. HMBC (600 MHz, DMSO-d_6) spectrum of compound **5**	35
62	Figure **S62**. NOESY spectrum (600 MHz, DMSO-d_6) of compound **5**	35
63	Figure **S63**. CD spectrum of compound **5** in CH_3CN	36
64	Figure **S64**. HR ESI-TOF MS spectrum of compound **6**	36
65	Figure **S65**. IR spectrum of compound **6**	37
66	Figure **S66**. UV spectrum of compound **6**	37
67	Figure **S67**. ^{1}H NMR (600 MHz, DMSO-d_6) spectrum of compound **6**	38
68	Figure **S68**. ^{13}C NMR (100 MHz, DMSO-d_6) spectrum of compound **6**	38
69	Figure **S69**. HSQC (600 MHz, DMSO-d_6) spectrum of compound **6**	39
70	Figure **S70.** HMBC (600 MHz, DMSO-d_6) spectrum of compound **6**	39
71	Figure **S71**. NOESY spectrum (600 MHz, DMSO-d_6) of compound **6**	40
72	Figure **S72**. HR ESI-TOF MS spectrum of compound **7**	40
73	Figure **S73**. IR spectrum of compound **7**	41
74	Figure **S74**. UV spectrum of compound **7**	41
75	Figure **S75**. ^{1}H NMR (600 MHz, DMSO-d_6) spectrum of compound **7**	42
76	Figure **S76**. ^{13}C NMR (100 MHz, DMSO-d_6) spectrum of compound **7**	42
77	Figure **S77**. HSQC (600 MHz, DMSO-d_6) spectrum of compound **7**	43
78	Figure **S78**. HMBC (600 MHz, DMSO-d_6) spectrum of compound **7**	43
79	Figure **S79**. NOESY spectrum (600 MHz, DMSO-d_6) of compound **7**	44
80	Figure **S80**. ^{1}H NMR (400 MHz, DMSO-d_6) spectrum of **2** isolated from the modified medium No.2	44
81	Figure **S81.** ^{13}C NMR (100 MHz, DMSO-d_6) spectrum of **2** isolated from the modified medium No.2	45

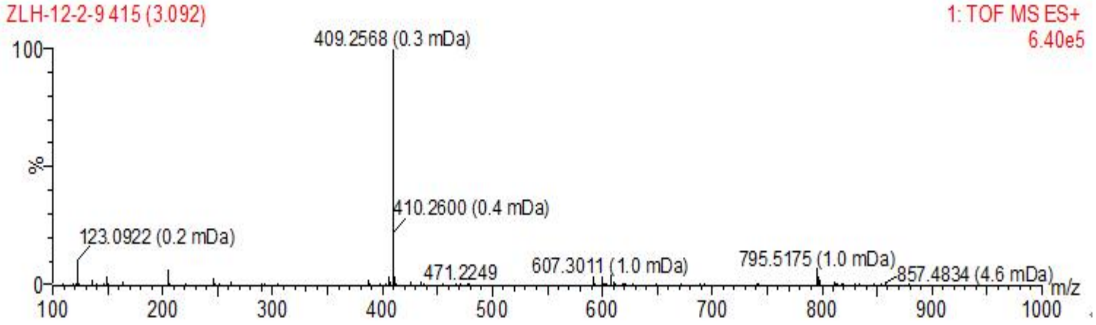

m/z 409.2568 [M + Na] +

Figure S1. HR ESI-TOF MS spectrum of compound 1

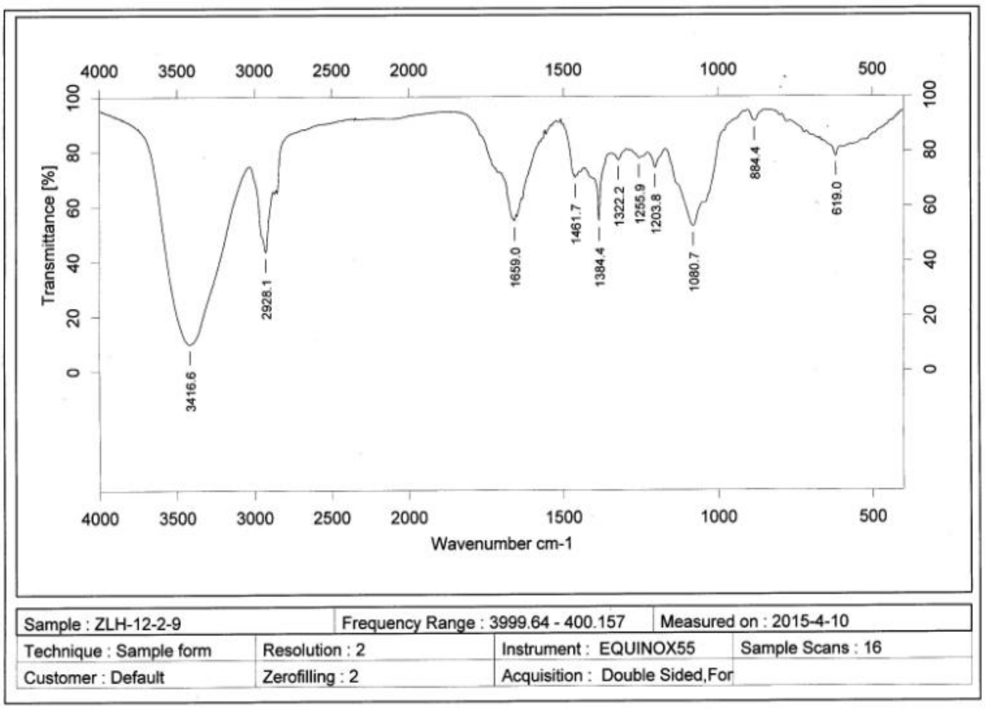

Figure S2. IR spectrum of compound 1

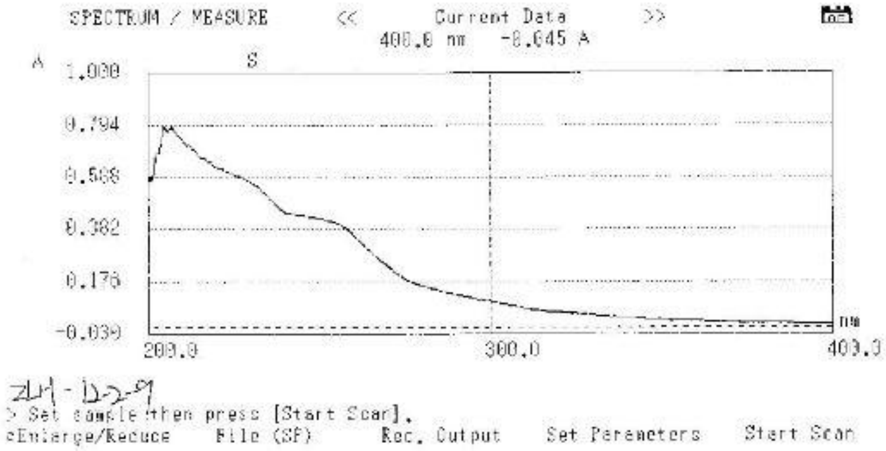

NO.	ABSCISSA	PEAK	HEIGHT	ABSCISSA	VALLEY	HEIGHT
1	204.4	0.7986	0.2396			

Figure S3. UV spectrum of compound 1

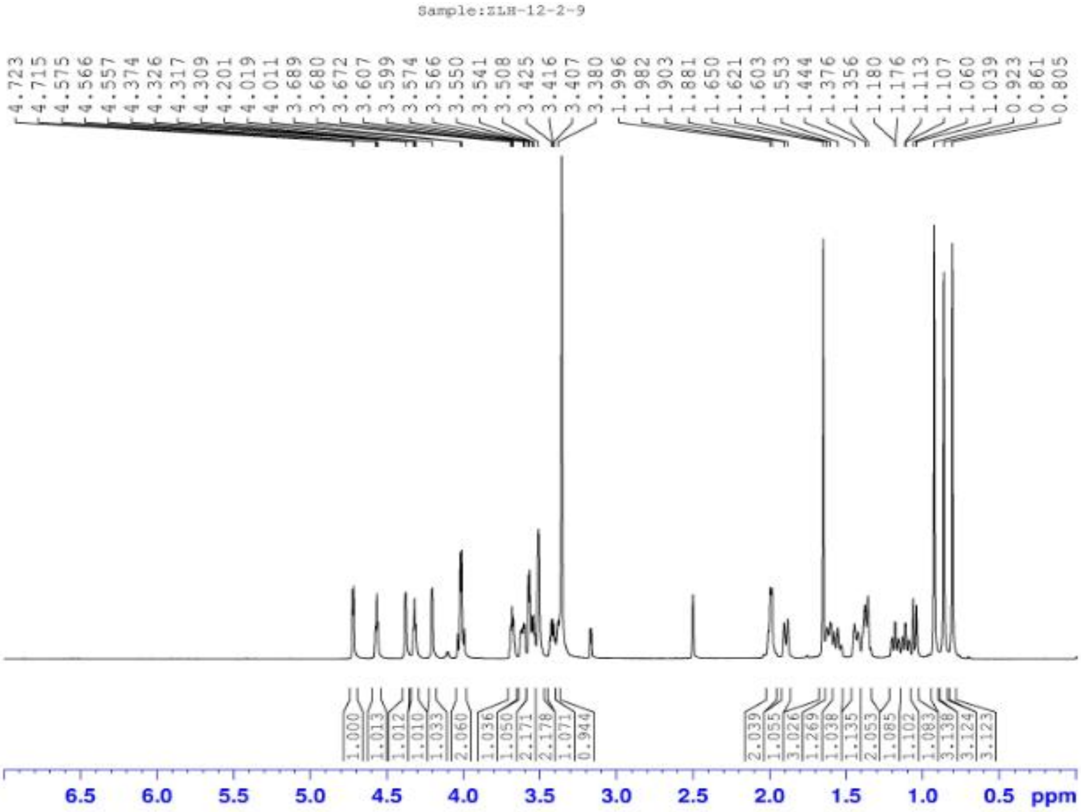

Figure S4. ^{1}H NMR (600 MHz, DMSO-d_6) spectrum of compound 1

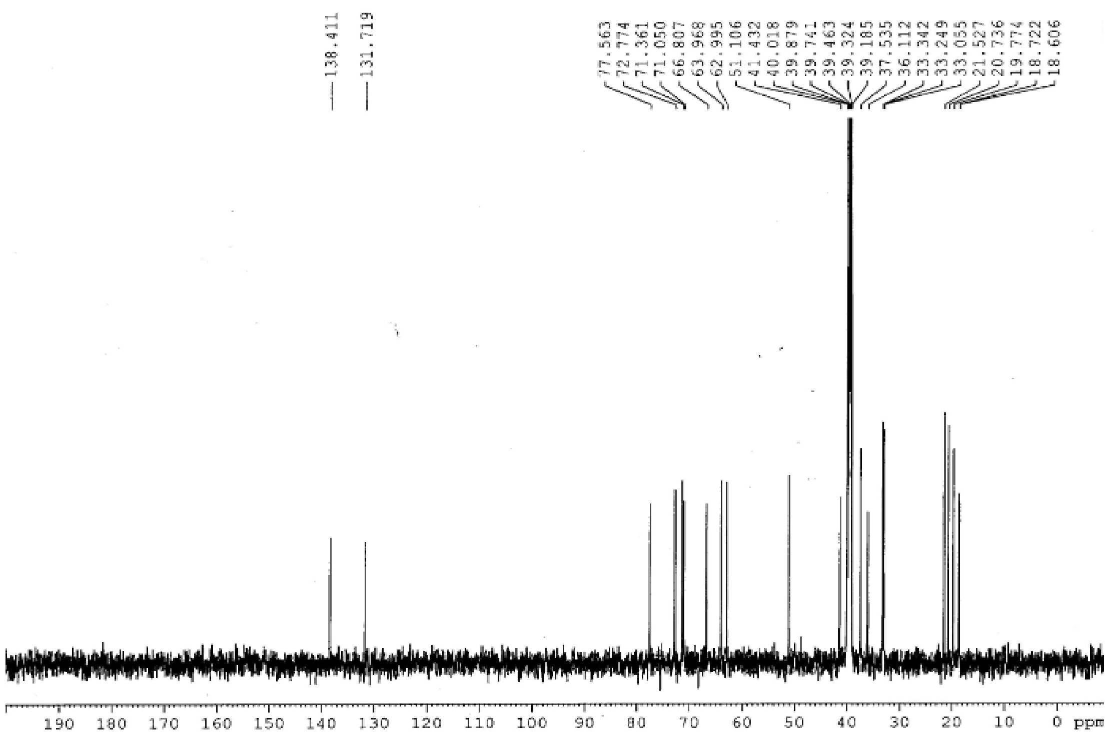

Figure S5. **^{13}C NMR (100 MHz, DMSO-*d*$_6$) spectrum of compound 1**

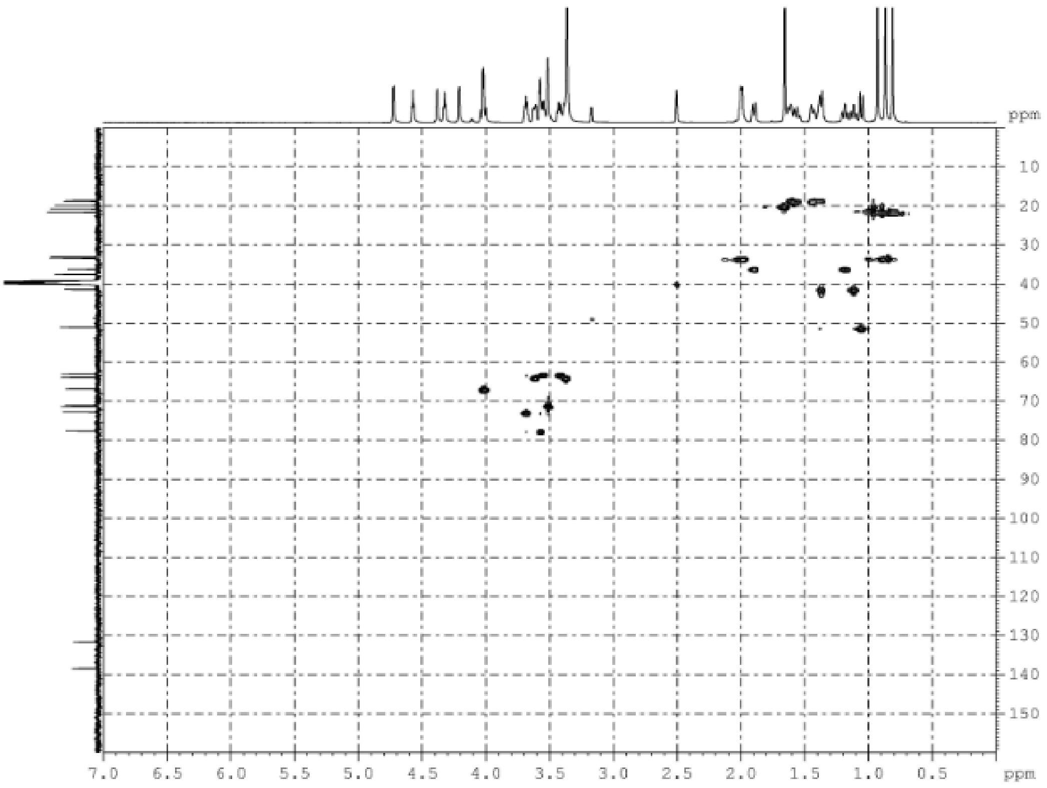

Figure S6. HSQC (600 MHz, DMSO-*d*$_6$) spectrum of compound 1

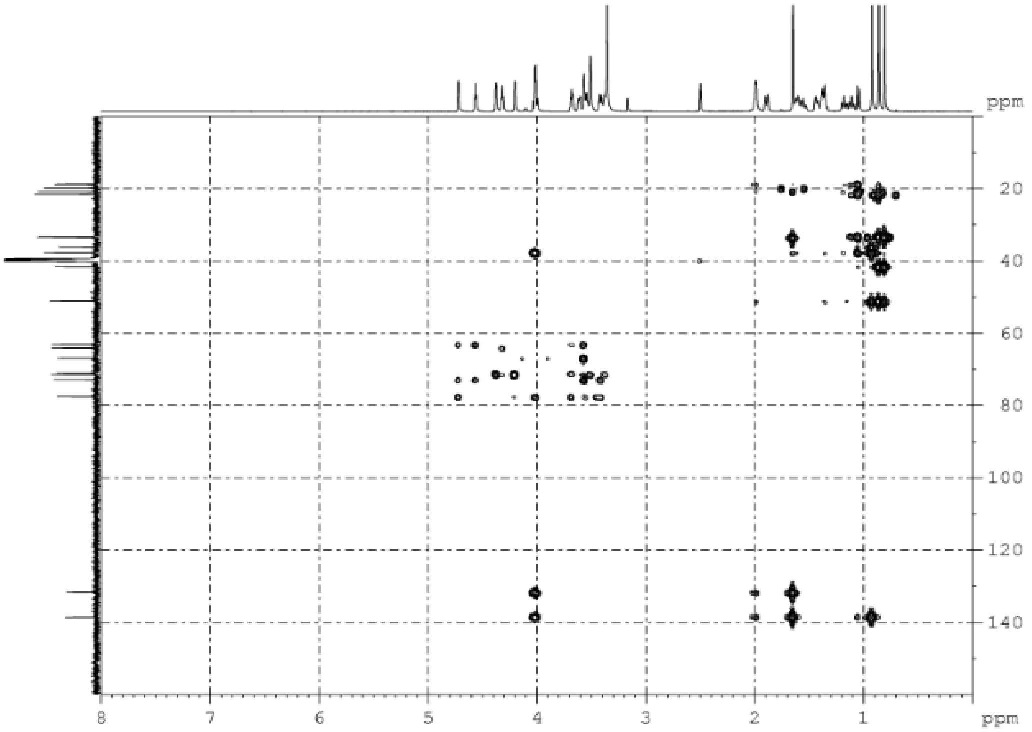

Figure S7. HMBC (600 MHz, DMSO-d_6) spectrum of compound 1

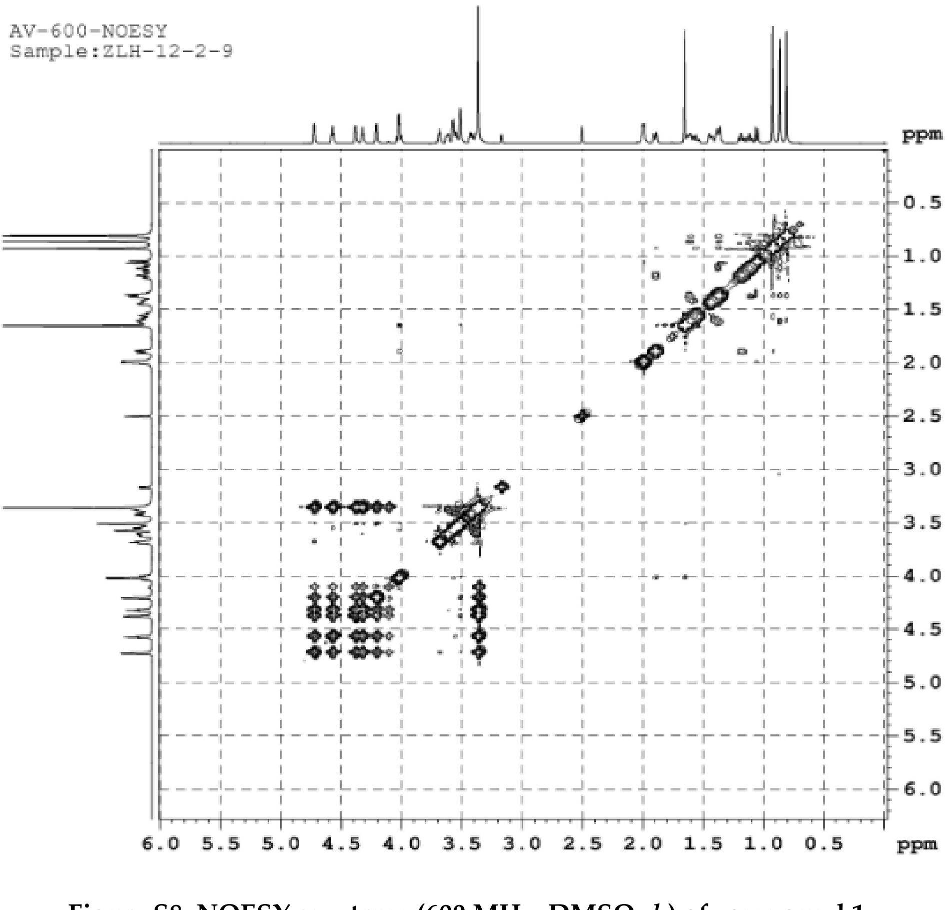

Figure S8. NOESY spectrum (600 MHz, DMSO-d_6) of compound 1

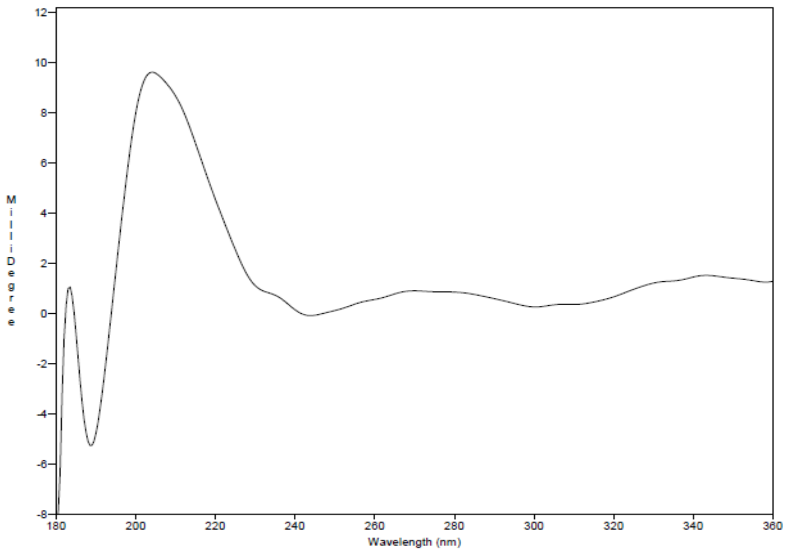

Figure S9. CD spectrum of compound 1 in CH_3CN

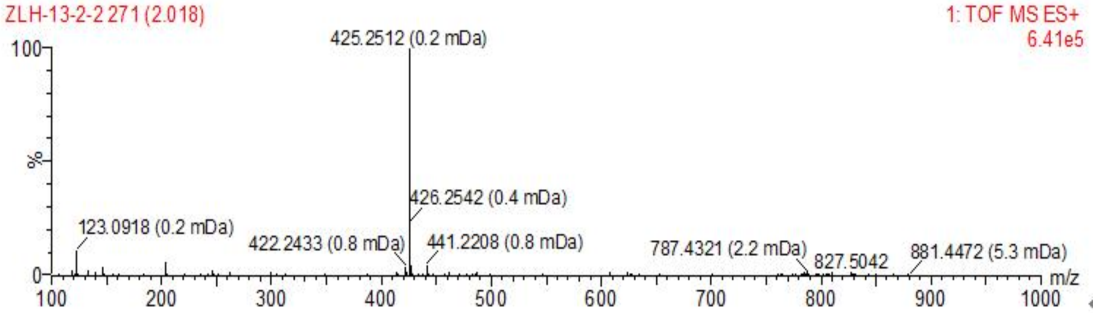

m/*z* 425.2512 [M + Na] $^{+}$

Figure S10. HR ESI-TOF MS spectrum of compound 2

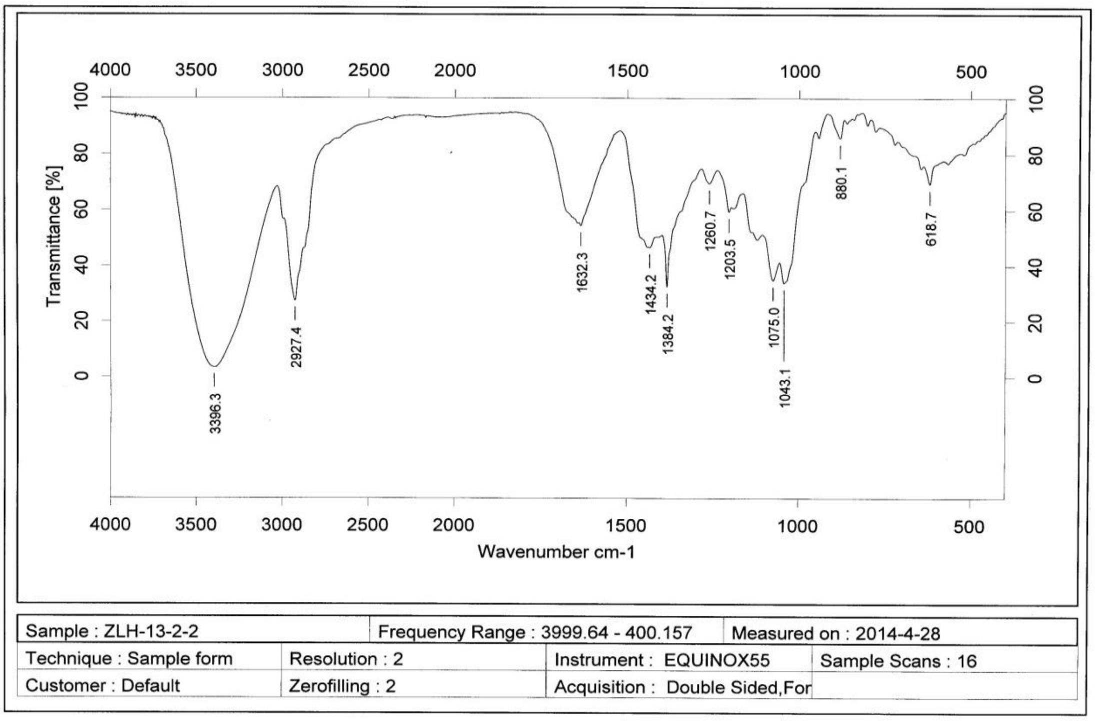

Figure S11. IR spectrum of compound 2

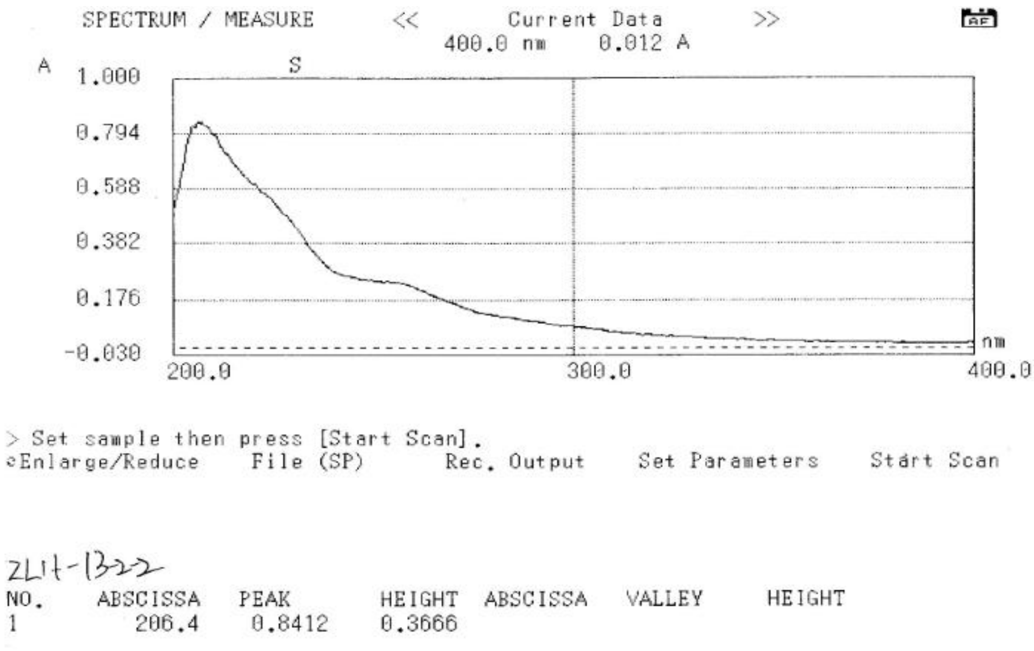

Figure S12. UV spectrum of compound 2

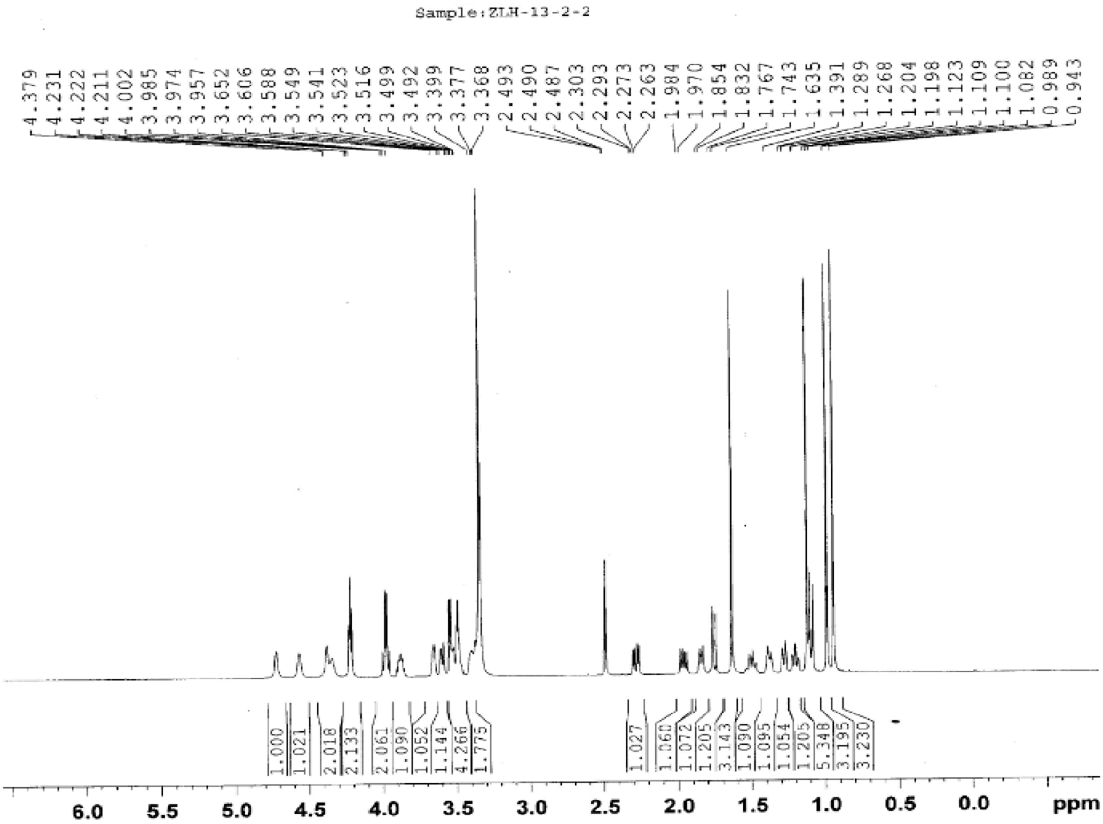

Figure S13. ¹H NMR (600 MHz, DMSO-*d*₆) spectrum of compound 2

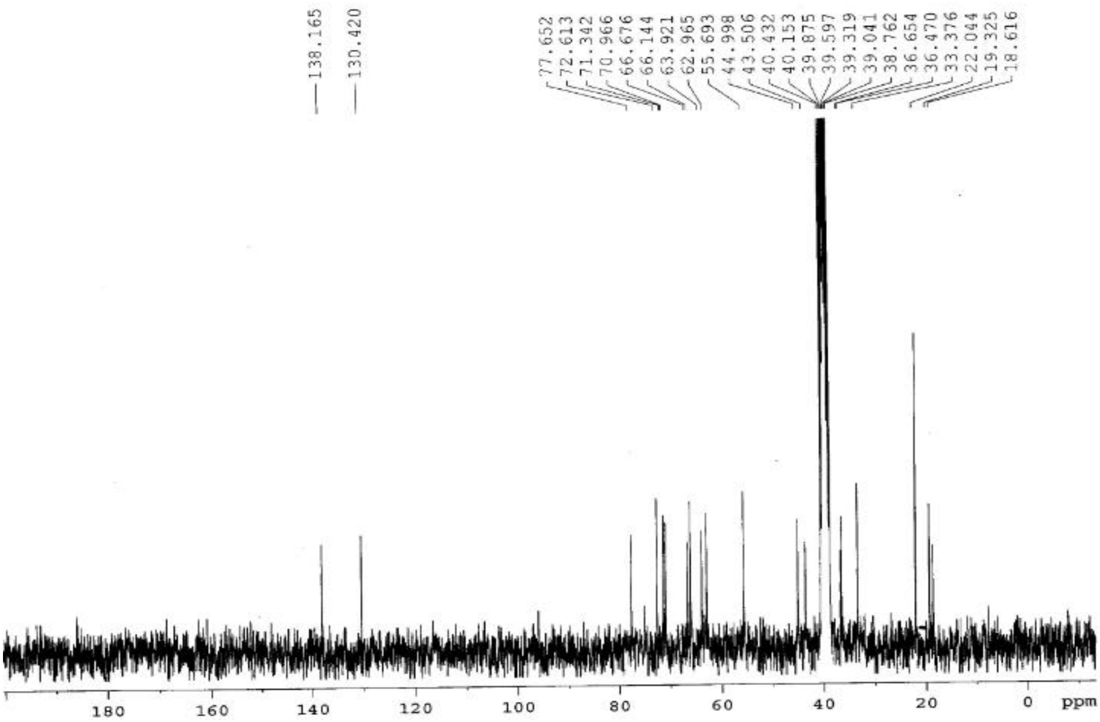

Figure S14. ¹³C NMR (100 MHz, DMSO-*d*₆) spectrum of compound 2

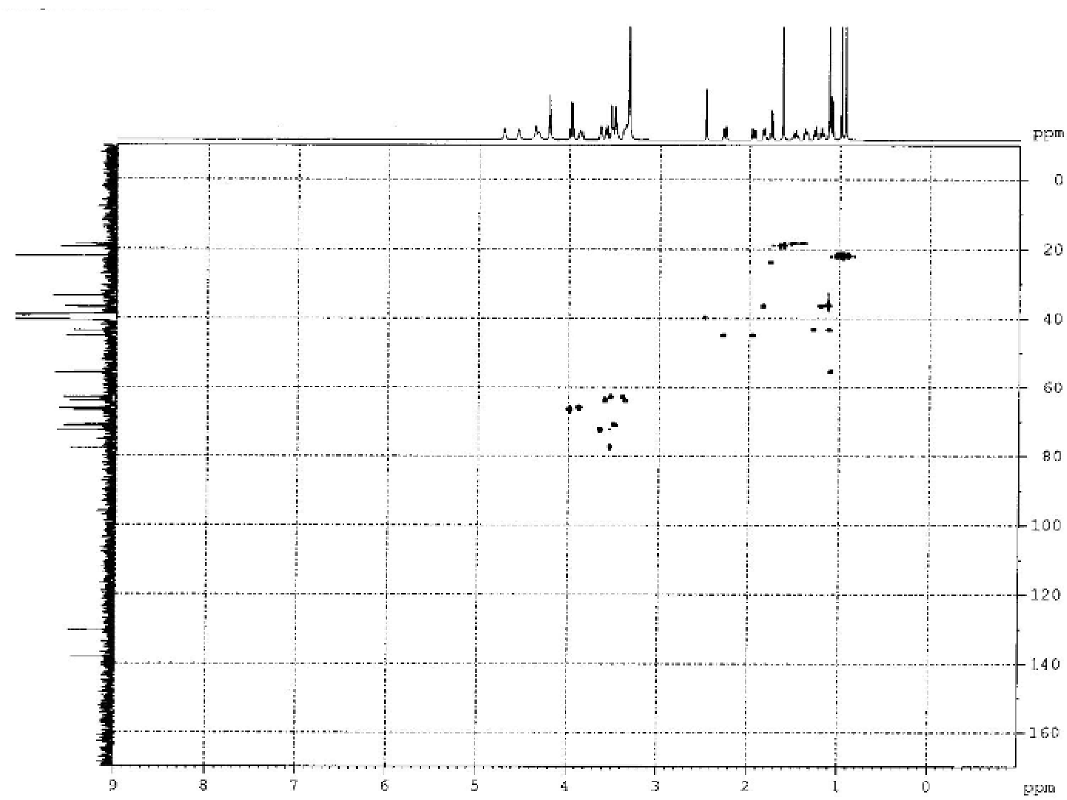

Figure S15. HSQC (600 MHz, DMSO-d_6) spectrum of compound 2

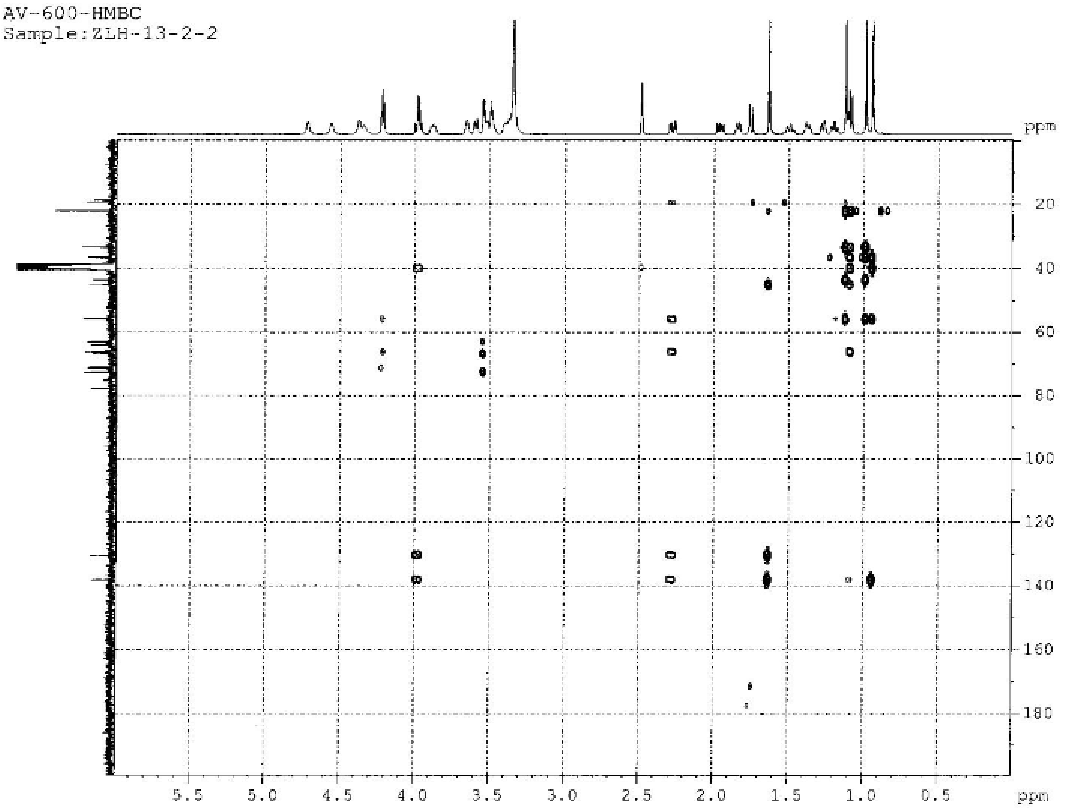

Figure S16. HMBC (600 MHz, DMSO-d_6) spectrum of compound 2

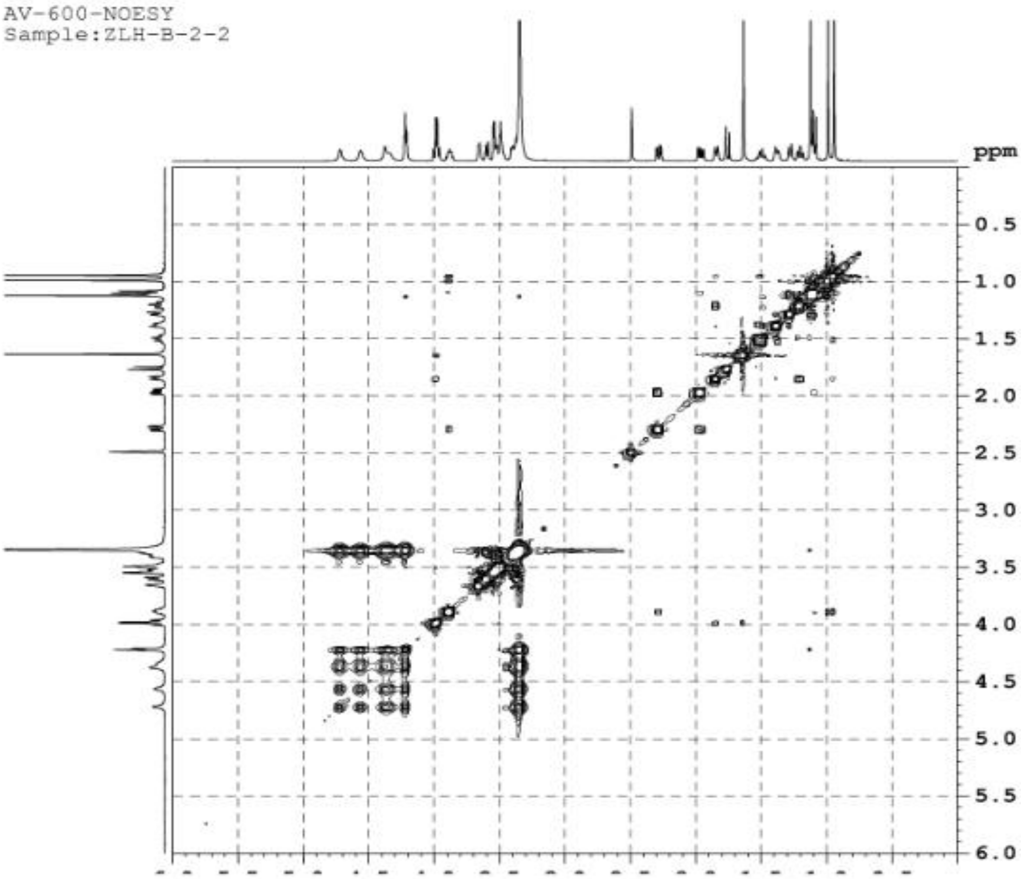

Figure S17. NOESY spectrum (600 MHz, DMSO-*d*6) of compound 2

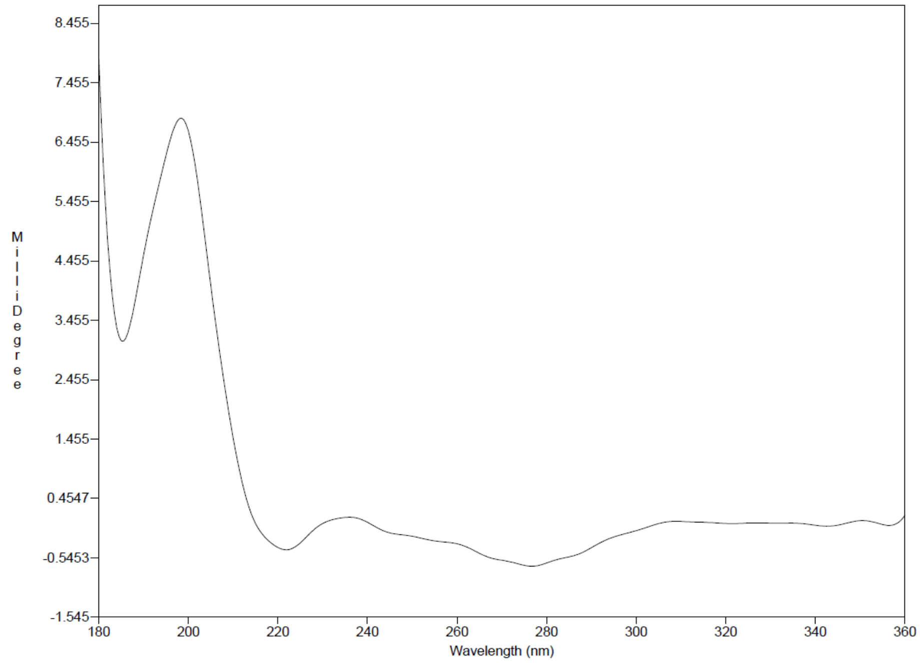

Figure S18. CD spectrum of compound 2 in CH_3CN

Mass Spectrum Molecular Formula Report

Analysis Info

Analysis Name	D:\Data\20141106ceyang\ZLH-13-2-2-CW_1-a,8_01_4174.d	Acquisition Date	11/6/2014 4:21:18 PM
Method	20131026_ceyang.m	Operator	Bruker Customer
Sample Name	ZLH-13-2-2-CW	Instrument / Ser#	micrOTOF-Q 125
Comment			

Acquisition Parameter

Source Type	ESI	Ion Polarity	Positive	Set Nebulizer	1.2 Bar
Focus	Active	Set Capillary	4500 V	Set Dry Heater	180 °C
Scan Begin	50 m/z	Set End Plate Offset	-500 V	Set Dry Gas	8.0 l/min
Scan End	3000 m/z	Set Collision Cell RF	600.0 Vpp	Set Divert Valve	Source

Generate Molecular Formula Parameter

Formula, min.	C16H28O2Na				
Formula, max.					
Measured m/z	275.198	Tolerance	5 ppm	Charge	1
Check Valence	no	Minimum	0	Maximum	0
Nirogen Rule	no	Electron Configuration	both		
Filter H/C Ratio	no	Minimum	0	Maximum	3
Estimate Carbon	yes				

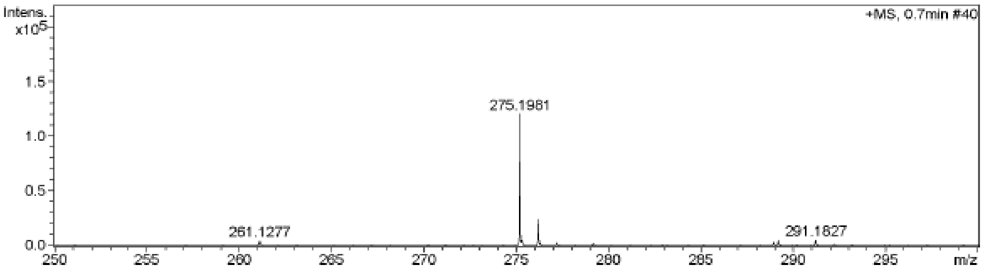

Sum Formula	Sigma	m/z	Err [ppm]	Mean Err [ppm]	Err [mDa]	rdb	N Rule	e⁻
C 16 H 28 Na 1 O 2	0.012	275.1982	0.14	3.96	0.04	2.50	ok	even

Figure S19. HR ESI-TOF MS spectrum of compound 2a

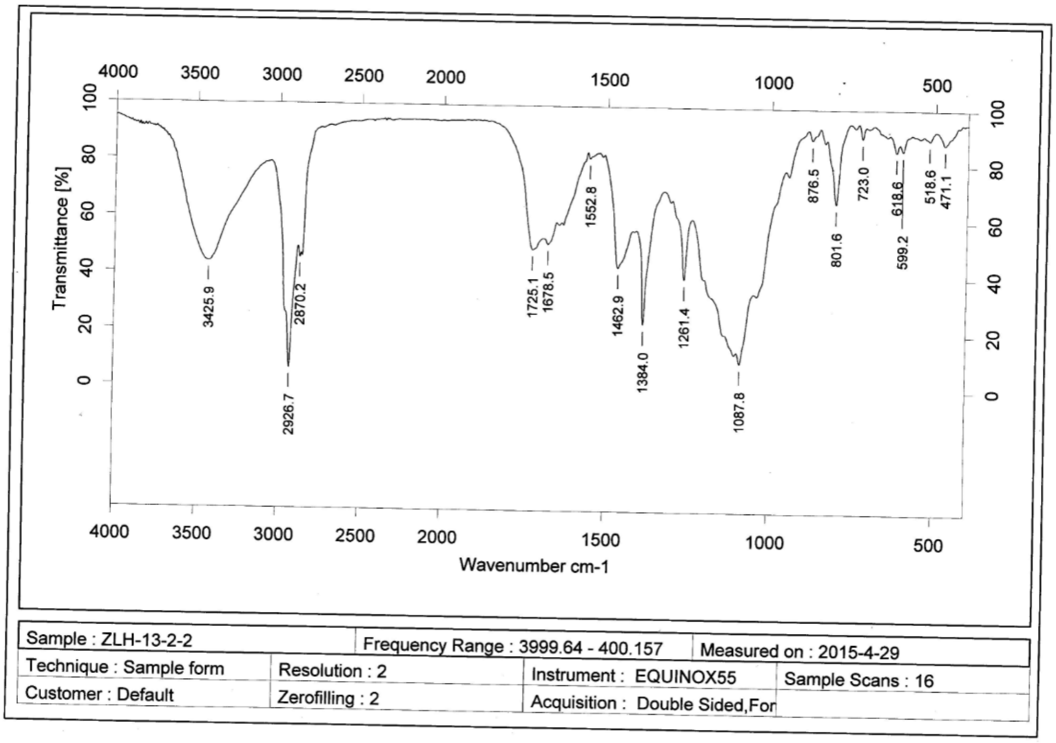

Figure S20. IR spectrum of compound 2a

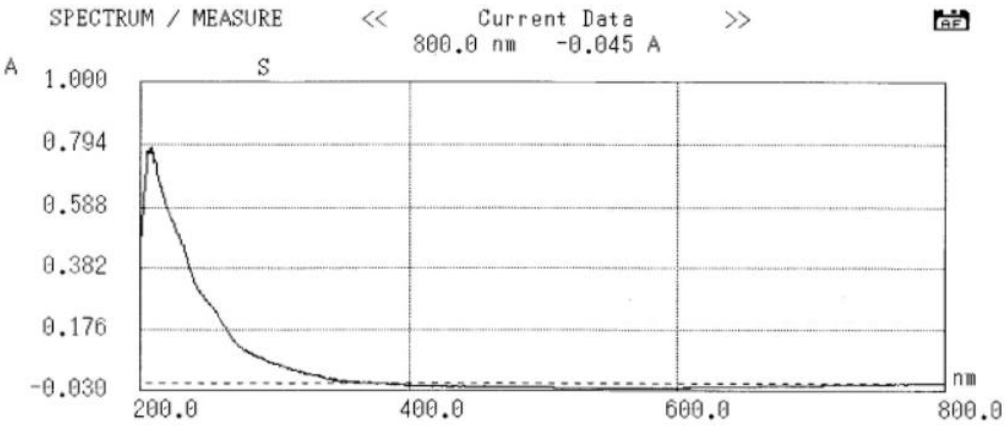

> Set sample then press [Start Scan].
Enlarge/Reduce File (SP) Rec. Output Set Parameters Start Scan

NO.	ABSCISSA	PEAK	HEIGHT	ABSCISSA	VALLEY	HEIGHT
1	207.5	0.7844	0.3495	318.0	0.0331	-0.0141

Figure S21. UV spectrum of compound 2a

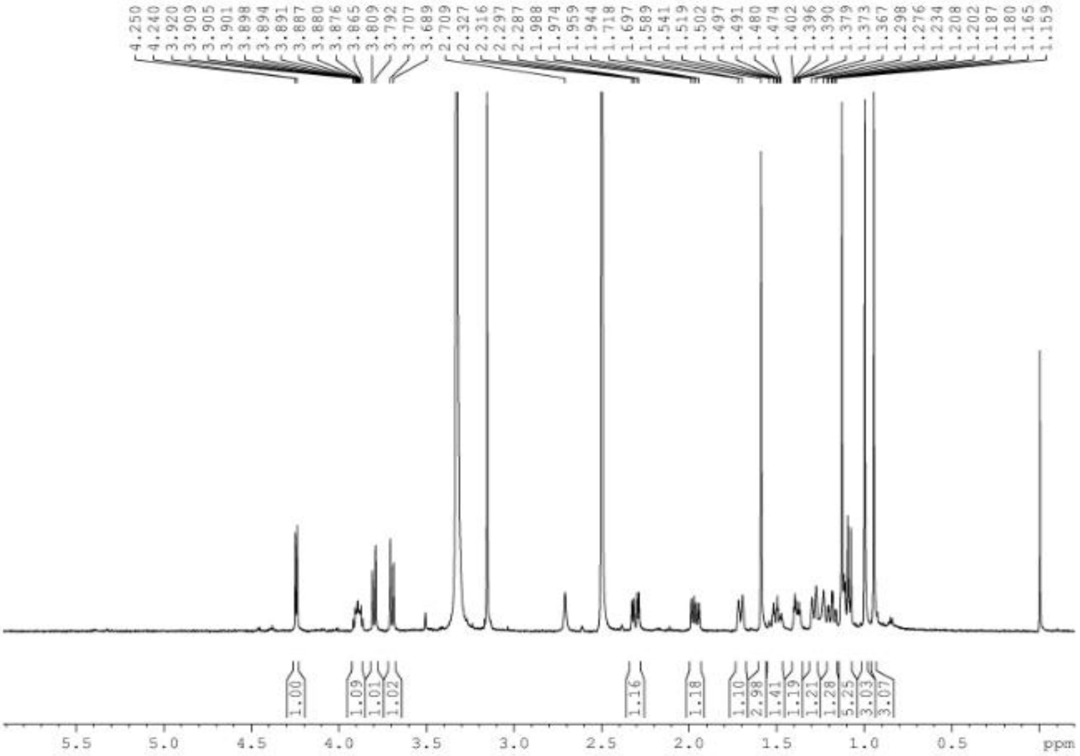

Figure S22. ^{1}H NMR (600 MHz, DMSO-d_6) spectrum of compound 2a

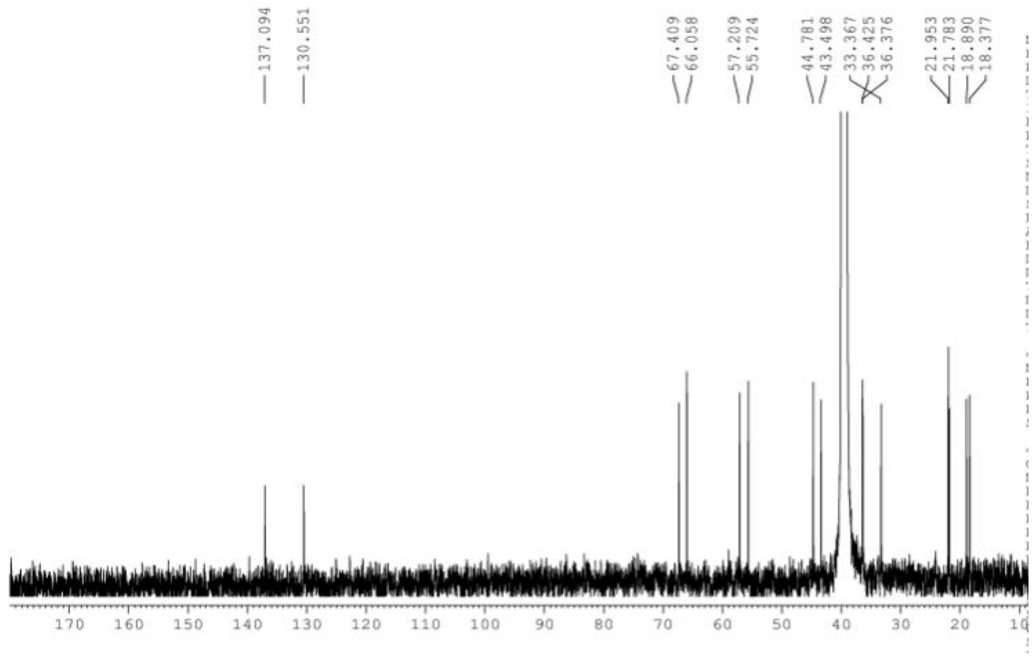

Figure S23. **^{13}C NMR (100 MHz, DMSO-*d*$_6$) spectrum of compound 2a**

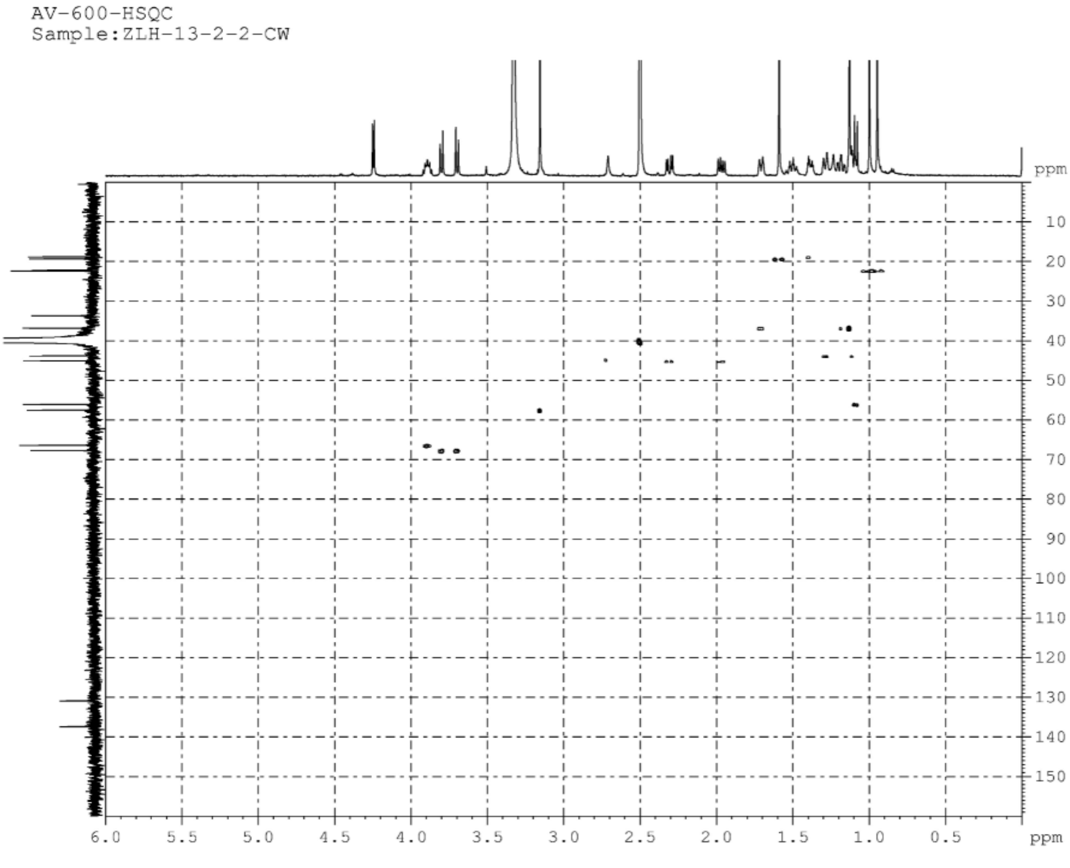

Figure S24. HSQC (600 MHz, DMSO-*d*$_6$) spectrum of compound 2a

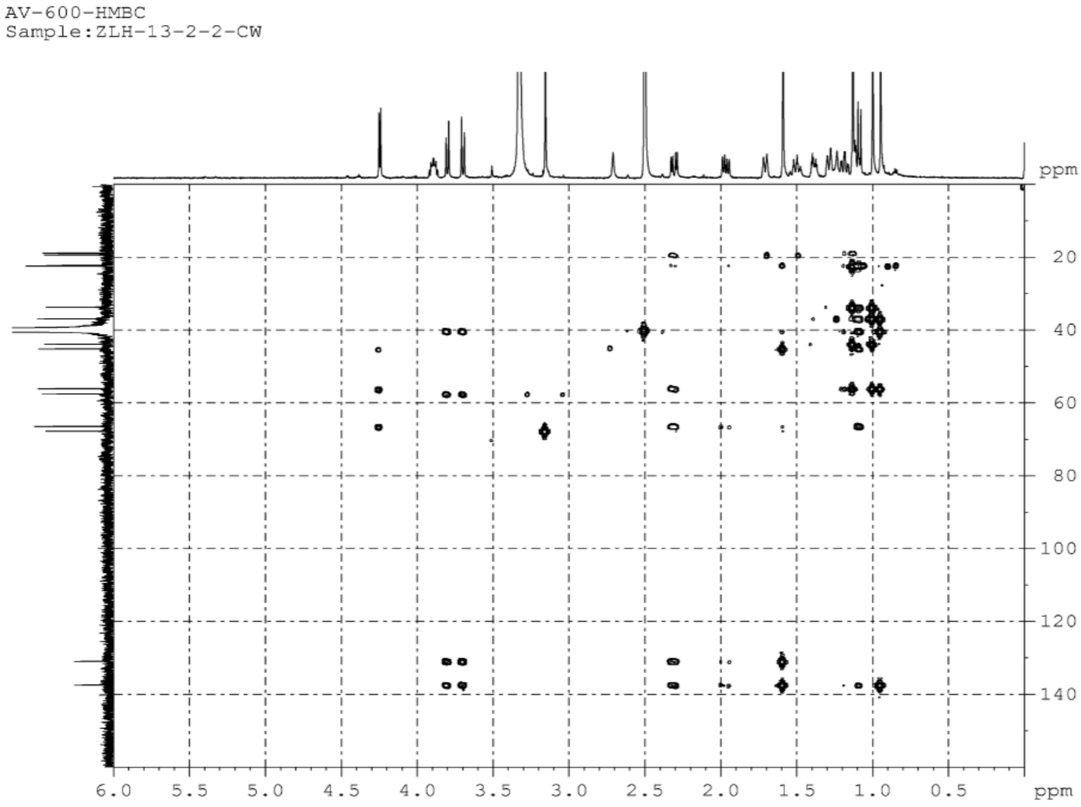

Figure S25. HSBC (600 MHz, DMSO-d_6) spectrum of compound 2a

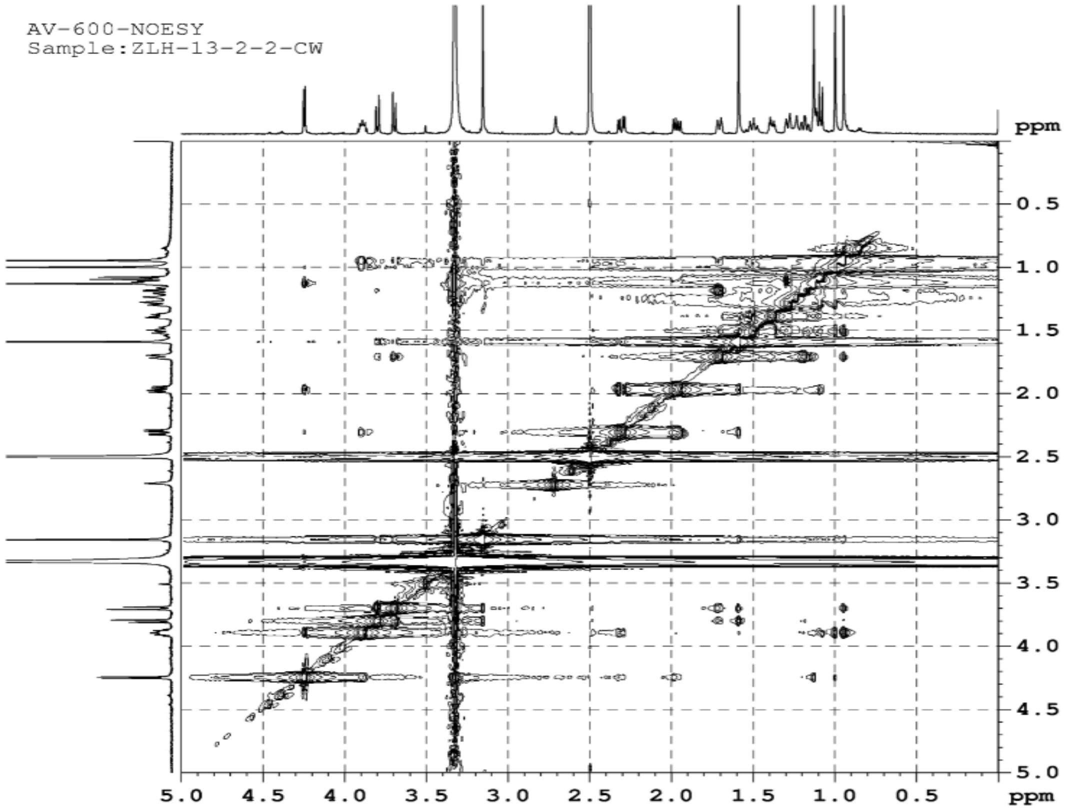

Figure S26. NOESY (600 MHz, DMSO-d_6) spectrum of compound 2a

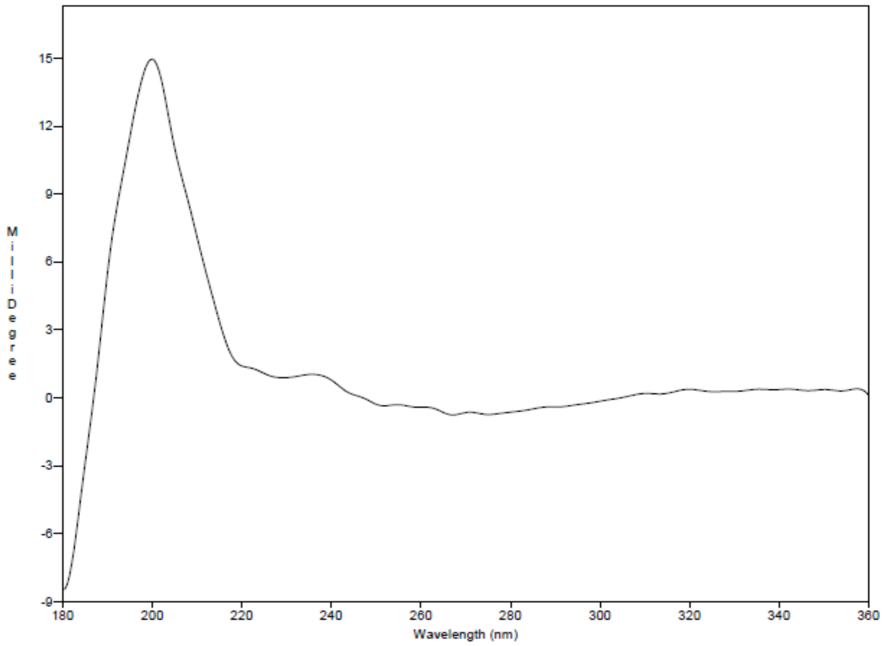

Figure S27. CD spectrum of compound 2a in CH_3CN

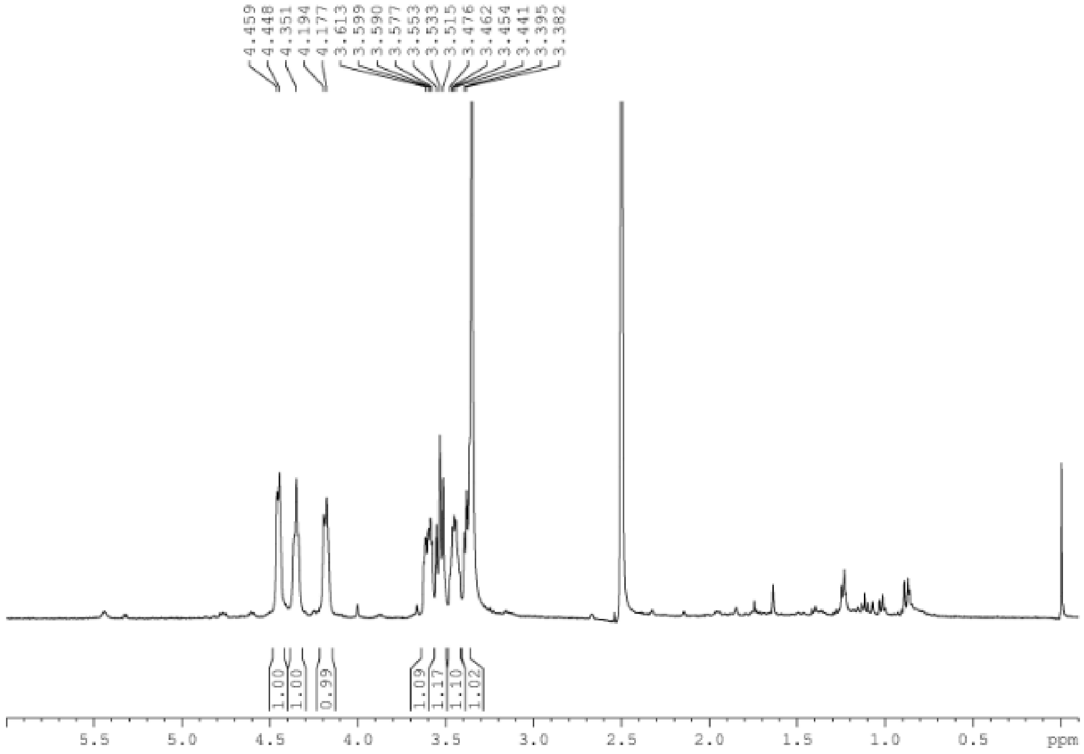

Figure S28. ^{1}H NMR (600 MHz, DMSO-*d*6) spectrum of the hexitol

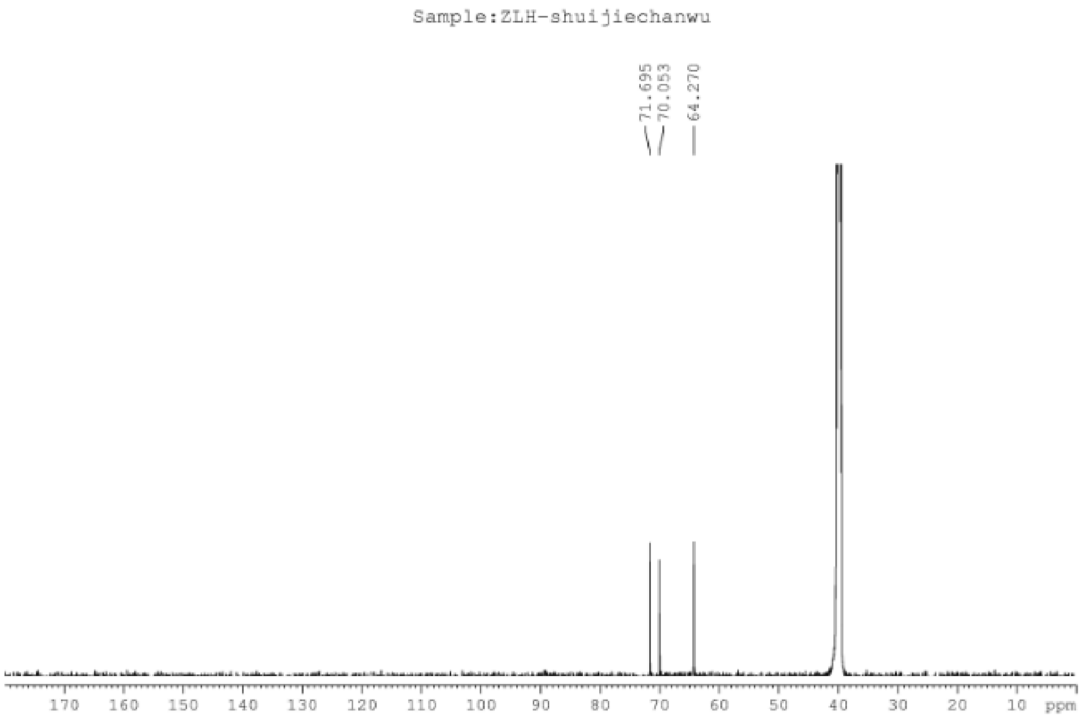

Figure S29. ^{13}C NMR (100 MHz, DMSO-d_6) spectrum of the hexitol

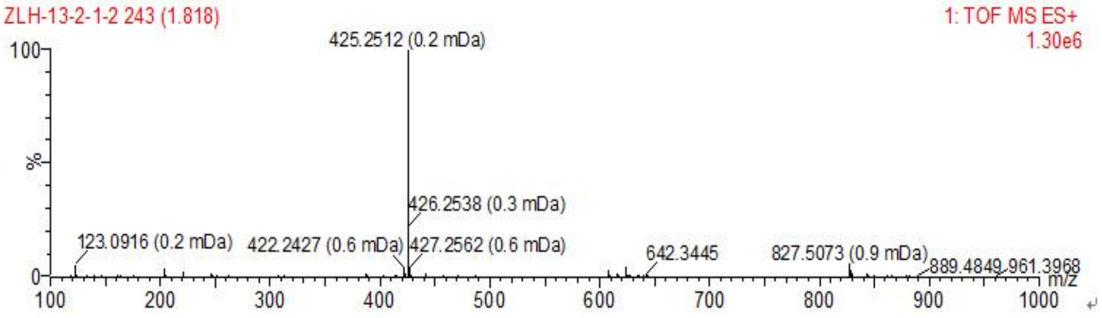

m/z 425.2512 [M + Na] +

Figure S30. HR ESI-TOF MS spectrum of compound 3

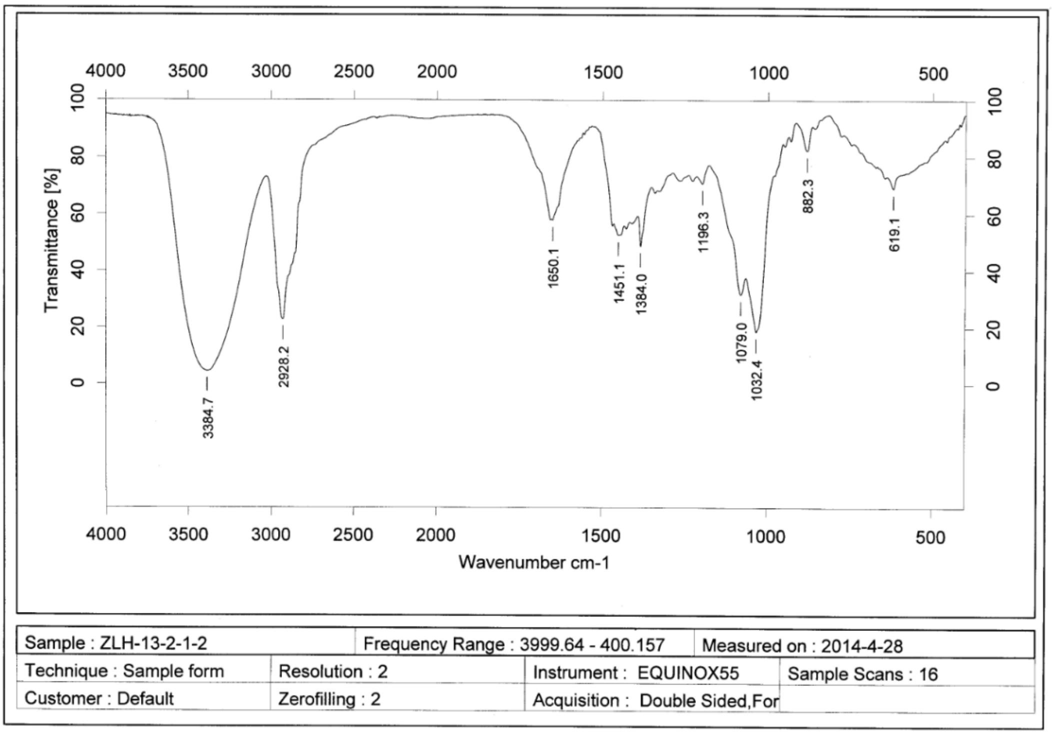

Figure S31. IR spectrum of compound 3

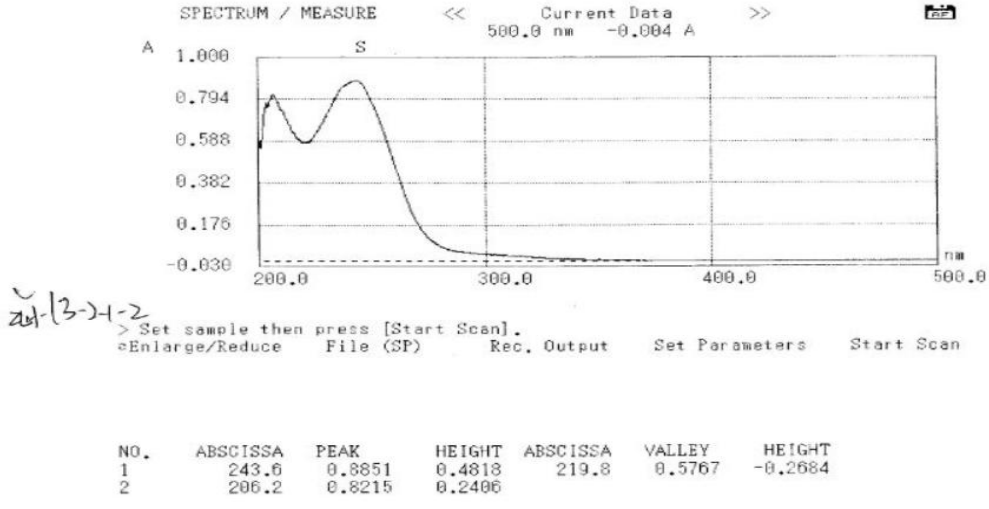

Figure S32. UV spectrum of compound 3

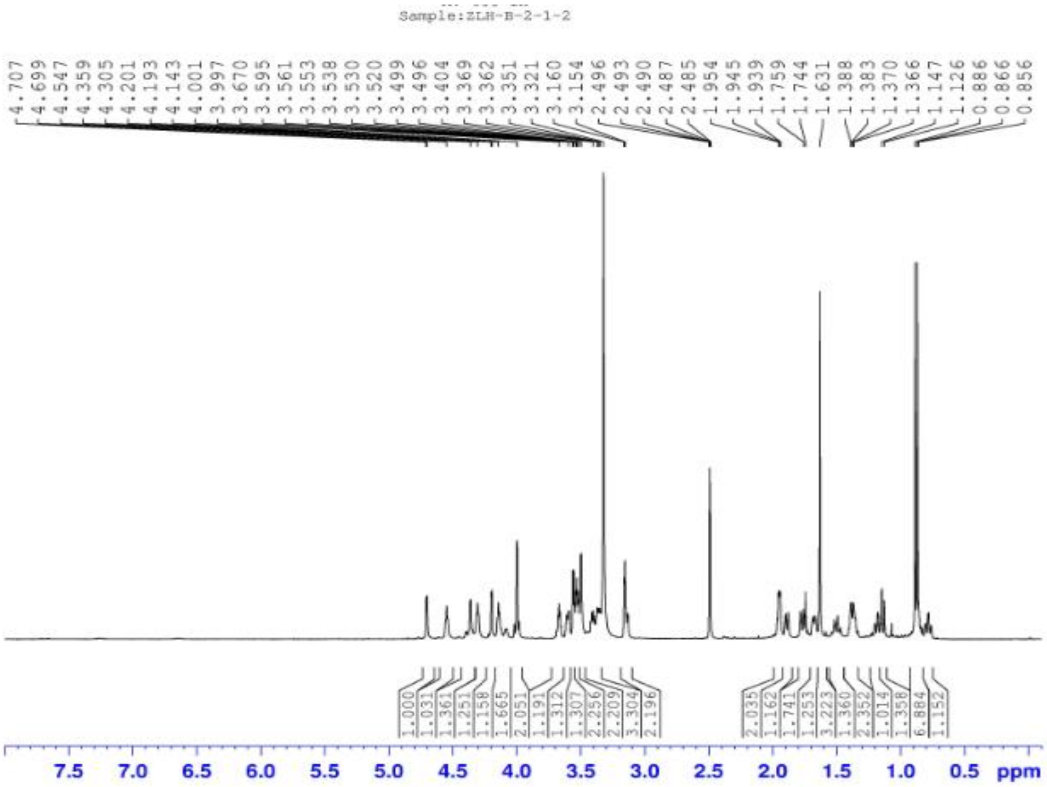

Figure S33. ¹H NMR (600 MHz, DMSO-d_6) spectrum of compound 3

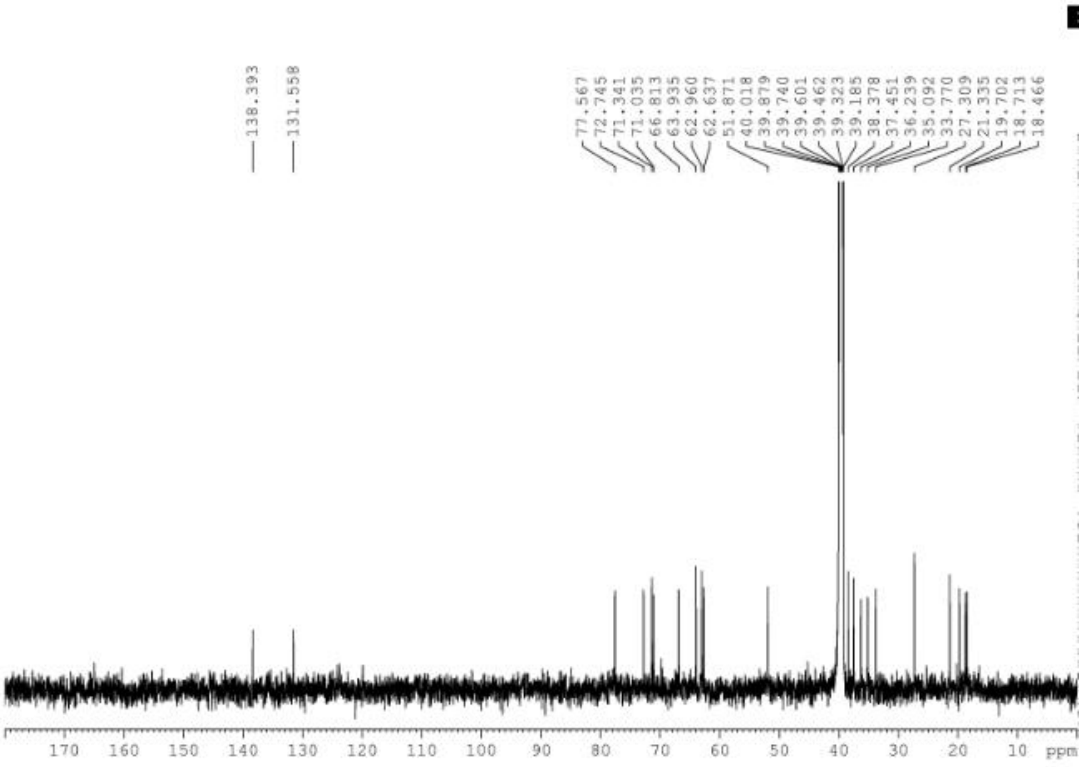

Figure S34. ¹³C NMR (100 MHz, DMSO-d_6) spectrum of compound 3

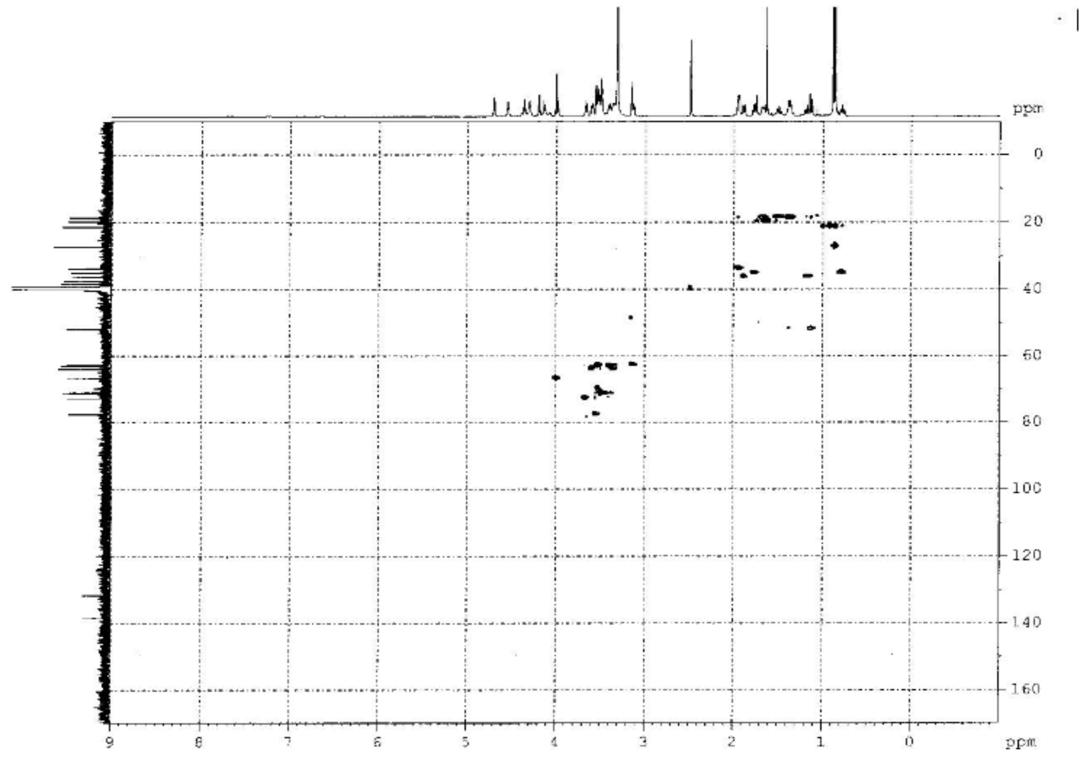

Figure S35. HSQC (600 MHz, DMSO-d_6) spectrum of compound 3

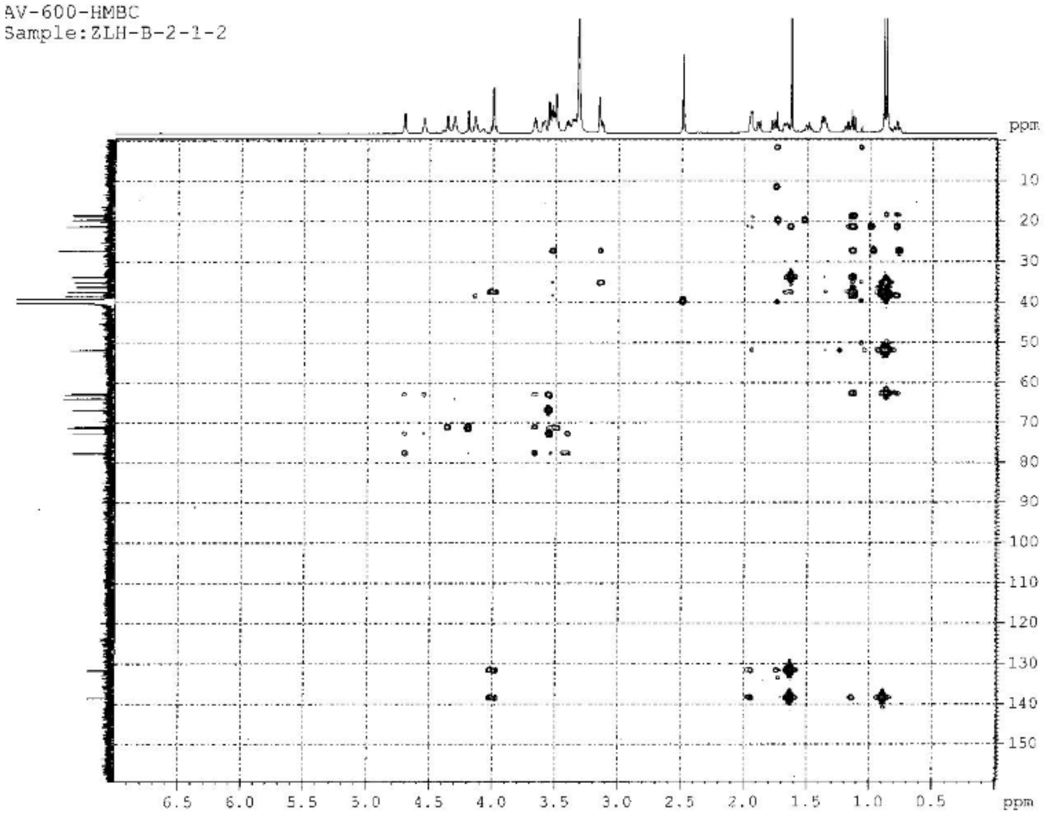

Figure S36. HMBC (600 MHz, DMSO-d_6) spectrum of compound 3

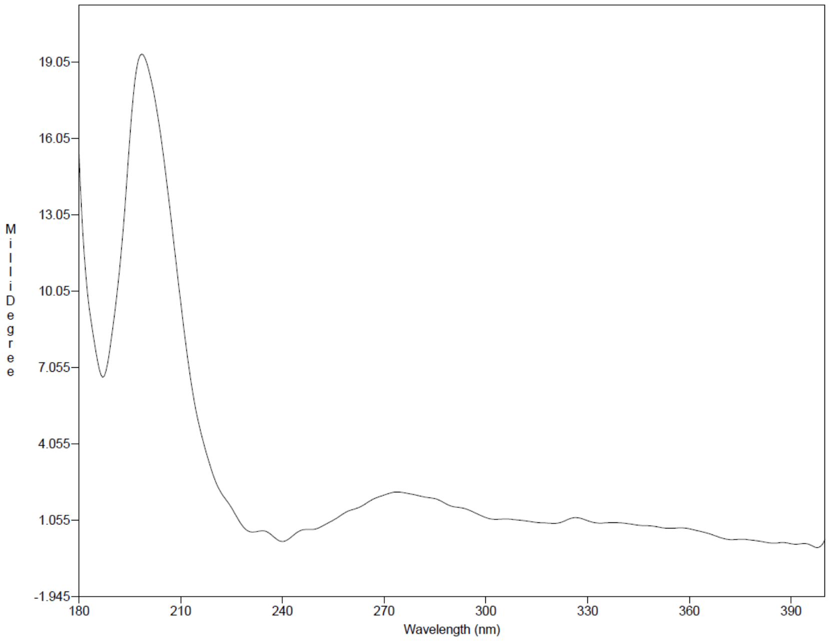

Figure S37. CD spectrum of compound 3 in CH_3CN

Mass Spectrum Molecular Formula Report

**Analysis Info**	D:\Data\20141114CEYANG\ZLH-13-2-1-2-CW.d		
Analysis Name		Acquisition Date	11/14/2014 4:23:32 PM
Method	20131026_ceyang.m	Operator	micrOTOF-Q
Sample Name	ZLH-13-2-1-2-CW	Instrument / Ser#	Bruker Customer 125
Comment			

Acquisition Parameter

Source Type	ESI	Ion Polarity	Positive	Set Nebulizer	0.3 Bar
Focus	Active	Set Capillary	4500 V	Set Dry Heater	180 °C
Scan Begin	50 m/z	Set End Plate Offset	-500 V	Set Dry Gas	4.0 l/min
Scan End	3000 m/z	Set Collision Cell RF	200.0 Vpp	Set Divert Valve	Waste

Generate Molecular Formula Parameter

Formula, min.	C16H28O2Na				
Formula, max.					
Measured m/z	275.199	Tolerance	5 ppm	Charge	1
Check Valence	no	Minimum	0	Maximum	0
Nirogen Rule	no	Electron Configuration	both		
Filter H/C Ratio	no	Minimum	0	Maximum	3
Estimate Carbon	yes				

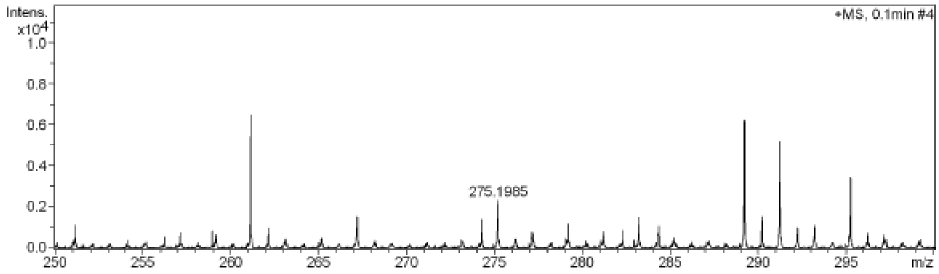

Sum Formula	Sigma	m/z	Err [ppm]	Mean Err [ppm]	Err [mDa]	rdb	N Rule	e⁻
C 16 H 28 Na 1 O 2	0.021	275.1982	-1.32	6.66	-0.36	2.50	ok	even

Figure S38. HR ESI-TOF MS spectrum of compound 3a

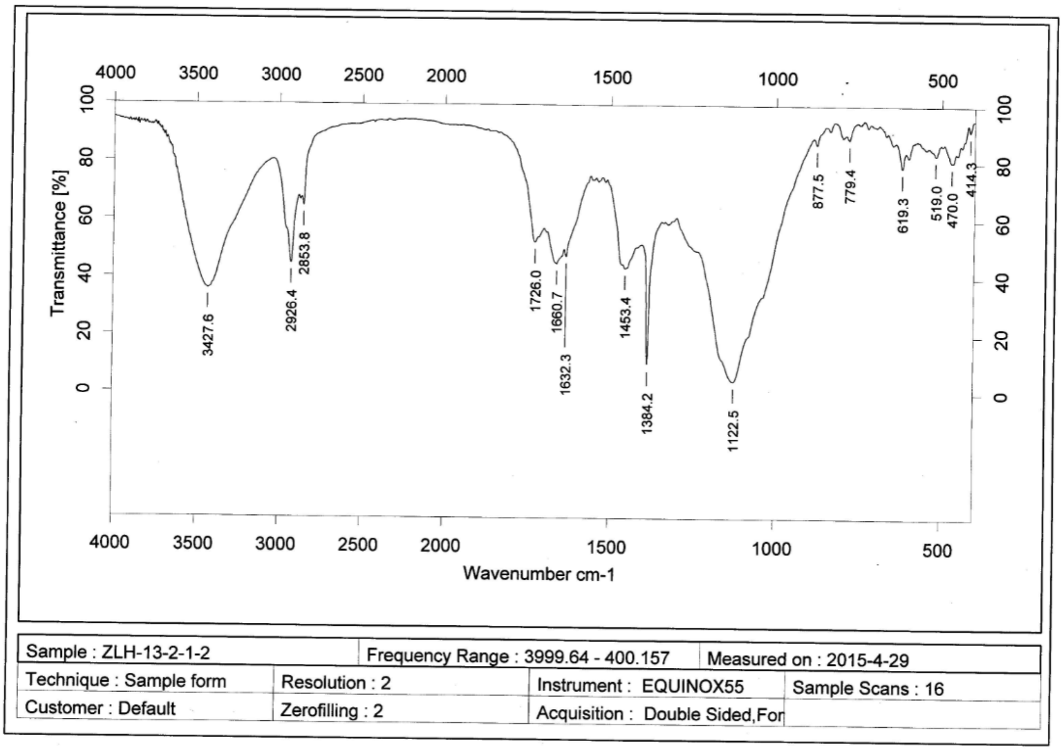

Figure S39. IR spectrum of compound 3a

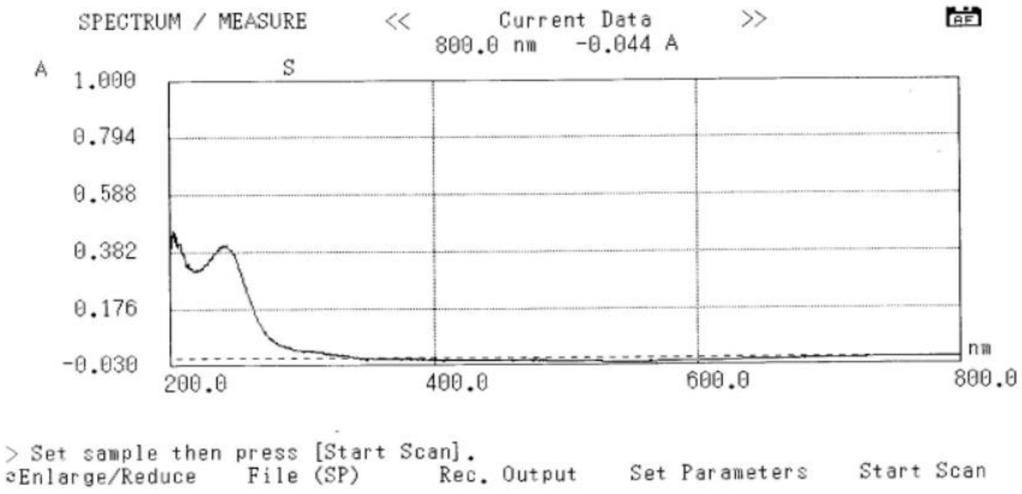

NO.	ABSCISSA	PEAK	HEIGHT	ABSCISSA	VALLEY	HEIGHT
1	240.5	0.4090	0.1768	306.5	0.0217	-0.0117
2				219.0	0.3124	-0.0927

Figure S40. UV spectrum of compound 3a

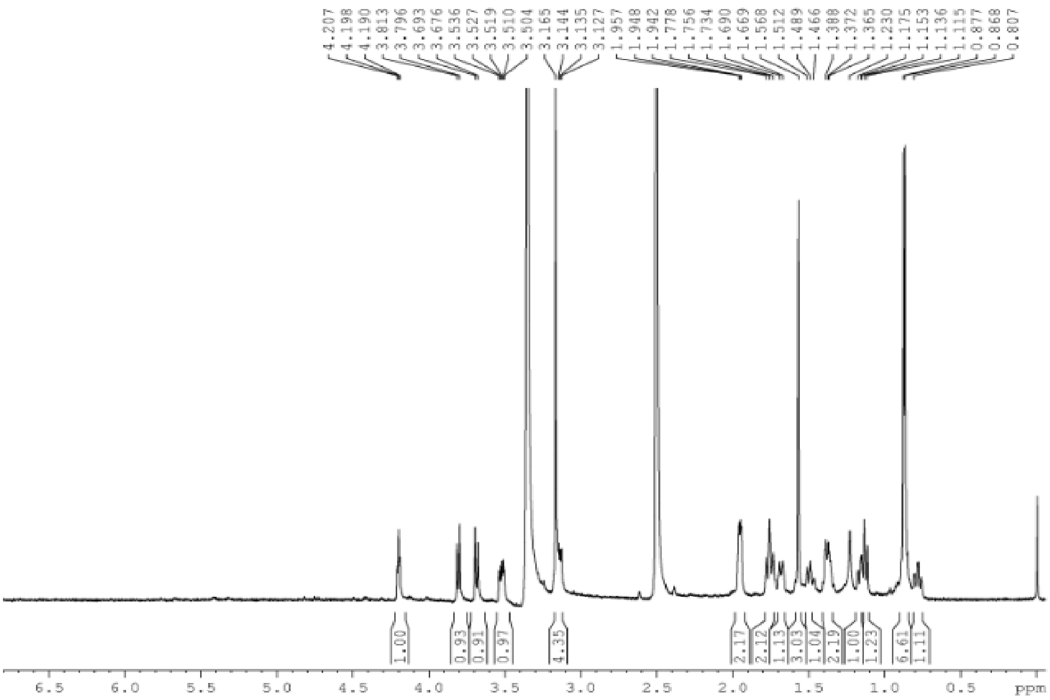

Figure S41. **¹H NMR (600 MHz, DMSO-*d*₆) spectrum of compound 3a**

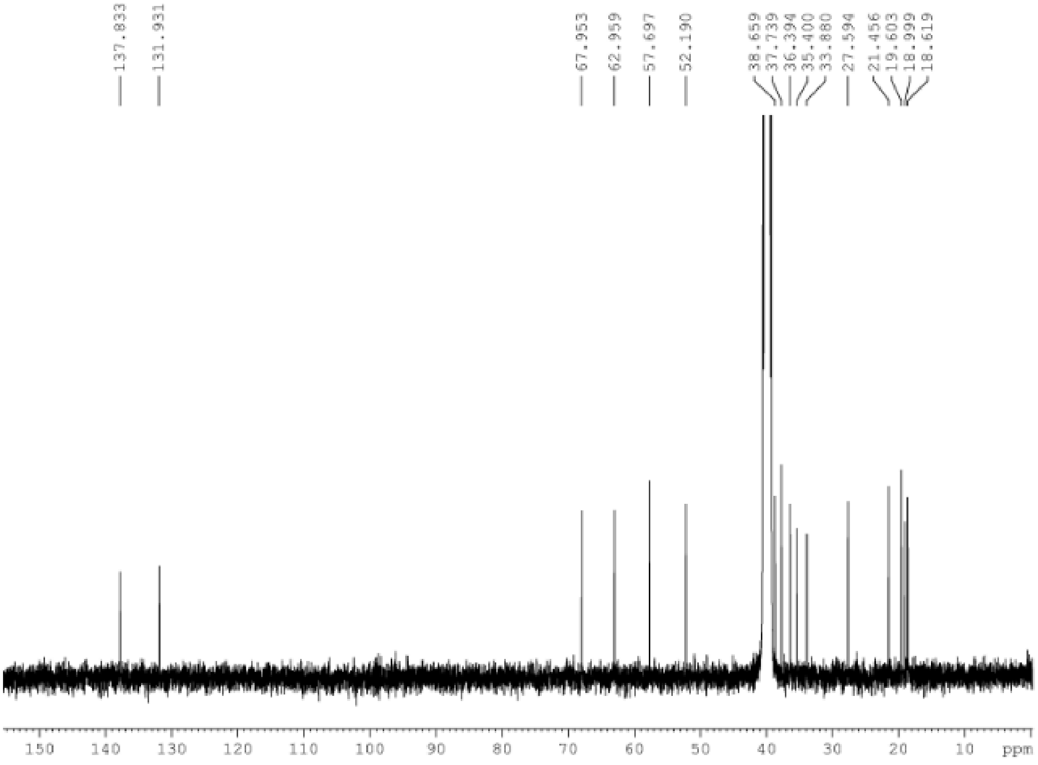

Figure S42. ^{13}C NMR (100 MHz, DMSO-*d*$_6$) spectrum of compound 3a

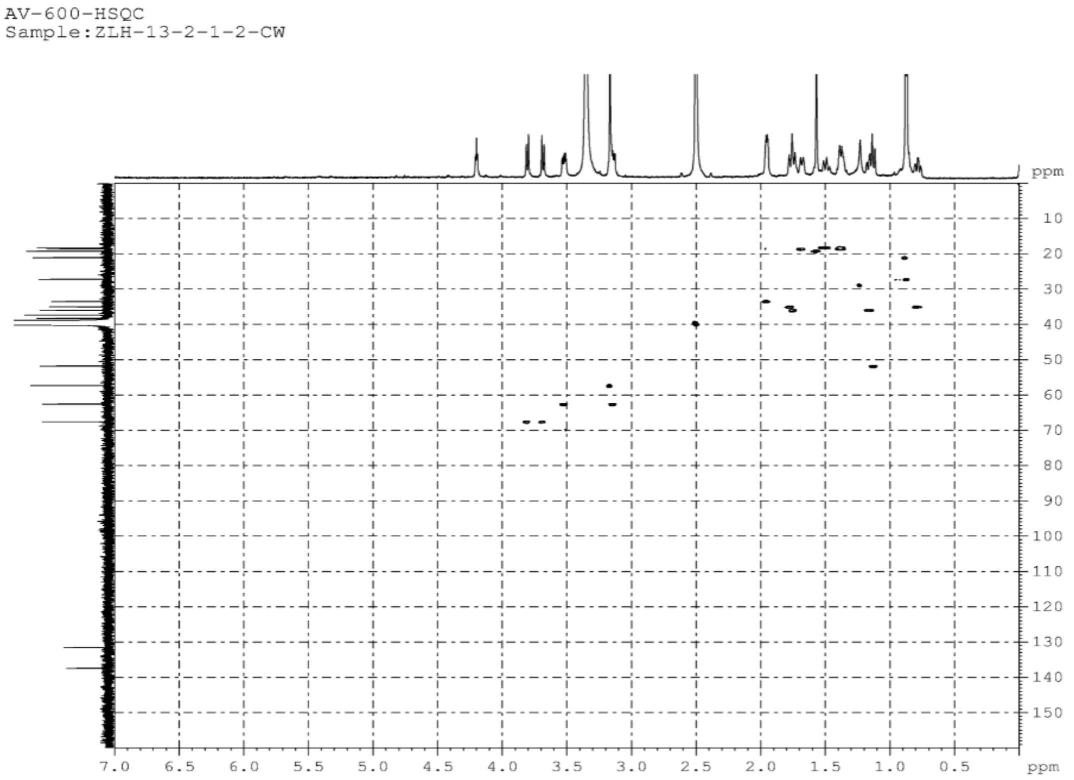

Figure S43. HSQC (600 MHz, DMSO-d_6) spectrum of compound 3a

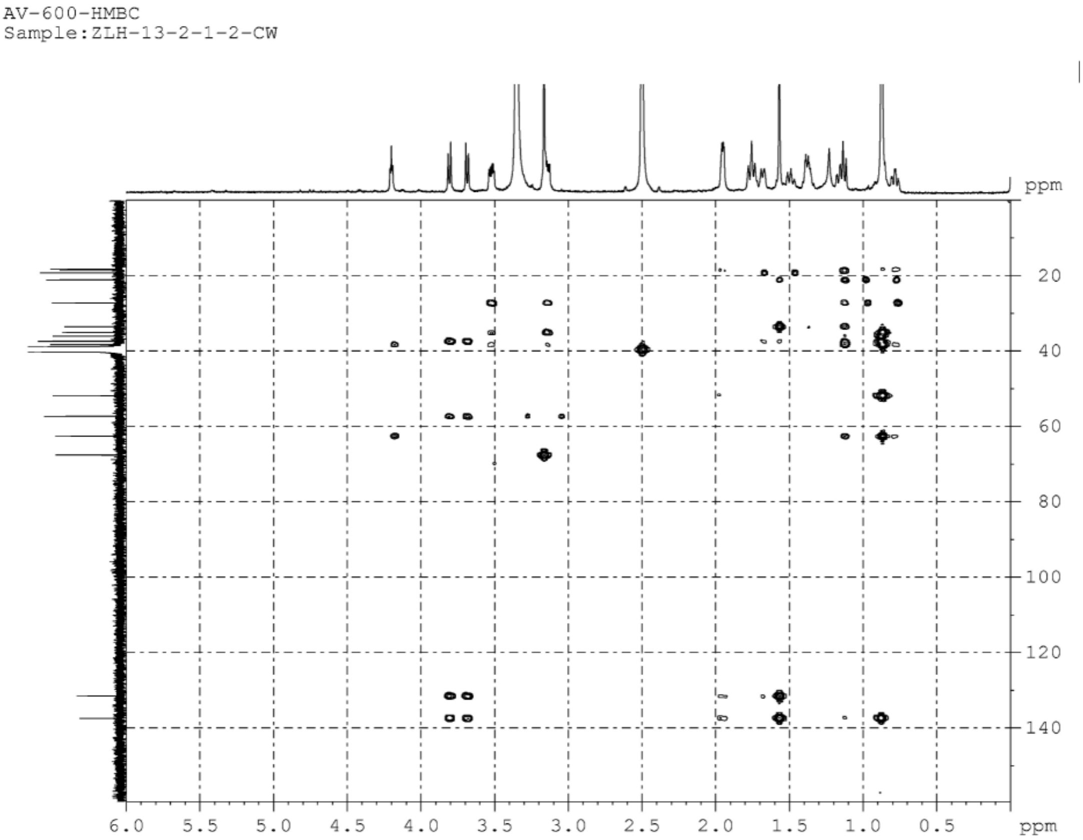

Figure S44. HSBC (600 MHz, DMSO-d_6) spectrum of compound 3a

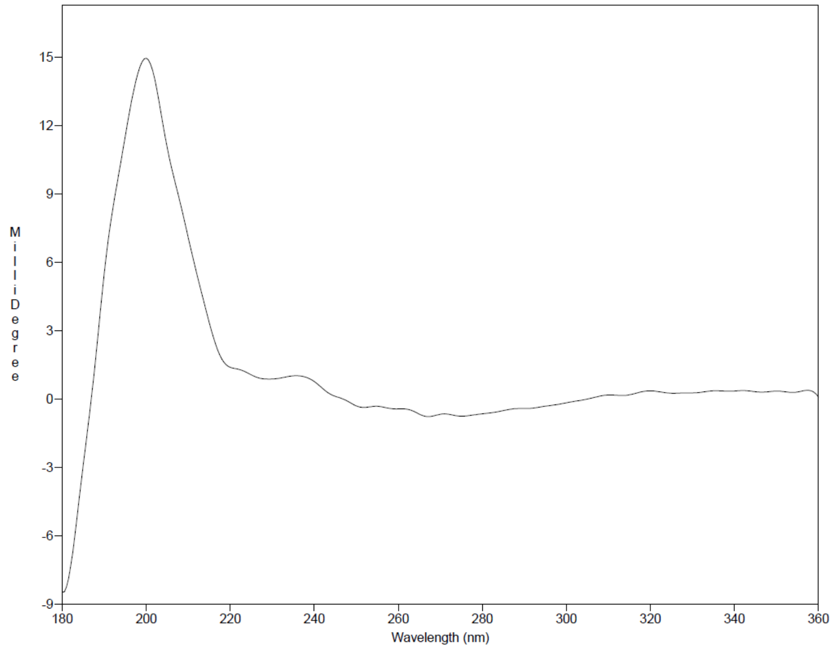

Figure S45. CD spectrum of compound 3a in CH_3CN

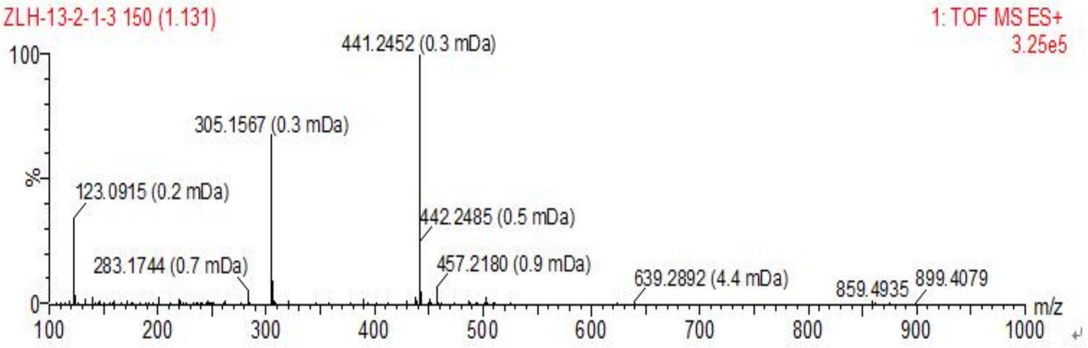

m/z 441.2452 [M + Na] +

Figure S46. HR ESI-TOF MS spectrum of compound 4

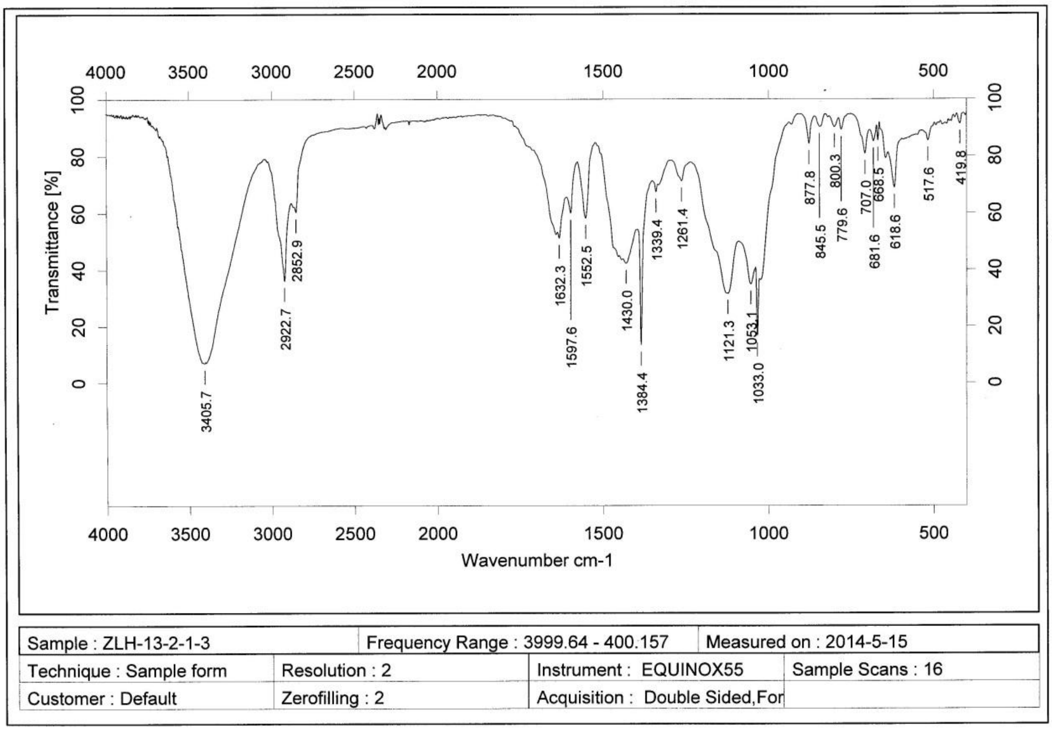

Figure S47. IR spectrum of compound 4

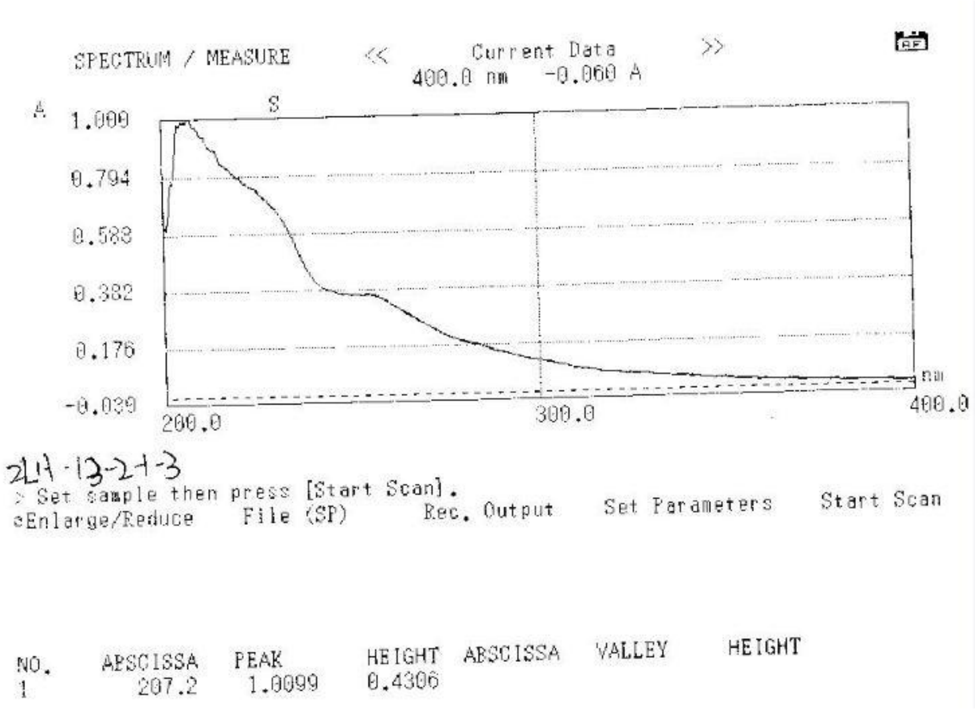

Figure S48. UV spectrum of compound 4

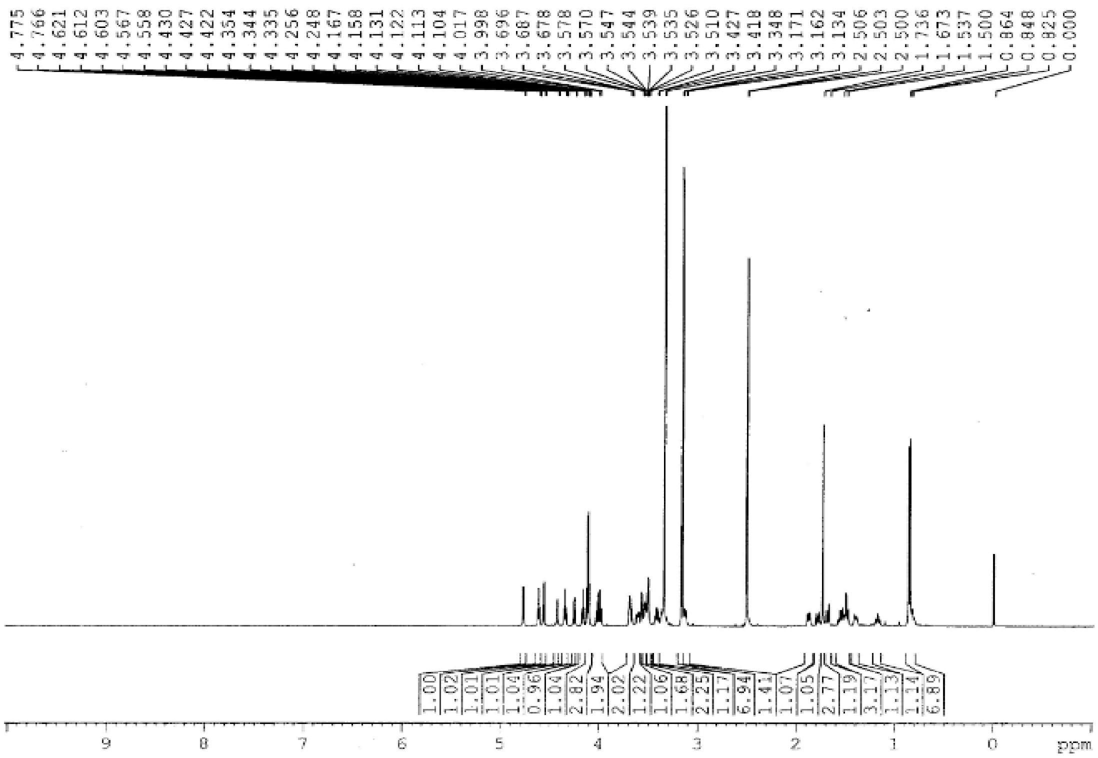

Figure S49. ^{1}H **NMR (600 MHz, DMSO-*d*$_6$) spectrum of compound 4**

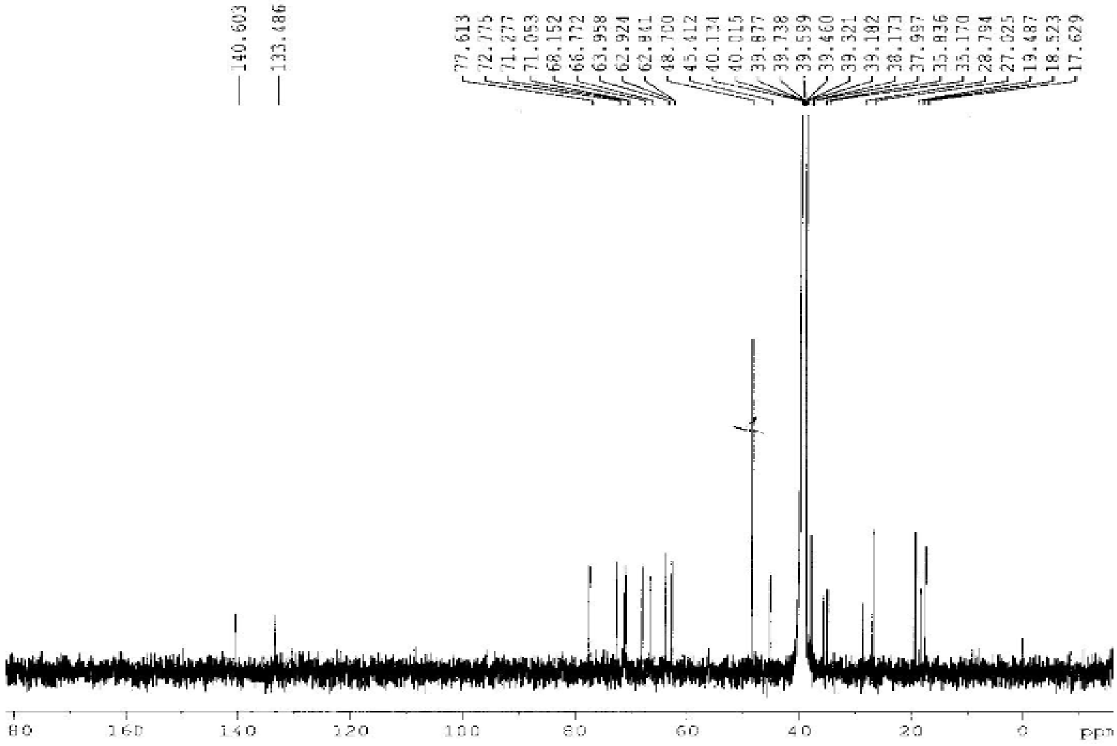

Figure S50. ^{13}C **NMR (100 MHz, DMSO-*d*$_6$) spectrum of compound 4**

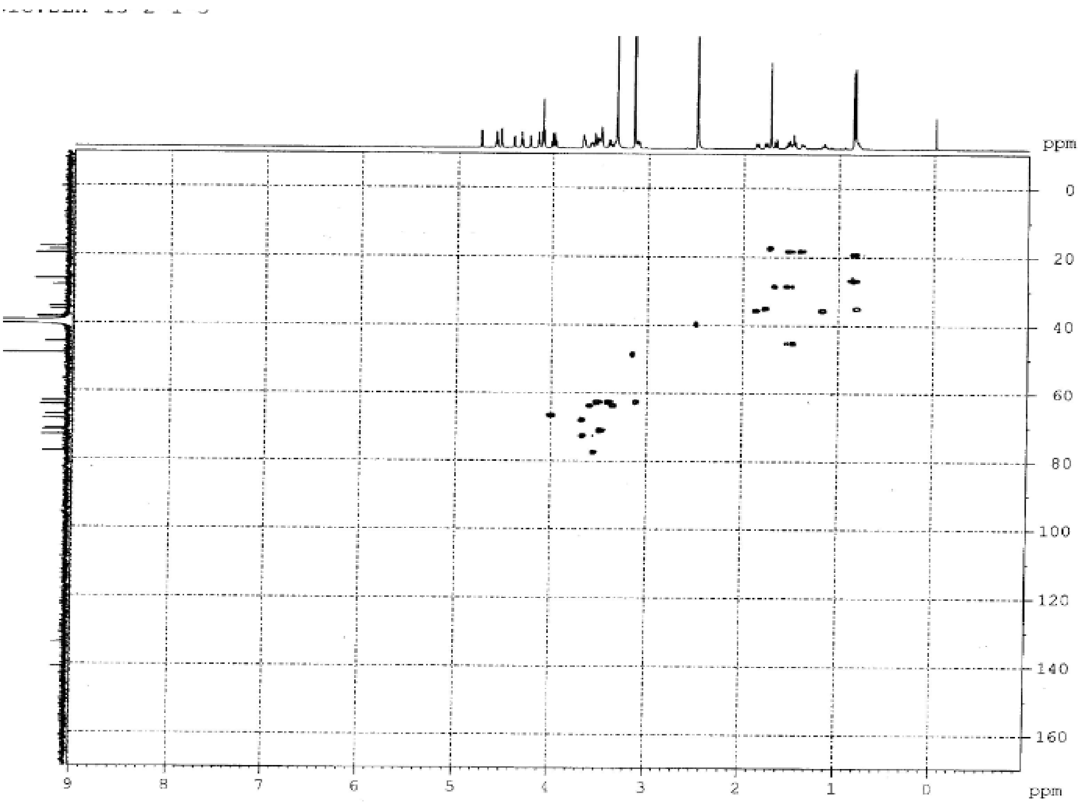

Figure S51. HSQC (600 MHz, DMSO-d_6) spectrum of compound 4

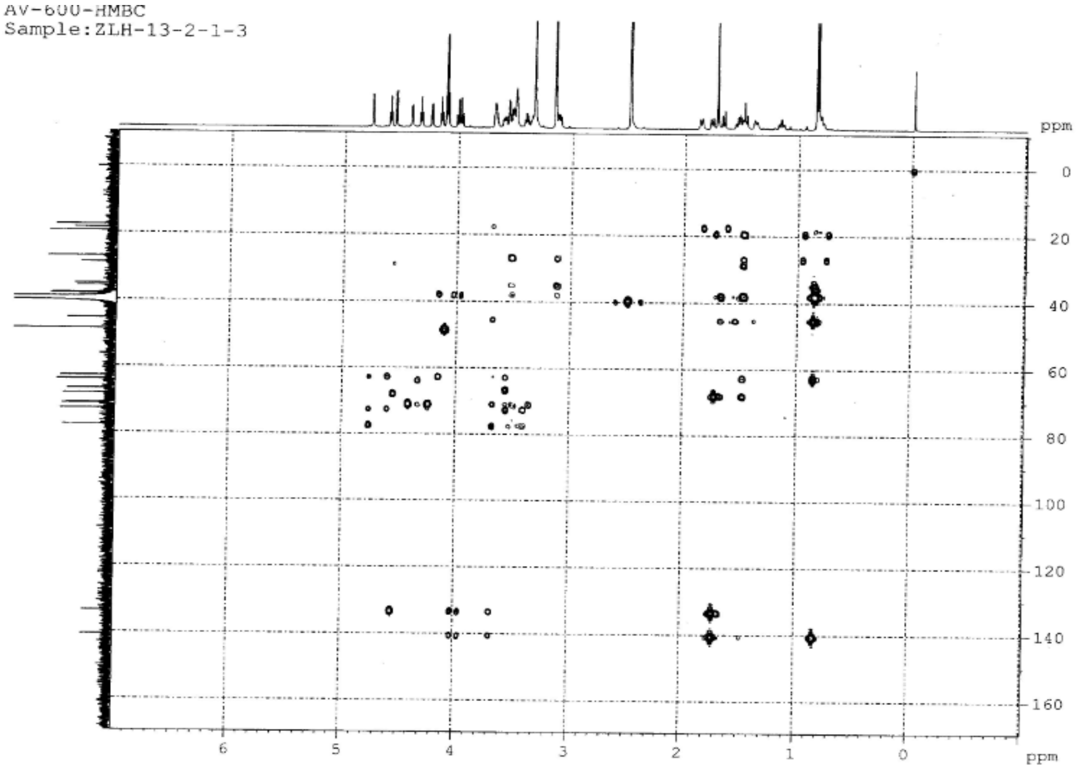

Figure S52. HMBC (600 MHz, DMSO-d_6) spectrum of compound 4

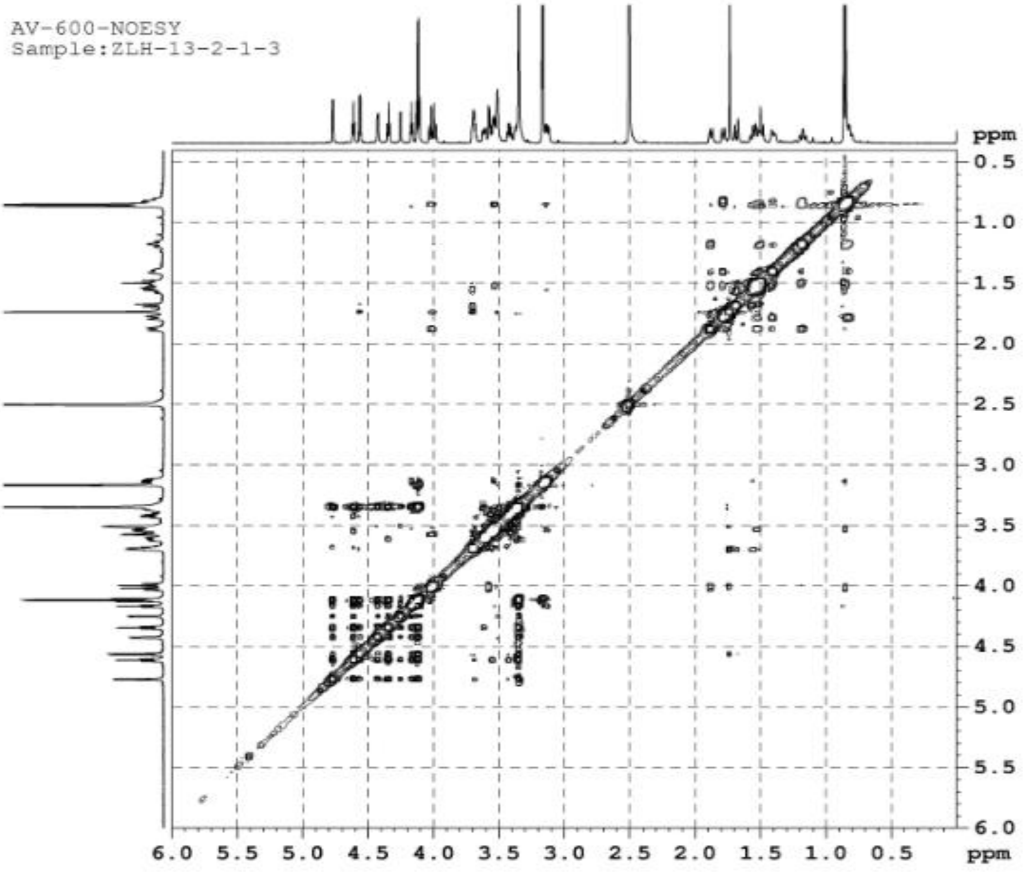

Figure S53. NOESY spectrum (600 MHz, DMSO-d_6) of compound 4

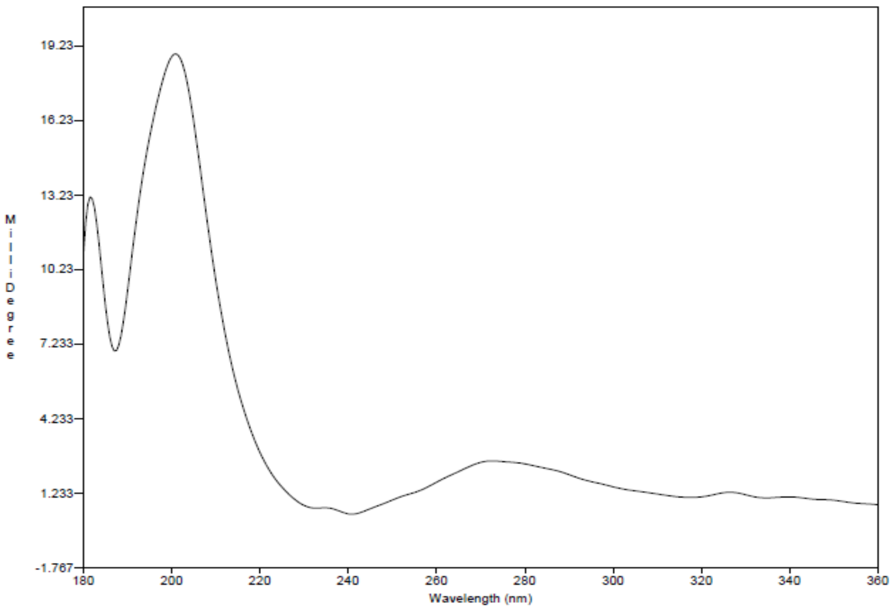

Figure S54. CD spectrum of compound 4 in CH_3CN

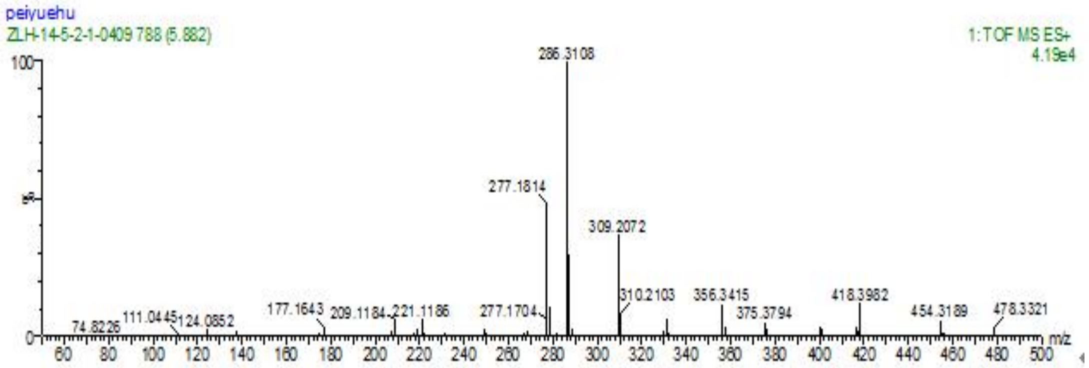

m/*z* 277.1814 [M + Na] $^{+}$

Figure S55. HR ESI-TOF MS spectrum of compound 5

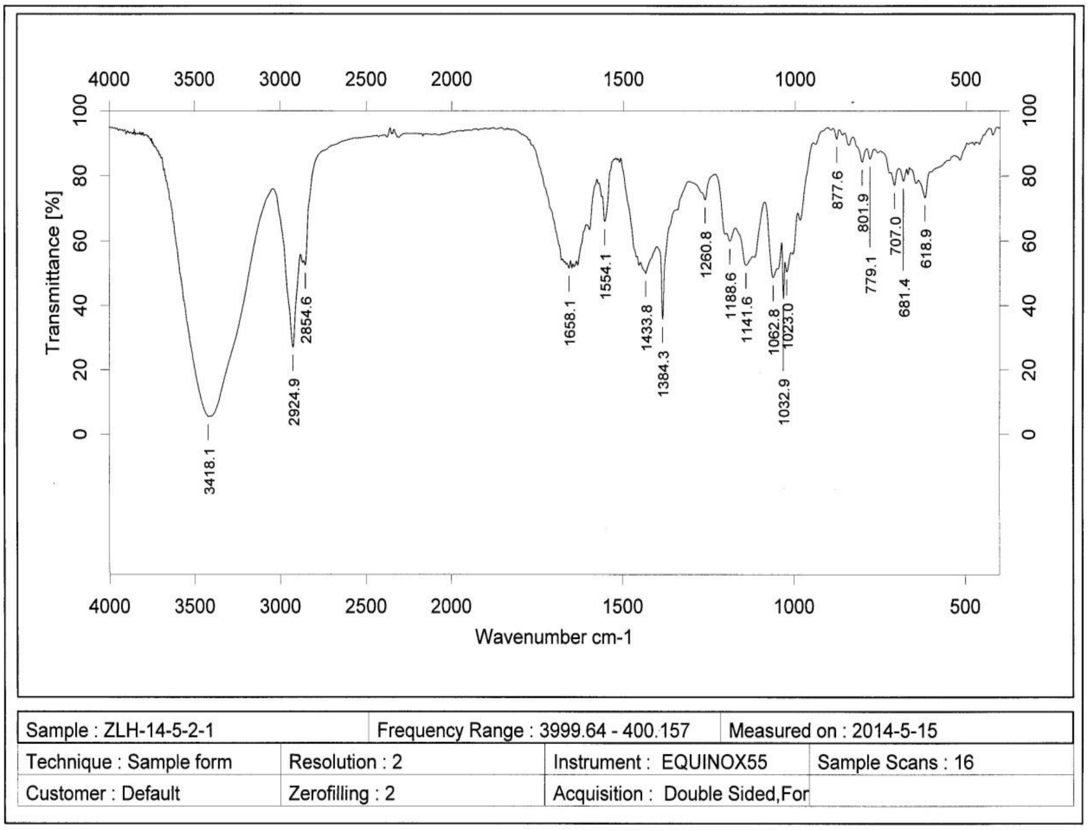

Figure S56. IR spectrum of compound 5

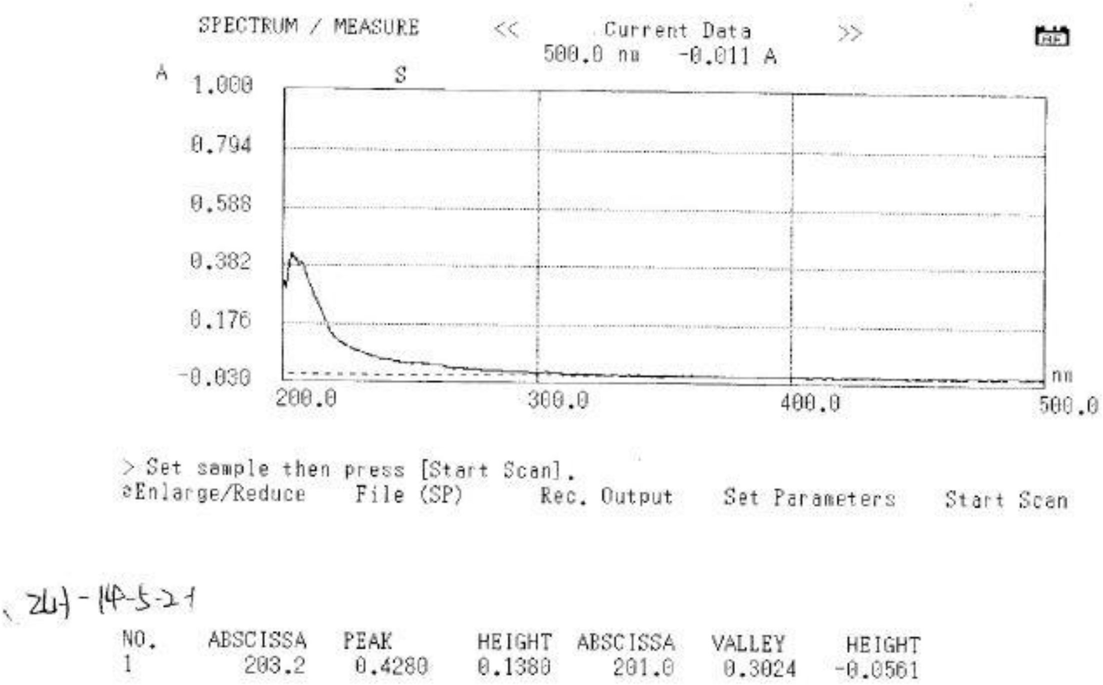

Figure S57. UV spectrum of compound 5

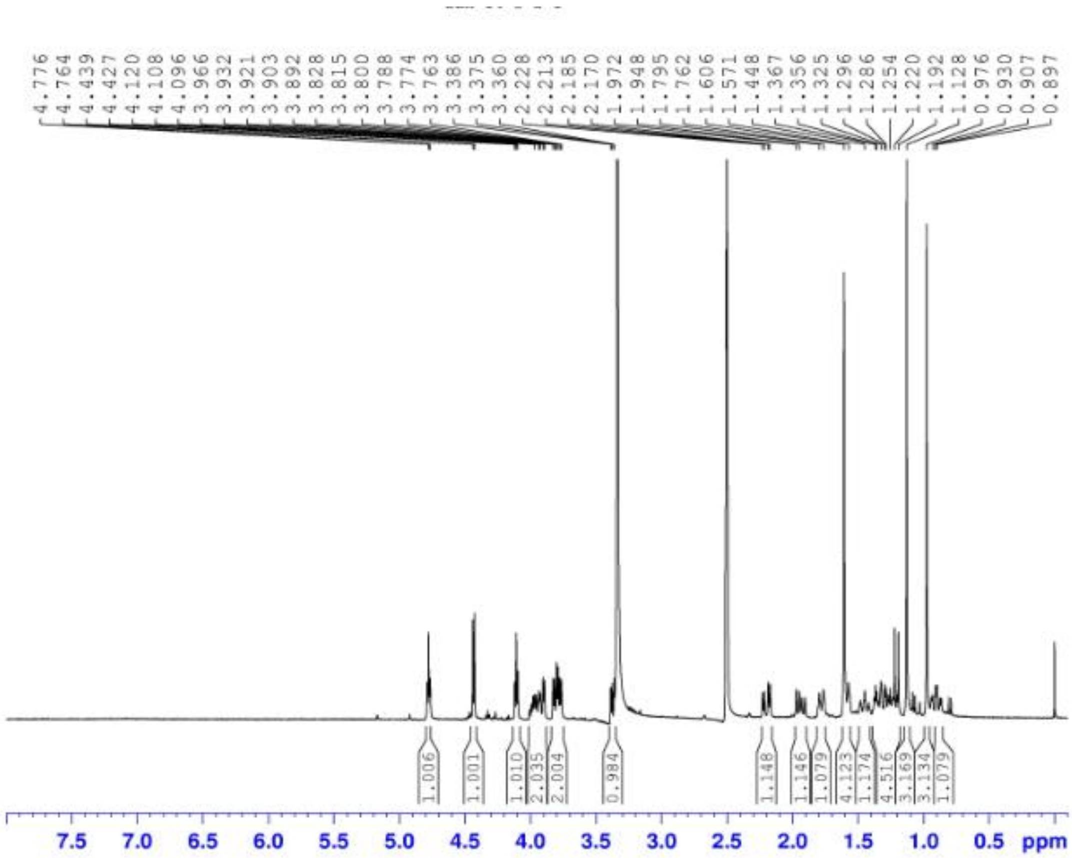

Figure S58. ^{1}H NMR (600 MHz, DMSO-d_6) spectrum of compound 5

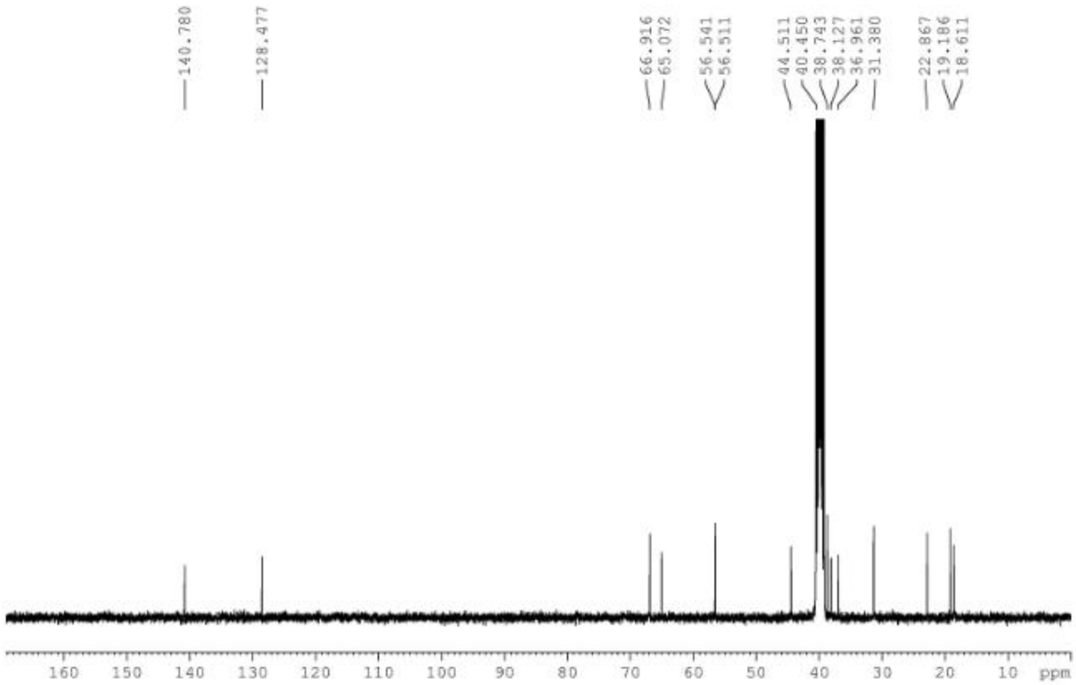

Figure S59. ^{13}C **NMR (100 MHz, DMSO-*d*$_6$) spectrum of compound 5**

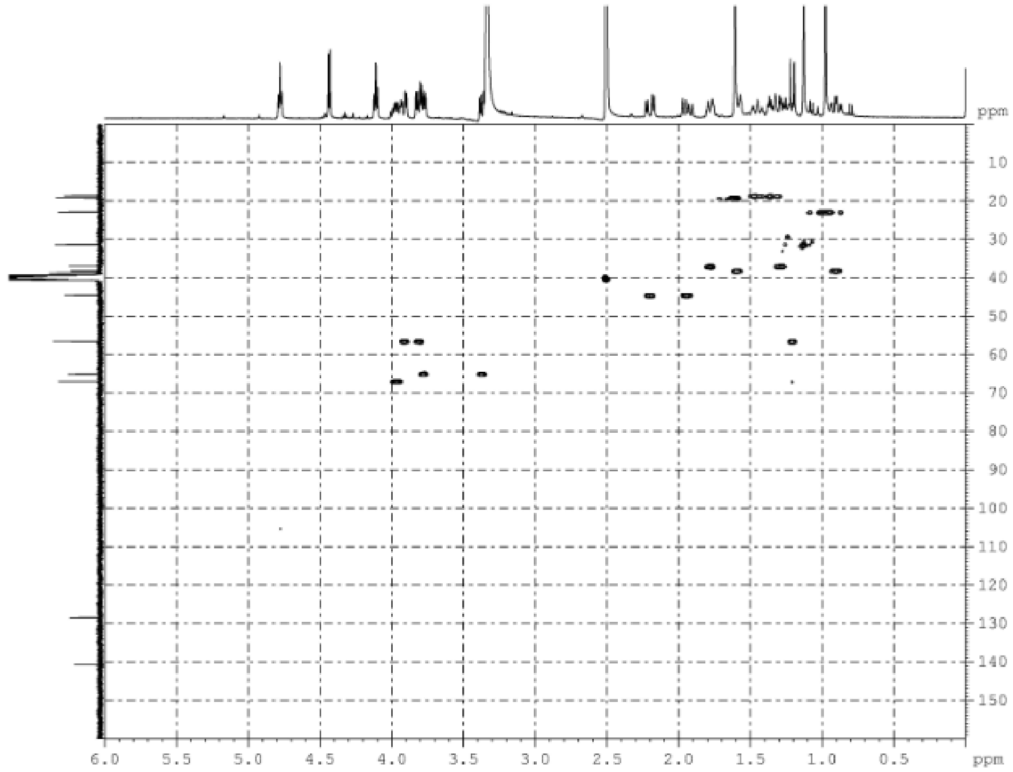

Figure S60. HSQC (600 MHz, DMSO-*d*$_6$) spectrum of compound 5

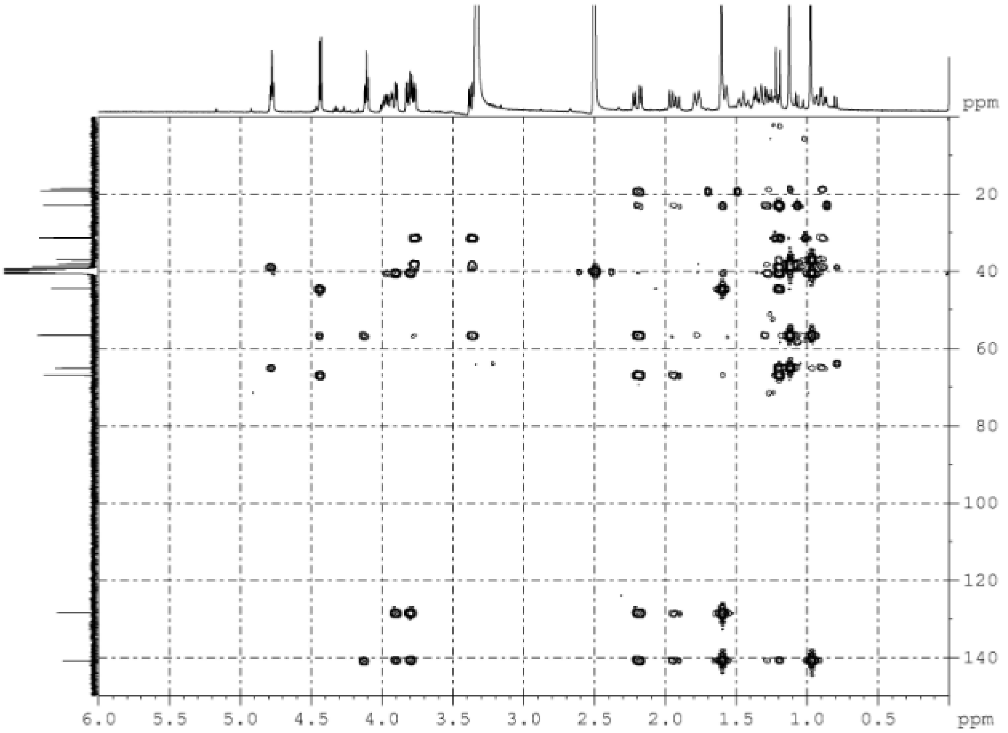

Figure S61. HMBC (600 MHz, DMSO-*d*6) spectrum of compound 5

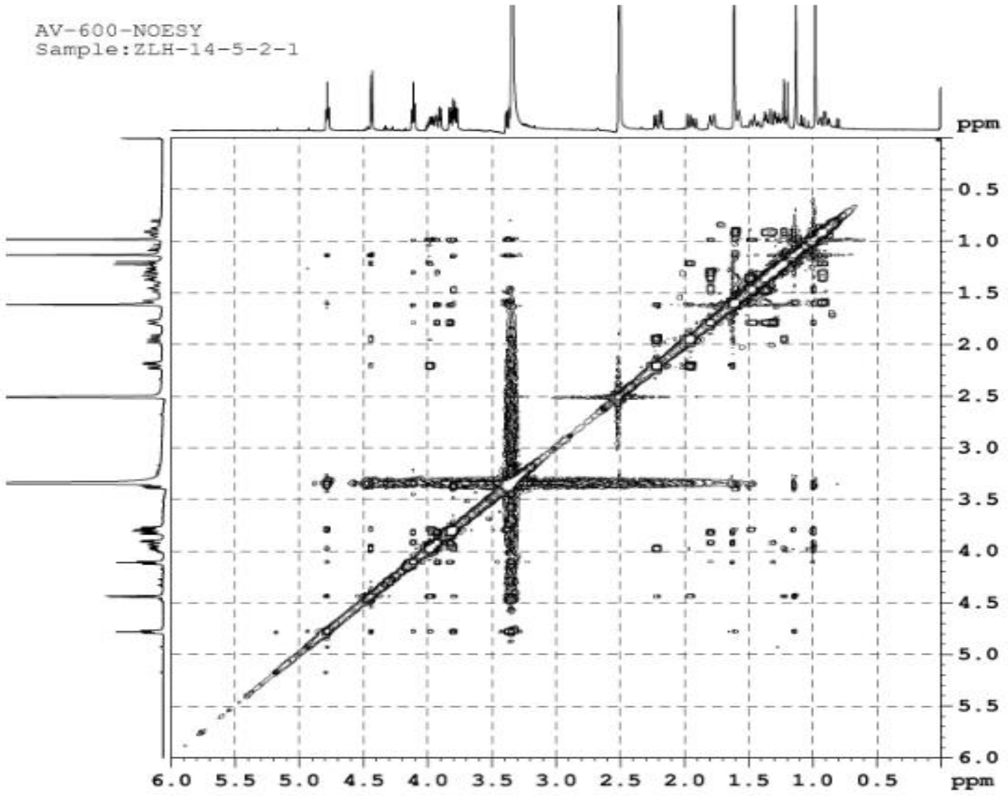

Figure S62. NOESY spectrum (600 MHz, DMSO-*d*6) of compound 5

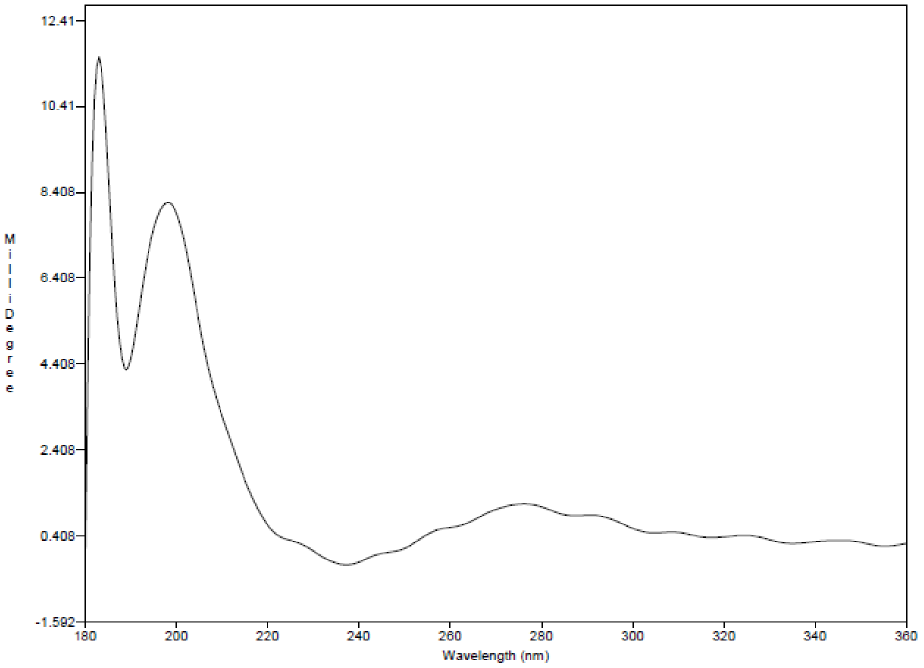

Figure S63. CD spectrum of compound 5 in CH_3CN

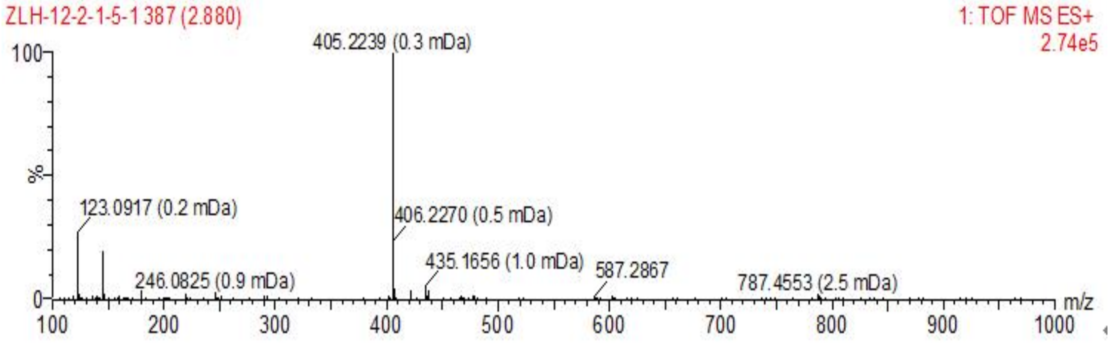

m/z 405.2239 [M + Na] $^{+}$

Figure S64. HR ESI-TOF MS spectrum of compound 6

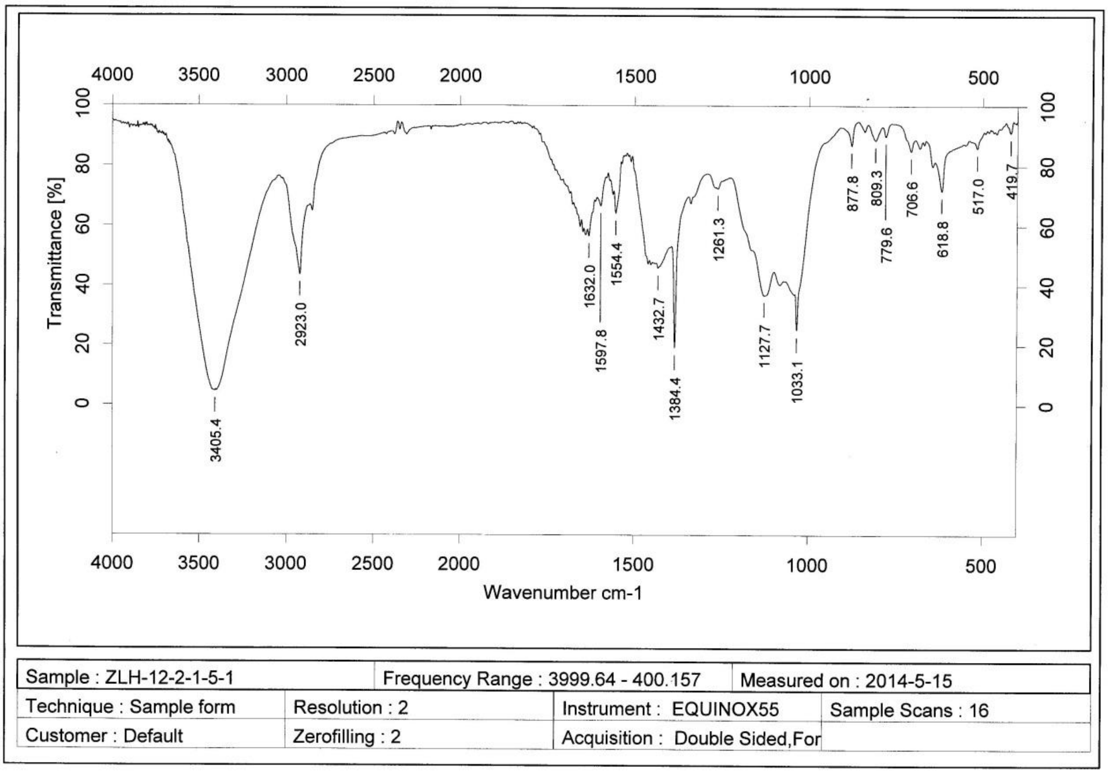

Figure S65. IR spectrum of compound 6

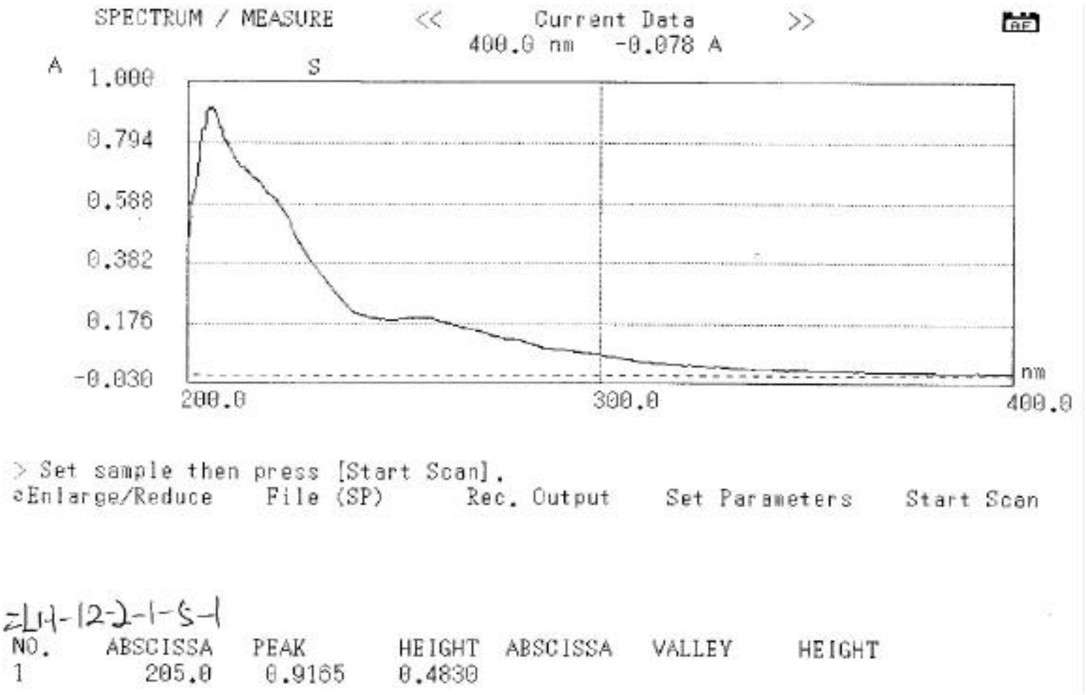

Figure S66. UV spectrum of compound 6

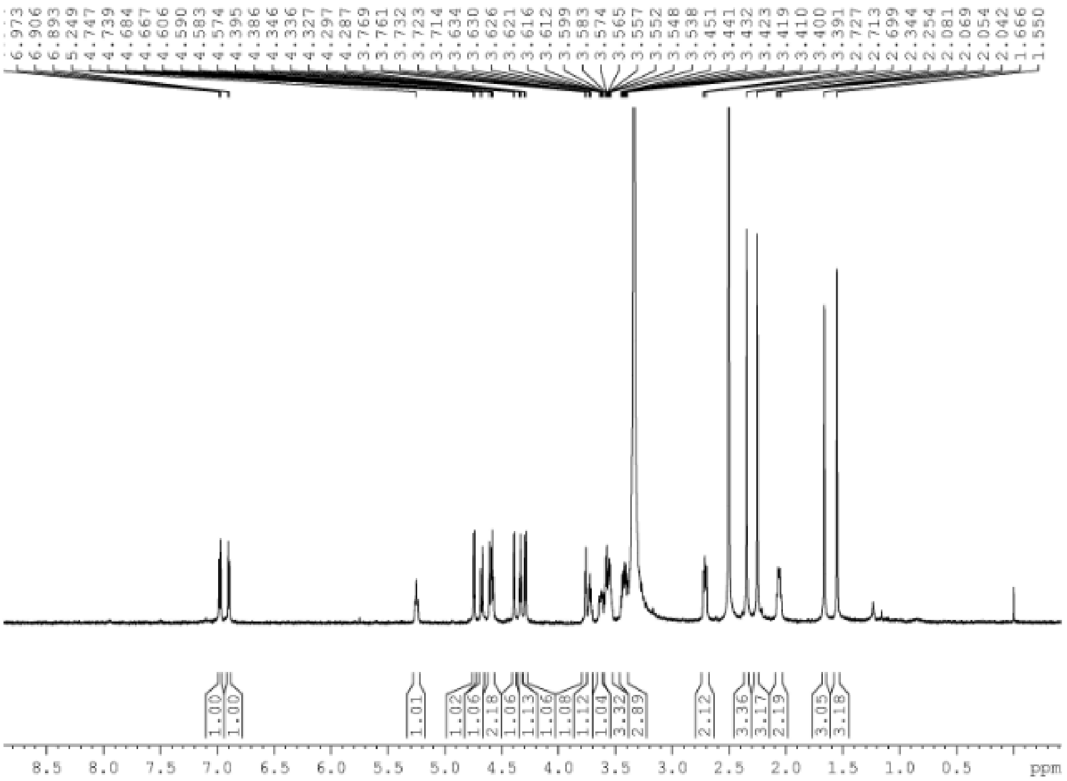

Figure S67. **¹H NMR (600 MHz, DMSO-*d*₆) spectrum of compound 6**

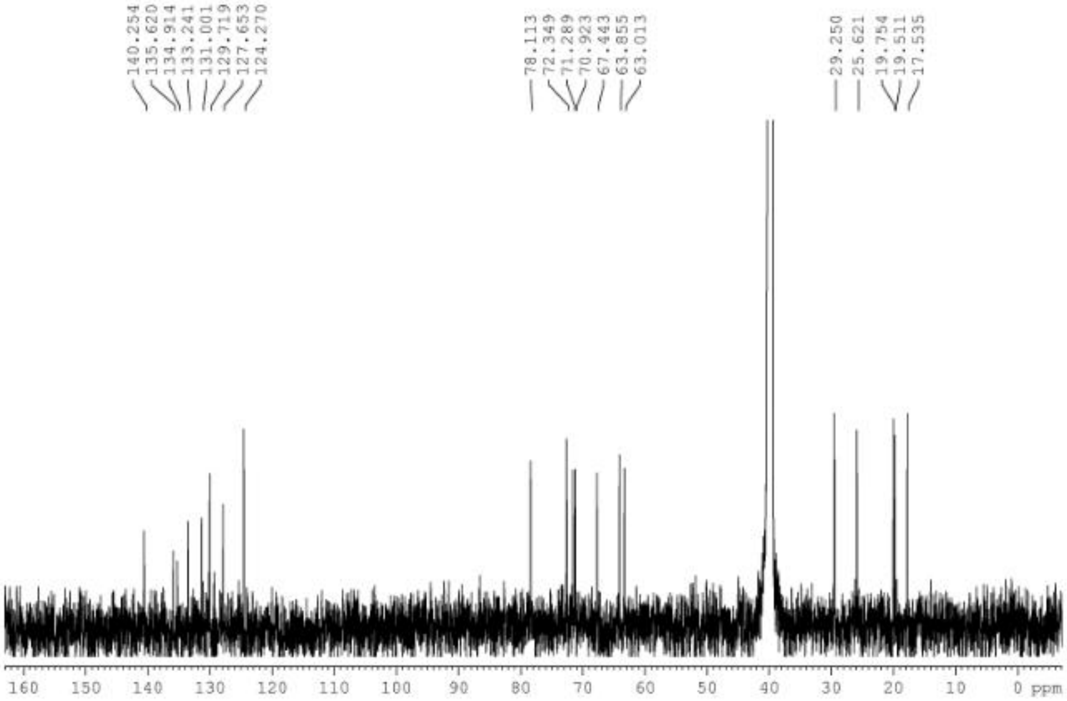

Figure S68. **¹³C NMR (100 MHz, DMSO-*d*₆) spectrum of compound 6**

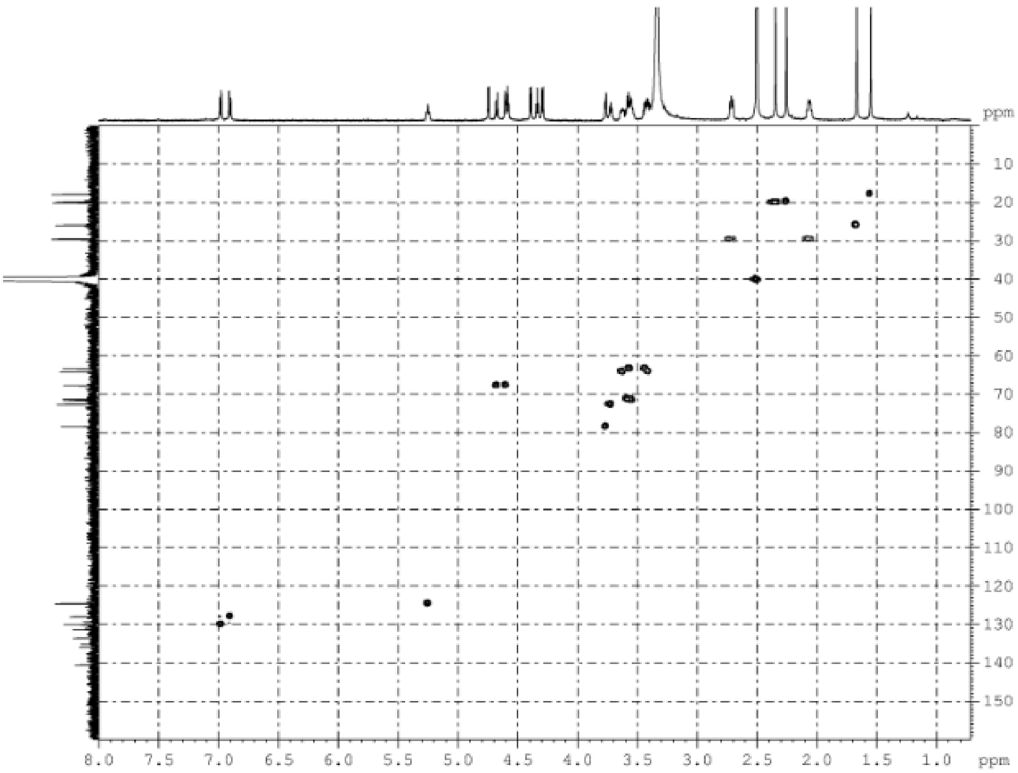

Figure S69. HSQC (600 MHz, DMSO-d_6) spectrum of compound 6

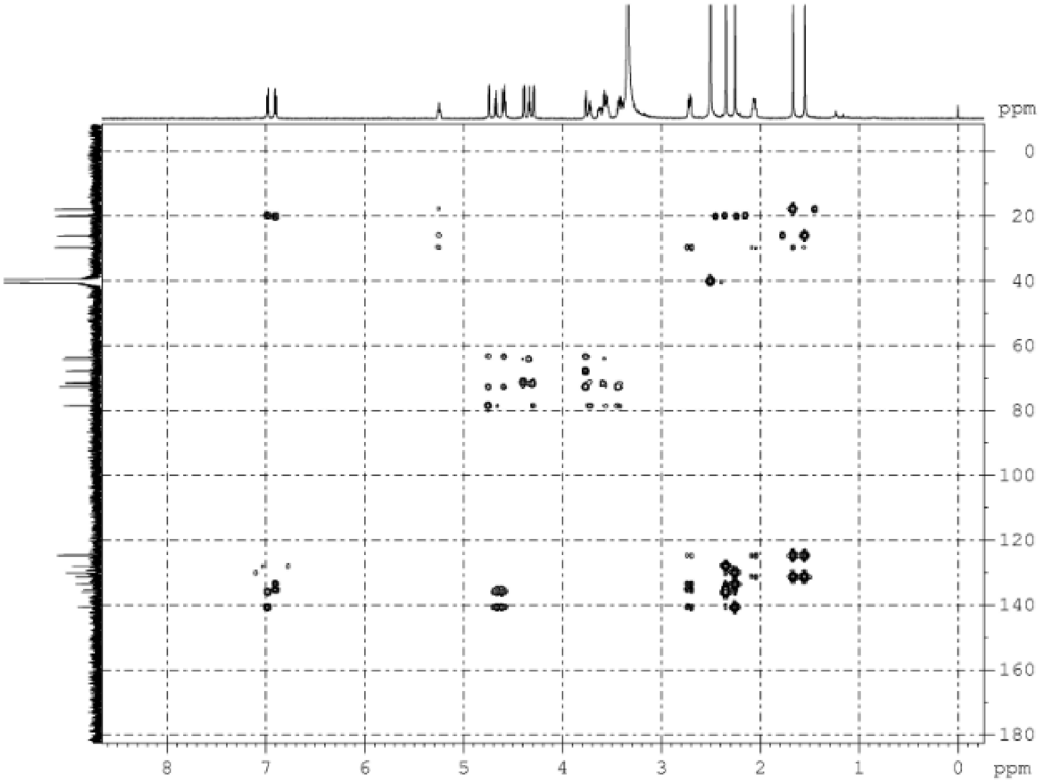

Figure S70. HMBC (600 MHz, DMSO-d_6) spectrum of compound 6

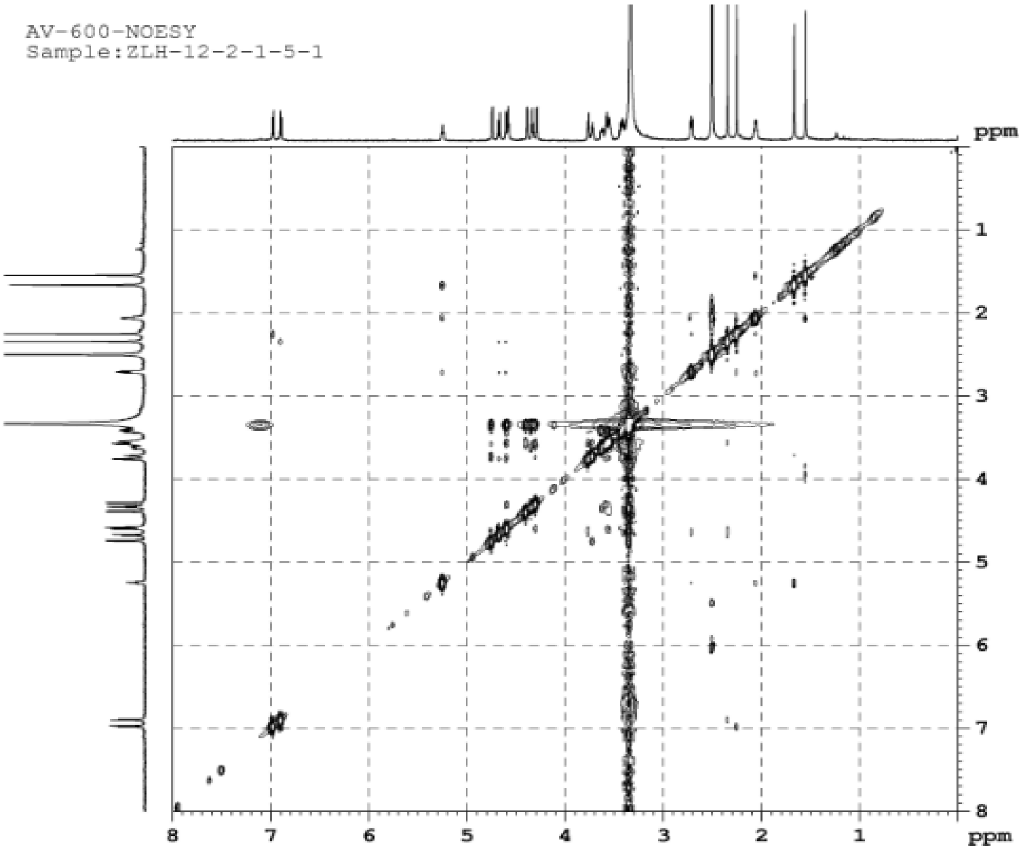

Figure S71. NOESY spectrum (600 MHz, DMSO-d_6) of compound 6

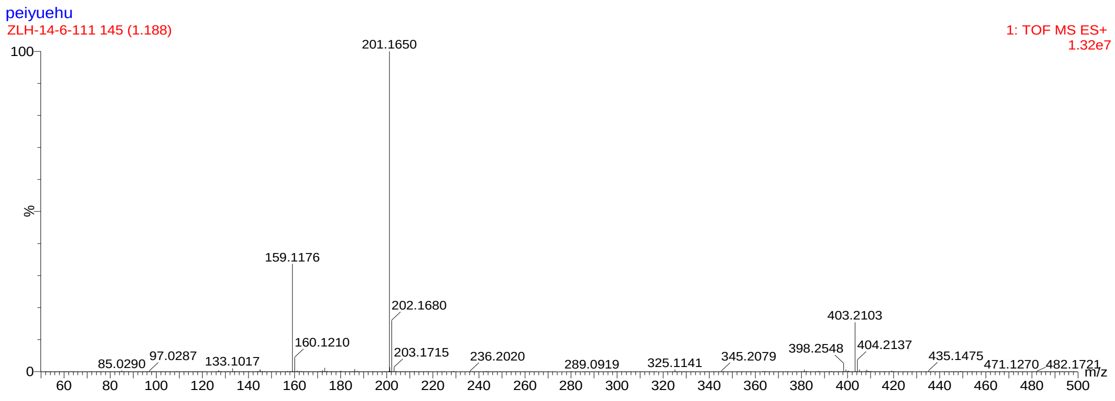

m/z 403.2103 [M + Na] +

Figure S72. HR ESI-TOF MS spectrum of compound 7

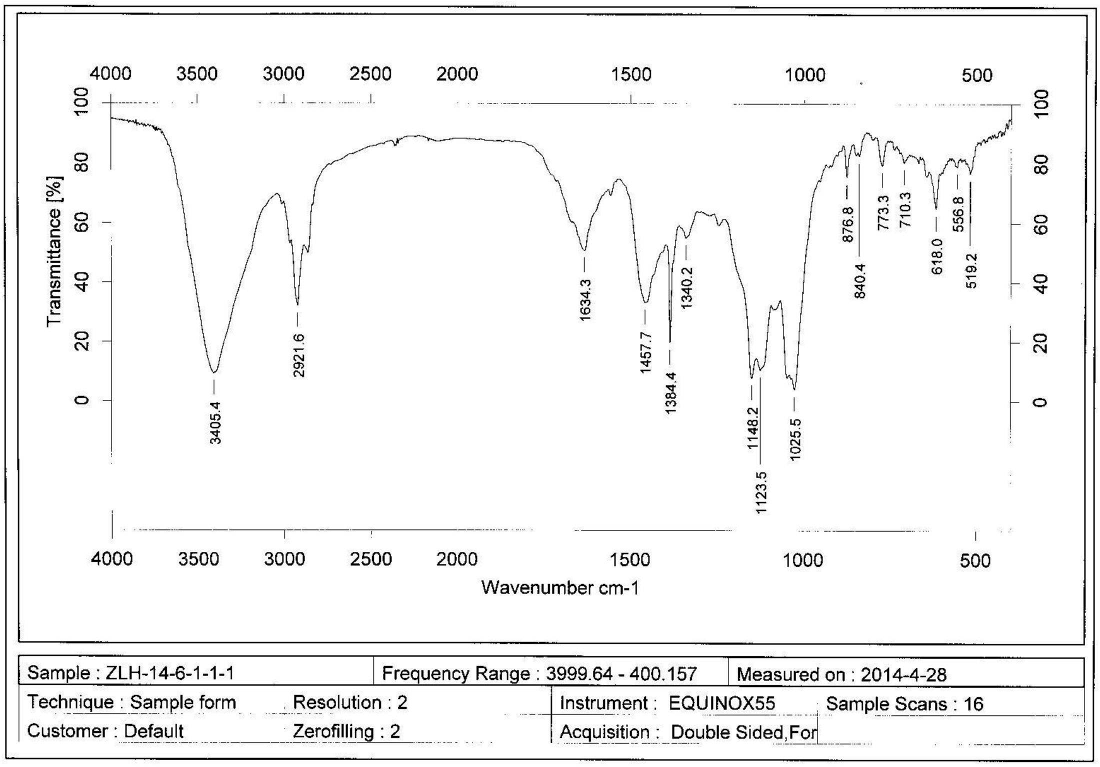

Figure S73. IR spectrum of compound 7

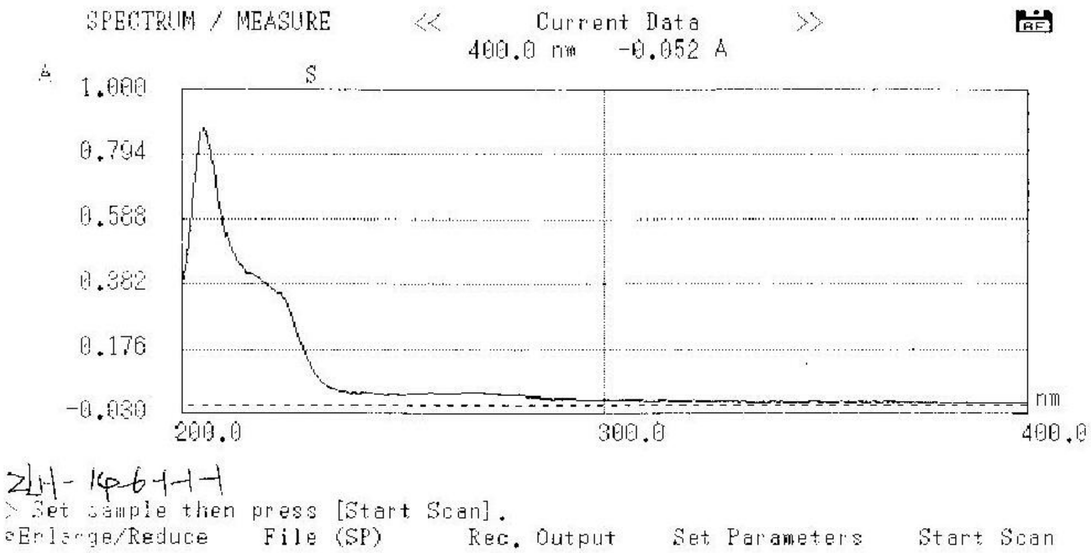

Figure S74. UV spectrum of compound 7

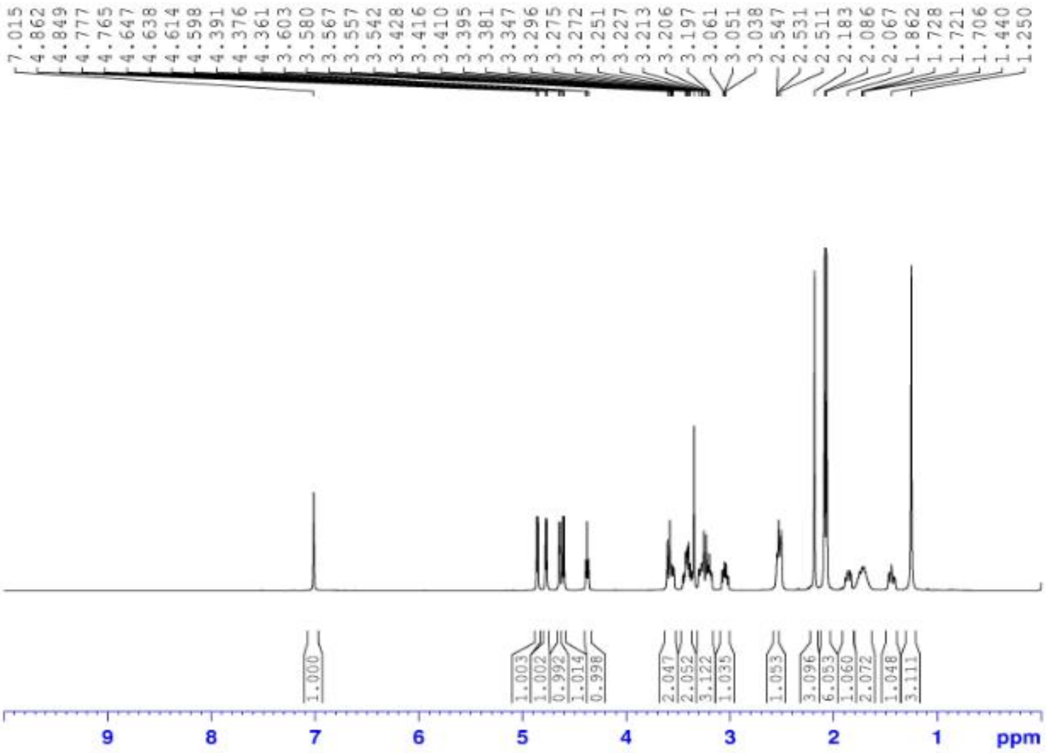

Figure S75. ^{1}H NMR (600 MHz, DMSO-d_6) spectrum of compound 7

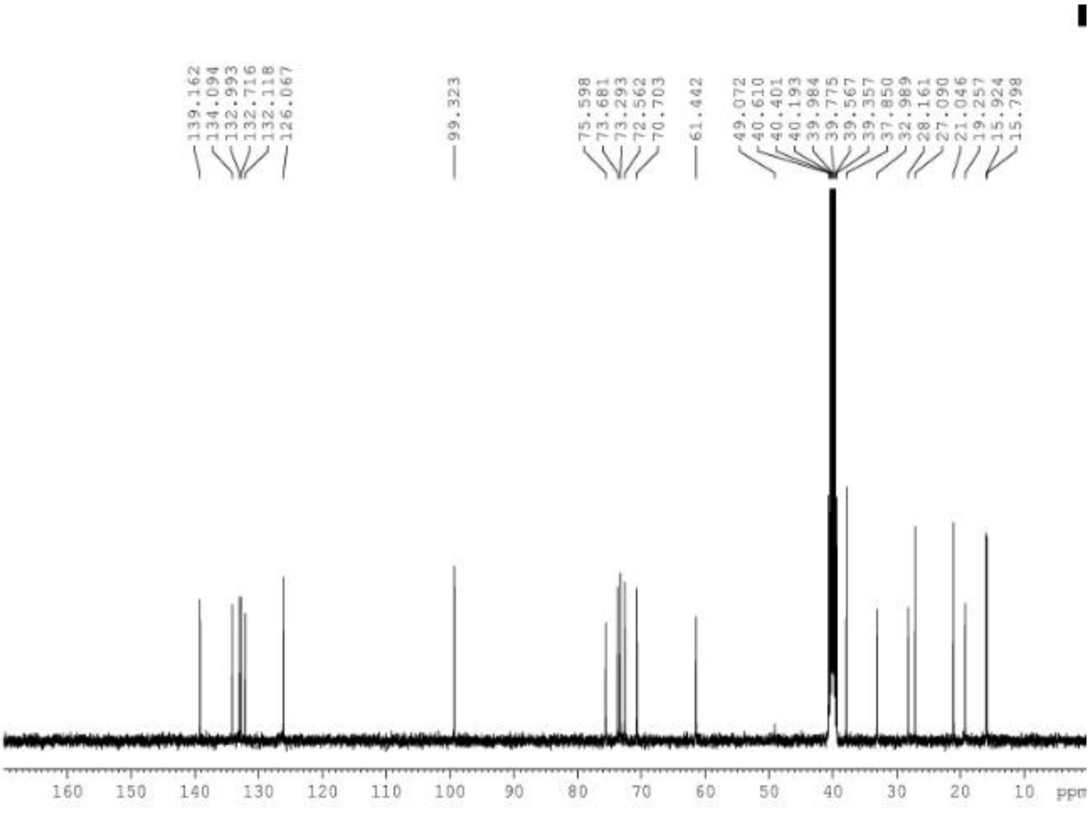

Figure S76. ^{13}C NMR (100 MHz, DMSO-d_6) spectrum of compound 7

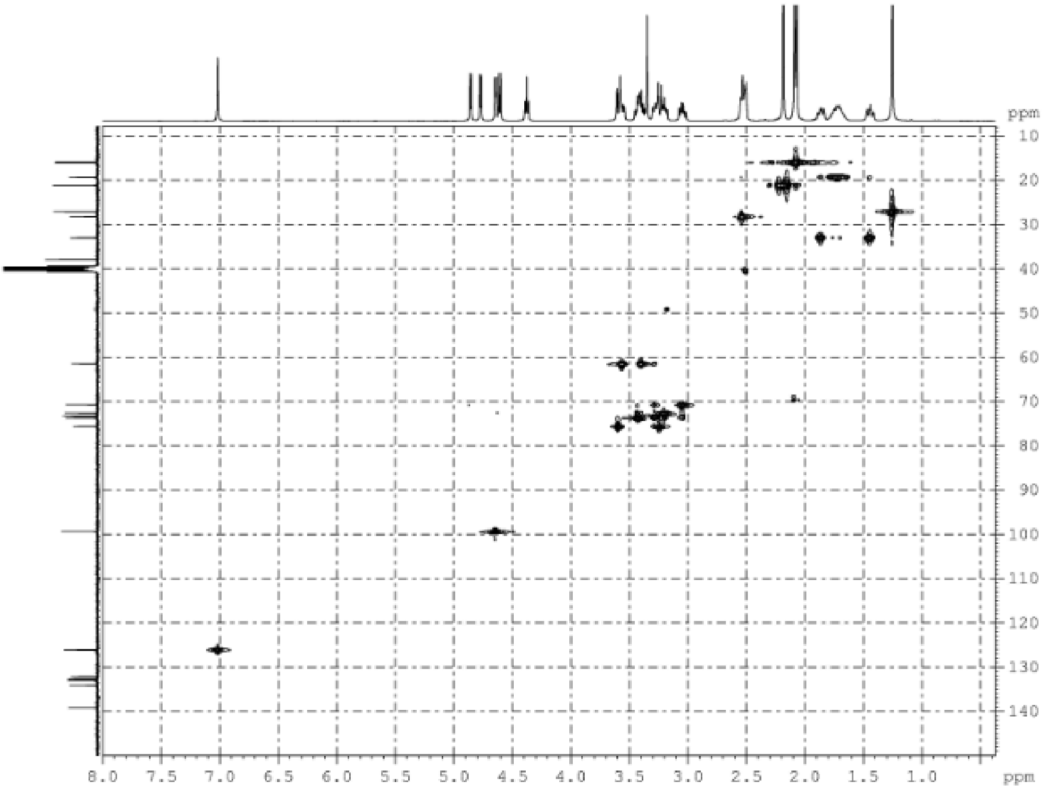

Figure S77. HSQC (600 MHz, DMSO-d_6) spectrum of compound 7

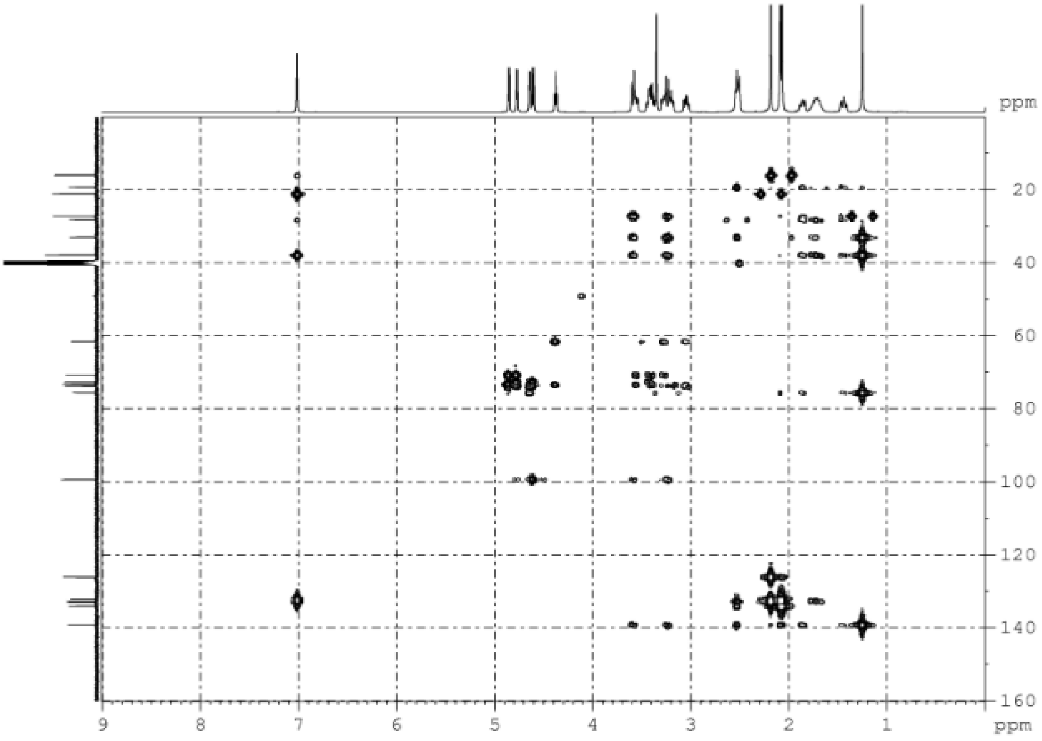

Figure S78. HMBC (600 MHz, DMSO-d_6) spectrum of compound 7

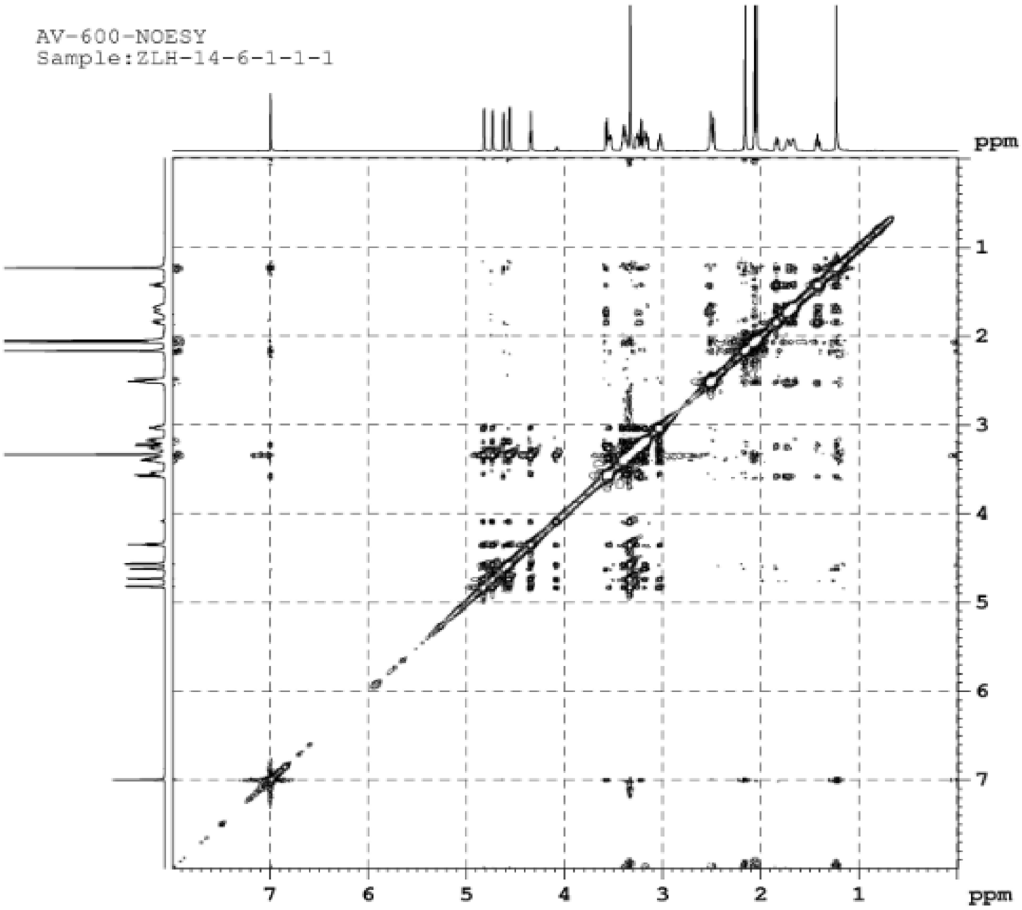

Figure S79. NOESY spectrum (600 MHz, DMSO-*d*6) of compound 7

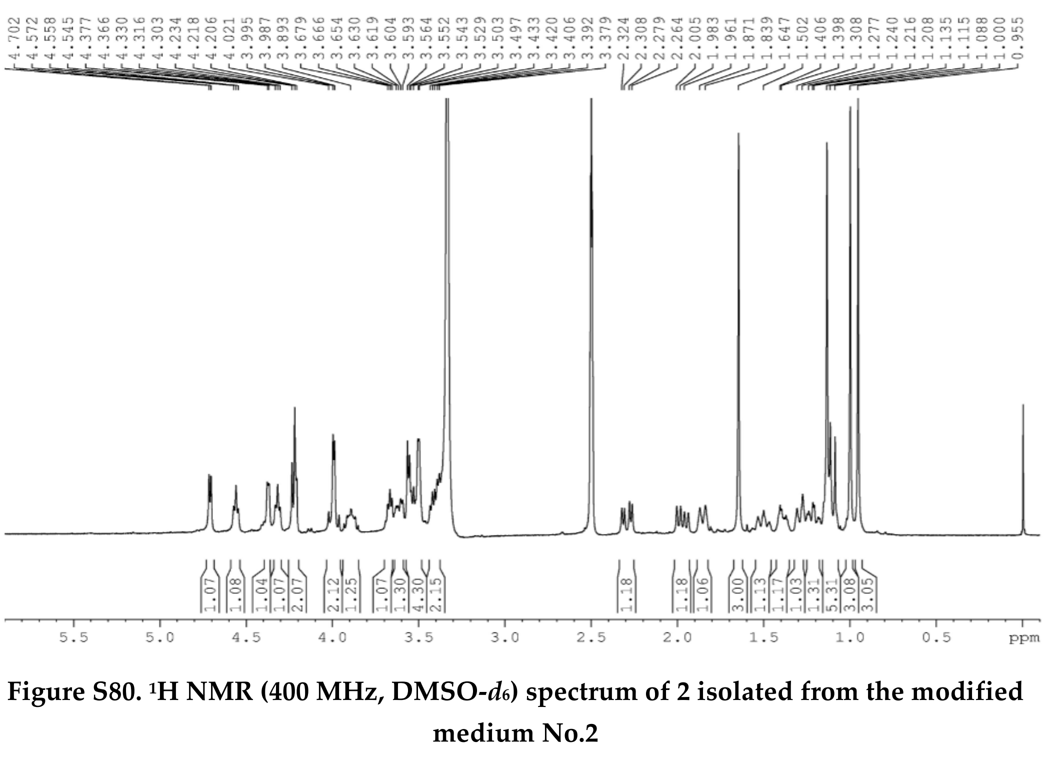

Figure S80. ¹H NMR (400 MHz, DMSO-*d*6) spectrum of 2 isolated from the modified medium No.2

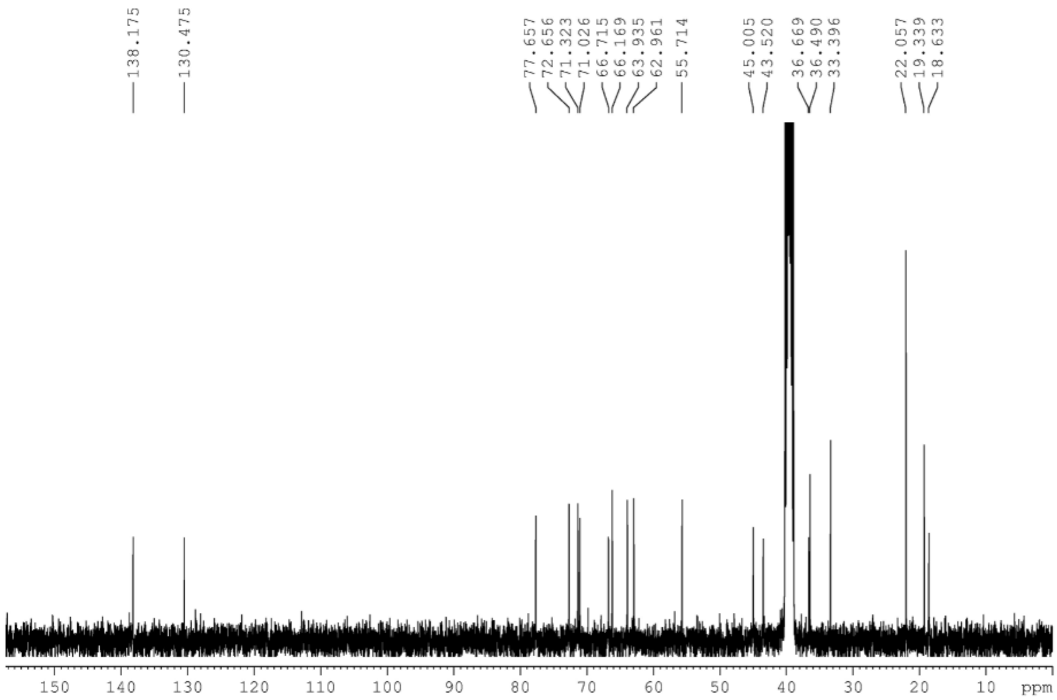

Figure S81. ^{13}C NMR (100 MHz, DMSO-d_6) spectrum of 2 isolated from the modified medium No.2
